# DeintensiF: Standard versus individualized deintensified follow-up after curative treatment in head and neck cancer: protocol of a randomized pilot study

**DOI:** 10.1186/s40814-025-01651-3

**Published:** 2025-05-16

**Authors:** M. Mueller, M. Visini, S. A. Mueller, T. Stadler, G. P. Rajan, G. B. Morand, S.-L. Hool, D. H. Schanne, P. Balermpas, A. Limacher, S. Chan, S. Trelle, O. Elicin, R. Giger

**Affiliations:** 1 Department of Otorhinolaryngology, Head and Neck Surgery, Inselspital, Bern University Hospital and University of Bern, Bern, Switzerland; 2 Private University in the Principality of Liechtenstein (UFL), Triesen, Liechtenstein; 3 Department of Otorhinolaryngology, Head and Neck Surgery, University Hospital Zurich and University of Zurich, Zurich, Switzerland; 4 Department of Otorhinolaryngology, Head and Neck Surgery, Lucerne Cantonal Hospital, Lucerne, Switzerland; 5 Department of Radiation-Oncology, Inselspital, Bern University Hospital and University of Bern, Bern, Switzerland; 6 Department of Radiation-Oncology, University Hospital Zurich and University of Zurich, Zurich, Switzerland; 7 Department of Clinical Research, University of Bern, Bern, Switzerland

**Keywords:** Follow-up studies, Head and neck neoplasms, Squamous cell carcinoma of head and neck, Neoplasm recurrence, Local, Recurrence, Precision medicine, Patient-reported outcome measures

## Abstract

**Background:**

Around 70% of head and neck cancer (HNC) cases are diagnosed in an advanced stage. Improvements in treatment have led to a cure rate of up to 80–90% for early-stage and 40–50% for advanced-stage disease. However, routine follow-up involves social and financial burdens, including frequent imaging associated with radiation exposure and costs. Currently, there is no consensus on the follow-up strategy after HNC treatment, and no conclusive evidence shows a survival advantage for routine follow-up over symptom-driven self-referrals. The DeintensiF study aims to provide robust evidence, comparing standard follow-up with a tailored deintensified approach. Additionally, it seeks to explore whether early detection of recurrence/second primary malignancy in asymptomatic patients impacts survival and quality of life. The pilot phase aims to assess feasibility of patients’ recruitment and adherence to the assigned follow-up strategy and patient-reported outcomes (PROs) questionnaire in the first 2 years.

**Methods:**

This randomized-controlled, multicenter, open-label, pilot study has the goal to randomize a minimum of 16 patients across three Swiss sites into two arms within 1 year. The Experimental Arm A: scheduled clinical exams every 6 months and monthly PRO with evaluation and possibility to alert for open urgent appointments; and the Control Arm B: regular visits every 3 months for the first 2 years and less frequent thereafter plus multiple scheduled imaging appointments for head and neck magnet resonance imaging (MRI) and computed tomography (CT) with contrast and chest CT scans. Patients’ motivation for participation or not will be explored by additional questionnaire before randomization. The primary objective during the pilot phase is to evaluate the feasibility of recruiting and randomizing patients with complete remission 6 months after treatment of head and neck squamous cell carcinoma to a deintensified and to a conventional follow-up. The secondary objective is to assess adherence to the two different follow-up strategies.

**Discussion:**

If feasible, the DeintensiF pilot study will expand from the recruited patients (detailed in the “Methods” section) to a larger cohort of advanced HNC cases in the main trial, integrating electronic PRO tailored follow-up care. This approach aims to reshape follow-up practices, enhancing patient-centered strategies and outcomes in head and neck oncology.

**Trial-registration:**

ClinicalTrials.gov (NCT05388136); Swiss National Clinical Trial Portal (SNCTP000005198).

**Supplementary Information:**

The online version contains supplementary material available at 10.1186/s40814-025-01651-3.

## Background and rationale

Head and neck squamous cell carcinoma (HNSCC) are the 6 th most common type of non-skin cancer [1]. Tobacco, alcohol, and human papillomavirus (HPV) are the main risk factors. Treatment options vary from surgery to radiotherapy (RT), either alone or combined with chemotherapy (CXRT), tailored to cancer site and stage. Early-stage HNSCC (stages I–II, 30% of cases) typically receive single-modality treatment, while advanced cases (stages III–IVA/B, 70% of cases) often necessitate multimodal approaches, such as CXRT or surgery followed by adjuvant (CX)RT. Cure rates for early-stage cancer reach 80–95%, yet locoregional recurrence (REC) affects 50–60% of advanced cases within 2 years, with 20–30% developing distant metastases. Additionally, there is a + 2–4%/year risk of second primary malignancies (SPM), mainly in the lung (60%) and superior aero-digestive tract (20%). HPV-positive oropharyngeal cancer patients exhibit lower REC/SPM risks [2–9]. The 5-year overall survival rates for HNSCC patients treated between 2002 and 2006 averaged 65.9% [10]. Despite treatment successes, patients face treatment-related morbidities, impacting their quality of life (QoL), necessitating comprehensive follow-up (FU) protocols. Curative setting patients’ FU in this population includes treatment response evaluation, REC/SPM detection, management of treatment sequelae, nutritional restoration, and psychosocial support [11]. Unnecessary investigations that may cause morbidity, discomfort, or stress and may add financial burden on the patient need to be avoided. There is neither consensus nor level one evidence on how to ideally achieve these globally accepted FU goals. Guidelines recommend routine clinical visits for head and neck examination, assessing radiation-induced toxicity and functional rehabilitation [12]. Following baseline imaging (magnet resonance imaging (MRI) and computed tomography (CT)) within 3–4 months post-surgery or after definitive CXRT, long-term reimaging until and beyond 5 years in asymptomatic patients remains controversial and unexplored. Within 3–6 months post-CXRT, a hybrid positron emission tomography CT or MRI (PET) is recommended for treatment response assessment and detection of tumor persistence/progression [13]. A chest CT scan should be performed if chest imaging is requested (chest X-ray detects only 33% of intra-thoracic lesions visualized by chest CT) [14]. Existing FU approaches lack consensus, varying in visit frequency, imaging, and duration, as listed in Table 1 [15–25].

**Table 1:** Published guidelines on FU intervals

Period	Year 1	Year 2	Year 3	Year 4	Year 5	> 5 years
*Guidelines*						
ASHNS (ASHNS, 1999) [15]	1–3	2–4	3–6	4–6	4–6	12
BAHNO (BAHNO, 2001) [16]	1–1.5	1–1.5	3	6	6	12
DCHNO (DCHNO, 2002) [17]	2	3	4	6	6	–
Lester and Wight, 2009 [18]	1	2	3	4	6	12
Digonnet et al., 2013 [12]	2–3	2–3	3–6	3–6	6	12
AIMO/AIRO (AIMO/AIRO, 2016) [19, 20]	1–3	3	3–6	6	6	12
FOS (FOS, 2016) [21]	2	3	4	6	6	12
SSORL (SSORL, 2019) [22]	1–3	1–3	4–6	4–6	4–6	(12)
EHNS/ESMO/ESTRO (EHNS/ESMO/ESTRO, 2020) [23]	2–3	2–3	6	6	6	12
NCCN (NCCN, 2024) [24]	1–3	2–6	4–8	4–8	4–8	12

Suggested interval of FU visits in months by selected authors and national committees

*AIMO* Associazione Italiana Oncologia Medica, *AIRO* Associazione Italiana Radioterapia Oncologica, *ASHNS* American Society of Head and Neck Surgeons, *BAHNO* British Association of Head and Neck Oncologists, *DCHNO* Dutch Cooperative Head and Neck Oncology Group, *EHNS* European Head and Neck Society, *ESMO* European Society for Medical Oncology, *ESTRO* European Society for Radiotherapy and Oncology, *FOS* French ORL Society, *NCCN* National Comprehensive Cancer Network, *SSORL* Swiss Society of Oto-Rhino-Laryngology, Head and Neck Surgery

Routine surveillance has shown survival benefits in two retrospective studies for REC diagnosed during scheduled FU visits compared to self-referral [26, 27]. However, many other retrospective studies found no difference in overall survival (OS) between routine FU and symptom-driven self-referral, indicating that patients efficiently detect symptomatic REC while silent RECs are rare [28–42]. Additionally, electronic Patient-Reported Outcomes (ePROs) enable real-time symptom monitoring and may offer a survival benefit. Basch et al. (2016) demonstrated that web-based ePROs significantly enhanced 1-year survival rates for advanced solid cancer patients [43]. Another study showed that ePROs during chemotherapy for advanced lung cancer increased 2-year survival, surpassing standard post-treatment imaging for REC detection [44]. With limited evidence on post-treatment FU for HNSCC patients, a multicenter trial comparing deintensified and conventional FU strategies is crucial to establish a cost-efficient, patient-centered approach.

The DeintensiF main trial hypothesizes that a patient-centered deintensified and individualized FU strategy is non-inferior compared to a standard FU approach. The specific primary aim of the DeintensiF trial is to determine whether the new approach with enhanced patient involvement actually reduces the number of outpatient visits without compromising survival compared to a standard FU with fixed scheduled clinical exams and imaging in curatively treated HNSCC patients. Secondary objectives relate to investigating additional clinical and economic effects of the deintensified individualized FU scheme. In addition, we aim to disentangle underlying mechanisms and to describe implementation aspects including acceptance and patient views. For the full protocol, see Additional file 1: DeintensiF-Full Protocol.

The general aims of the DeintensiF pilot phase are to evaluate the feasibility of recruiting and randomizing patients with complete remission 6 months after treatment of HNSCC to a deintensified or conventional (standard) FU. The secondary objective is to investigate adherence to the two different FU strategies. Good compliance is defined as the realization of the planned/organized visits and exams at the planned time ± 4 weeks as well as regularly recording of signs and symptoms in the PROs questionnaire.

## Methods

### Trial design

**Fig. 1:**
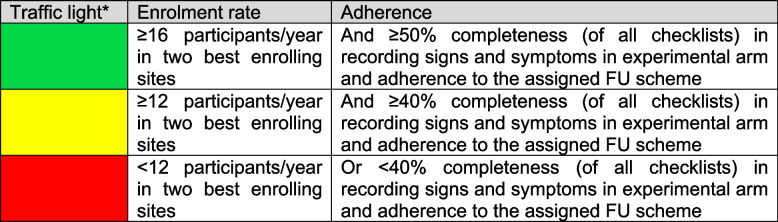
Traffic light system for moving from the pilot phase to the main phase. *Green: continue (i.e., no concerning issues that threaten the success of the trial); yellow: either adapt or continue with caution (i.e., remediable issues); red: stop or at least halt (i.e., intractable issues that cannot easily be remedied). Although it is foreseen to have only two to three sites in the pilot, using enrolment in the two best enrolling sites allows also for more sites in the pilot

Patients fulfilling the eligibility criteria are randomized to one of two FU schedules using a 1:1 ratio. The study also investigates patients'adherence to the assigned protocol and collects additional data on patients’ motivation for study participation. Three Swiss academic tertiary referral centers in Bern, Lucerne and Zurich are involved in this initial phase. During the pilot phase, the sites committed to enroll a total of 20 patients within the first year. This phase serves as a crucial precursor to the main trial, providing insights into the practicality and effectiveness of the deintensified FU strategy compared to the standard approach. The pilot study differs from the main study by collecting additional data concerning patients’ motivation for study participation and the use of paper-based symptom reporting in the experimental arm (as opposed to an electronic patient-reported outcome data capture system). Otherwise, all procedures mimic the main trial. Data from this pilot study will be used to inform the planned large-scale main trial. Decisions will be based on a traffic light system (Fig. 1) before implementation of the larger-scale main trial, demonstrating its feasibility and relevance in addressing the challenges of post-treatment FU in HNSCC patients [45].

**Fig. 2:**
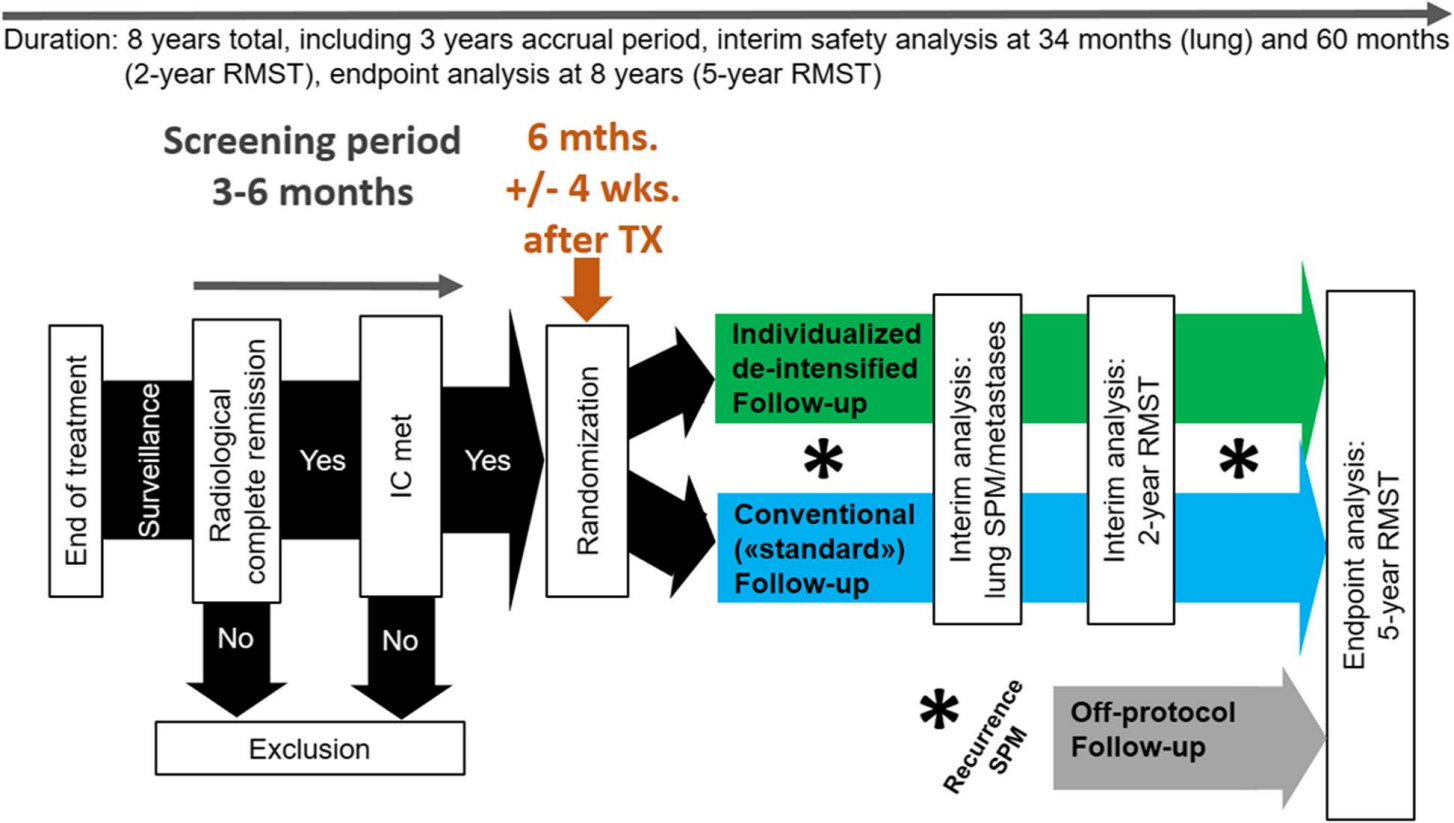
Flow chart of participants’ recruitment and study procedure of the main trial. Abbreviations: IC = inclusion criteria; mths. = months; RMST = restricted mean survival time; SPM = second primary malignancy; TX = treatment; wks. = weeks

The success of the pilot study and its progression to the main trial depend on observed enrolment rates and adherence to the FU protocol, as demonstrated in Fig. 2. The criteria for advancing to the main trial are based on the number of sites planned for participation, the size and duration of participation of those sites, and an expected enrolment of 550 patients within 3 years for the main trial.

### Objectives and success criteria of the pilot study

#### Primary objective

The primary objective is to assess the feasibility of recruiting and randomizing HNSCC patients in complete remission 6 months post-treatment to either a deintensified or a conventional FU protocol. The aim is to recruit at least 80% of the committed 20 patients (*n* = 16) within the initial 1-year recruitment period, ensuring that enrollment remains on track to achieve sample size and timeline goals.

#### Secondary objective

The secondary objective is to investigate adherence to the two FU strategies, defined as the completion of scheduled visits and exams as planned, along with regular recording of signs and symptoms in the patient-reported outcomes (PRO) questionnaire. Good compliance is defined as the realization of the planned/organized visits and exams at the planned time ± 4 weeks as well as regularly recording of signs and symptoms in the PRO questionnaire. A retention target of 90% is set for those completing scheduled FU assessments, alongside an adherence goal of 85% to the FU schedule. To ensure the reliability of the findings, at least 95% completeness is aimed in required data fields, with data audits conducted by the Clinical Trials Unit at the University of Bern.

#### Endpoints of the pilot study

Eligibility rate: proportion of all eligible patients out of all screened patients. Consent rate: Proportion of consenting patients among all eligible patients. Patient motivation for participation based on a participation questionnaire. Overall accrual per month and adherence to the randomized FU schedule.

Meeting the targets will confirm the feasibility of moving forward with the main trial. If any challenges arise in recruitment, retention, or data quality, insights from the pilot phase are used to adjust and optimize the approach.

### Study procedure

#### Participants and recruitment

Inclusion criteria.

Participants must be aged 18 or older, have a histopathologically proven invasive HNSCC at clinical/radiological stage II–IV (non-surgically treated) or pathological stage II–IV (surgically treated, excluding M1) according to the Union for International Cancer Control (UICC) tumor, node, metastasis (TNM) UICC/TNM 8 th edition. Other inclusion criteria are curative treatment intent, planned FU at the study center, radiological and clinical confirmation of complete remission, and agreement for 5-year FU. Furthermore, signed, written informed consent is required.

Exclusion criteria.

Participants with initial clinical stage I and/or M1 HNSCC, nasopharyngeal cancer or carcinoma of unknown primary, previously treated head and neck cancer (except specified cases), other synchronous or metachronous malignancies (except specified cases), participation in conflicting studies, pregnant or breastfeeding women, and those with conditions affecting protocol compliance will be excluded [46].

For detailed criteria and procedures, refer to the full protocol submitted as supplementary material (see Additional file 1: DeintensiF-Full Protocol).

#### Recruitment procedure

Patients receive study information through a recruitment flyer and complete a participation questionnaire from the treating centers. Patient registration and randomization are performed online in secuTrial®, with eligibility checks and signed consent obtained.

#### Randomization and stratification

Following the entry of baseline information and eligibility criteria, participants will be randomized in secuTrial®. They will be assigned to either the experimental (individualized deintensified FU) or control (conventional FU) arm in a 1:1 ratio. Stratification is done via probabilistic minimization to balance prognostic factors across arms [47, 48]. Stratification factors include tumor stage, treatment modality and trial site.

#### Study intervention

Patients in the experimental arm will have scheduled outpatient visits every 6 months including a clinical examination. In-between, patients complete a monitoring questionnaire monthly, with alerts triggering additional visits, if necessary. Details of the instrument are shown in Table 2. Participants will receive reminders to complete the PROs questionnaire the day before the expected date with up to three reminders in the main study, if missed. During the pilot study, phone calls are conducted if the PROs questionnaire is completely or partially missing 1 day after the planned date, which mimics the automated alert by the app in the future main study. In the main trial, a phone call will also follow 3 days after the app’s notification, in case the questionnaire is still not completed. Patients not able to adequately use the app will be offered a paper-based version of the PROs questionnaire, similar to the pilot study. The use of a paper-based PRO questionnaire in the pilot study is a logistical choice intended to simulate the future electronic alert system. This change is solely for feasibility assessment, and any potential differences in data collection methods will be controlled in subsequent subgroup analyses. If a patient fails to complete the PRO, up to two calls are made within 1 week to gather the results. Depending on the rating, the PRO will trigger an alert, manually by phone call during the pilot phase or automatically through an electronic alert in the main study. The alert will recommend an earlier control visit if the patient’s conditions indicate a possible REC/SPM, with the center scheduling an “open urgent appointment” within 2 weeks.

**Table 2:**
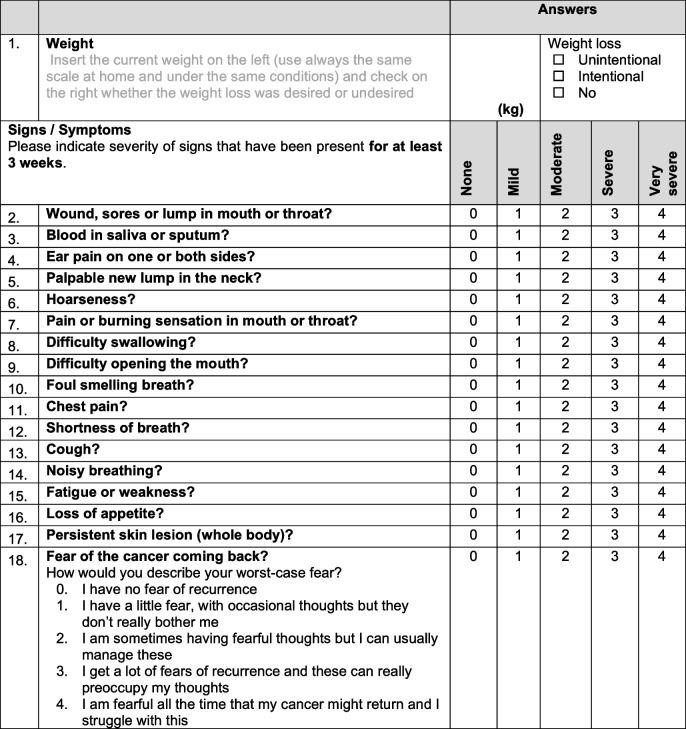
Patient reported outcome questionnaire (symptom tracker)

Patients in the control arm will have scheduled outpatient visits with clinical examination every 3 months (years 1–3) and every 6 months (years 4–5). In addition, patients will undergo scheduled imaging 6 and 18 months after enrollment. Smokers will have additional chest CT-scans 30, 42, and 60 months after enrollment. In the conventional arm, participants will also complete the PROs questionnaire monthly and during every visit, but neither participant calls will be made nor alerts will be sent.

#### Detailed study procedure

The timing of FU visits and the assessments for participants in both arms (conventional FU and individualized deintensified FU) are outlined in Table 3.

**Table 3:**
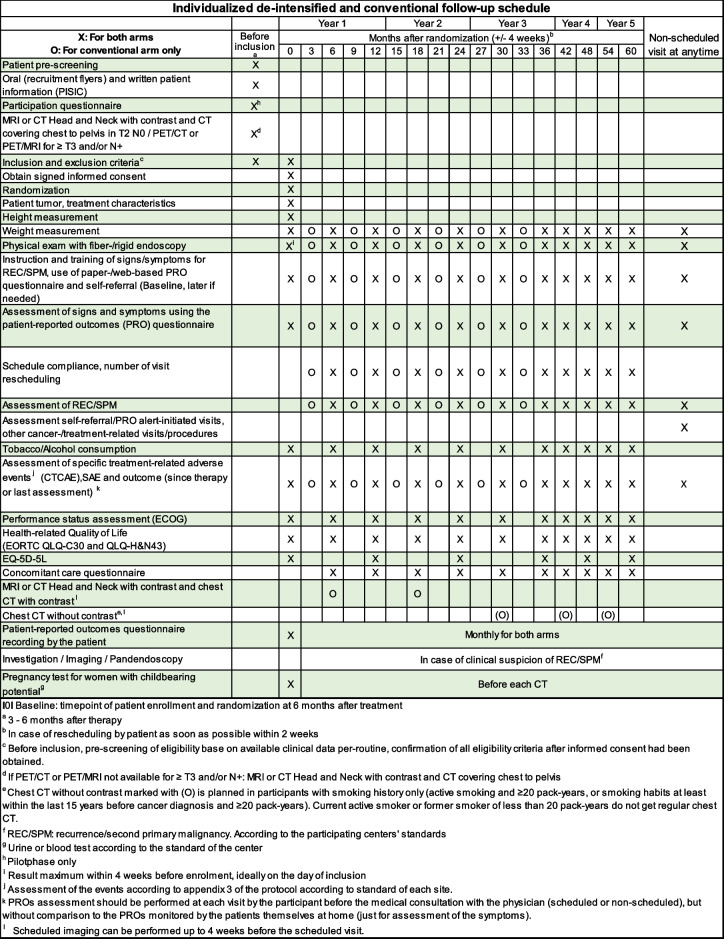
Study assessment schedule

Participants will undergo comprehensive imaging, including head and neck MRI or CT scans and contrast-enhanced trunk CT scans, at 3 to 6 months, and at 6 and 18 months post-randomization, to confirm remission and detect secondary primary malignancies (SPM). Annual low-dose chest CT scans without contrast will be performed on current or recent heavy smokers from 18 months to 5 years, per National Comprehensive Cancer Network (NCCN) Guidelines [13, 24]. Adverse events will be monitored using Common Terminology Criteria for Adverse Events (CTCAE) v.5.0. Medical interactions, including unscheduled visits and supportive treatments, will be recorded via a Concomitant Care Questionnaire. Compliance with FU visits and symptom monitoring using paper or electronic PRO questionnaires will be tracked. Health care utilization costs, including FU visits, imaging, and personnel expenses, will be documented.

#### Fear of REC assessment

Fear of REC is assessed monthly using a single-item screening question developed by Rogers et al. (2015) [49], which rates fear on a scale from 0 to 4 [50]. The English version was translated and back translated to German following European Organisation for Research and Treatment of Cancer (EORTC) standards achieving consensus on the final version.

#### Diagnostic work-up for PRO-triggered visits

For visits triggered by PRO alerts or self-referral, diagnostic work-up follows center standards to assess REC/SPM and/or adverse events. These visits are documented as concomitant care events.

#### Study procedures beyond scheduled FU visits

##### Pilot phase

Participants will fill out a paper PRO form monthly. The study team will call monthly to collect PRO data, offering up to three reminders and further training, if needed. They will advise earlier visits for suspected REC/SPM with the center arranging these within 2 weeks.

##### Main study (ePRO)

Participants will use a monthly ePRO with alerts triggering faster visits for REC/SPM within 2 weeks for the experimental group. The control group will not receive such alerts.

#### Self-referral visits

Additionally, any participant can initiate non-scheduled visits, with centers obliged to accommodate within 2 weeks.

#### Procedure for suspected REC/SPM

When REC/SPM is suspected, centers will follow their protocols for diagnostic work-up, recording all steps in the eCRF.

Post-confirmation a tumor board will outline the treatment approach. FU deviating from the study will conform to center standards, continuing for 5 years or until death. Data on REC/SPM diagnosis, treatment, and participant health status, including last weight, tobacco/alcohol use and symptom scores, will be collected and tracked.

#### Procedure in case of death

The eCRF will record the date and cause of death, distinguishing if it is related to the primary cancer, SPM, treatment, or other causes.

#### Retention and patient involvement strategies

To promote adherence, participants will get a color-coded alarm flyer outlining FU plans and space for future appointments. The flyer serves as a reminder for post-treatment progress and alerts patients to REC/SPM signs, emphasizing the importance of FU compliance and symptom-driven self-referral with quick contact details provided.

#### Training for early detection

Education on recognizing REC/SPM and adverse effects is given to all. The study uses a monthly ePRO (initially paper-based) to monitor symptoms with automated alerts for the experimental group to schedule earlier visits for REC/SPM symptoms and ensure questionnaire completion and adherence.

#### Non-attendance and recall procedures

For unattended appointments, participants will be contacted up to three times before marking “discontinuation of FU scheme” in the eCRF, unless primary endpoint data are obtainable from the family physician. Unconfirmed survival at 5 years will be “Lost to FU”.

#### Discontinuation of FU

##### Discontinuation criteria

Participants can leave the FU study for REC/SPM diagnosis, serious adverse event (SAE), significant medical condition, pregnancy, non-compliance after three recalls or by own request.

##### Status post-discontinuation

Participants leaving the FU scheme, who do not withdraw consent remain in the trial and should follow the centers’ standard FU protocol.

### Statistical analysis

After a 1-year recruitment period, the primary objective assessment will proceed based on participant accrual. A patient motivation questionnaire will be used to optimize enrollment and refine the study design. Adherence will be monitored until the final participant completes the 12-month FU in the second year. Data from the internal pilot phase will inform potential adjustments to the main trial but will not be analyzed separately unless the trial does not advance.

The internal pilot phase sample size is informed by feasibility objectives, including recruitment rates, participant retention, and protocol adherence, which are critical for assessing the study’s viability. Drawing on internal data from the Department of Oto-Rhino-Laryngology, Head and Neck Surgery at Inselspital, Bern University Hospital, the anticipated participant pool aligns with real-world metrics, ensuring a practical recruitment target for the main trial. This sample size allows for robust evaluation of recruitment and adherence metrics without compromising statistical power for the primary endpoint, given that the main trial’s effect measure—5-year restricted mean survival time—has been calibrated based on historical data approximated by a Weibull distribution (shape parameter *a* = 1.18, scale parameter *b* = 10.25), supporting a 5-year RMST of 4.65 years.

While the pilot’s sample size primarily assesses feasibility metrics, it does not account for the interim analysis, designed as non-binding and thus not impacting type I or II error rates. However, feasibility insights from the pilot phase will guide potential adjustments, ensuring that the full study, expected to enroll 550 participants, meets its recruitment and data quality standards.

Data from the internal pilot phase will be analyzed descriptively to assess feasibility metrics, such as recruitment rates and adherence. The participation questionnaire data will undergo descriptive and qualitative analysis to inform potential adjustments to the main trial, particularly in enrollment strategy and patient information. However, endpoint data collected during the pilot will only contribute to the main trial analysis if the study proceeds.

In the main study, analysis sets are defined in line with estimands, including intention-to-treat and two per-protocol sets. The intention-to-treat set will consist of all randomized participants, regardless of protocol violations, while the per-protocol sets will define adherence differently: (a) “while-on-treatment” excludes non-adherence due to medical reasons, and (b) “hypothetical” includes any non-adherence. Adherence will be calculated based on scheduled visits completed within a ± 4-week window, with classification occurring only before REC/SPM occurrence. The main study’s primary endpoint, 5-year restricted mean survival time (RMST), and secondary endpoints will be analyzed using mixed-effects and negative binomial models, with adjustments for competing events [51–55]. A cost and cost-utility analysis will be performed if the trial shows promising non-inferiority results. This analysis will compare the incremental cost-utility ratio of the deintensified versus standard FU, using quality-adjusted life years (QALYs) and standardized healthcare costs.

For detailed procedures and analysis methods, refer to the full protocol submitted as supplementary material (see Additional file 1: DeintensiF-Full Protocol).

## Discussion

Routine FU protocols for HNSCC patients typically include ENT exams and imaging. While various international guidelines support these standard FU schedules, retrospective studies offer mixed evidence on their impact, suggesting that standard FU does not consistently improve oncological outcomes over symptom-driven self-referral [28–34]. While two studies suggest a survival benefit of routine FU over self-reported symptoms [26, 27]. Evidence generally shows, that patients detect REC sooner than physical exams. Silent REC remains rare with low detection rates in routine visits [30, 35–38].

HPV-driven HNSCC, expected to become more prevalent by 2030, may further reshape FU protocols, emphasizing the need for validated methods that respond to both REC detection and changing etiological patterns [42, 56–60]. Current evidence to support the oncological benefits of regular FU remains sparse, raising questions about the value of routine visits compared to a symptom-based approach that has shown high sensitivity and negative predictive value [61–65].

Imaging remains a crucial yet controversial component of FU for HNSCC, largely due to its implications for QoL and cost. While imaging can aid in detecting locoregional REC early, its utility in asymptomatic cases is limited by high rates of false positives, which often lead to unnecessary interventions and added anxiety [66–68]. PET/CT scans following CXRT have shown greater sensitivity for detecting REC but vary in clinical impact depending on the timing of detection and disease progression [69–76]. Trials like PET-NECK suggest that PET/CT-guided surveillance may achieve similar survival rates to standard FU protocols, with fewer surgeries and improved cost-effectiveness [76–86]. Despite these benefits, imaging beyond 6 months post-treatment for asymptomatic patients in complete remission remains debatable, as similar survival outcomes have been observed regardless of the detection method used [87].

For many patients, the FU process poses logistical, psychological, and financial burdens, often diminishing QoL. Studies indicate that while some patients feel reassured by routine FU, others experience heightened anxiety and express a preference for a less intense FU schedule [88–93]. Balancing the need for patient-centered care with evidence-based FU strategies could mitigate unnecessary radiation exposure, reduce stress, and lower costs associated with high false-positive rates, ultimately supporting a de-escalated FU approach [49, 94]. Additionally, FU protocols for HNSCC span several years and impose a significant financial burden. Research highlights the substantial QoL reductions experienced by HNC patients up to 24 months post-treatment, with a clear need for long-term care strategies that address persistent impairments [95]. While intensive FU has not demonstrated cost-effectiveness in other cancers, HNC-specific economic analyses remain limited. Addressing these economic and QoL impacts through a prospective, multicenter study could provide valuable insights into an optimal balance for FU strategies [96].

Ongoing research, including trials like HETeCo and SURVEILL’ORL, is currently exploring the cost-effectiveness and survival impact of PET/CT, while PETNECK 2 evaluates patient-initiated FU for low-risk HNSCC patients [NCT03519048, NCT02262221, NIHR200861]. These studies highlight the potential of targeted FU for selected HNSCC patients, particularly for those with HPV-positive cancer, offering valuable data that could be used as a reference point for broader studies, such as the one proposed here.

The presented DeintensiF trial aims to generate high-quality data to benefit patients and healthcare providers by establishing evidence-based follow-up (FU) guidelines for head and neck cancer. It will compare medical and patient-initiated FU procedures, assessing their impact on outcomes. Additionally, data on quality of life, anxiety, depression, and fear of recurrence will be collected to understand the psychological effects. Efforts to improve cost-efficiency will also be explored, potentially reshaping global clinical practice for HNC FU.

With REC primarily driven by symptoms, electronic patient-reported outcomes (ePROs), present a promising method for real-time symptom tracking, showing survival benefits in other cancer types through improved early detection and outcomes [36, 44]. However, the effectiveness of routine exams for detecting REC or SPM is uncertain due to high false-negative rates, and physical exams alone often fail to capture early signs of REC. With no proven survival benefit from routine over symptom-driven FU in HNC, the DeintensiF main trial will further investigate whether a deintensified FU approach can safely match the mortality outcomes of standard FU while preserving patients’ QoL. Symptom-based consultations may provide outcomes similar to those of standard FU in detecting REC or SPM, thereby presenting a patient-adapted, cost-efficient alternative.

Ethical considerations underscore the need for a randomized trial to address the current lack of prospective data on HNC follow-up (FU). The DeIntensiF trial will compare FU schedules with and without imaging to evaluate their effects on survival, oncological outcomes, and recurrence detection. It will also assess the influence of patient-reported symptoms, demographics, tumor characteristics, QoL, and costs across two FU schemes. Findings aim to support evidence-based, personalized surveillance tailored to tumor specifics and patient needs. The pilot phase will explore feasibility in randomizing patients with complete remission to different FU schemes. Participation is voluntary, with the right to withdraw anytime; no vulnerable participants will be included. To minimize the dropouts in the main study on the grounds of electronic illiteracy, patients are given the possibility to opt for an offline paper version of the ePROs questionnaire. Those patients will be called by the responsible site mimicking the web-based tool. Health literacy (HLS-EU-Q16) [97] and e-health literacy (eHEALS) [98] will be assessed during randomization to control for effects in compliance and applying (subgroup analysis). In addition to assessing health and e-health literacy during randomization, these metrics will be incorporated into our statistical models to adjust for potential variations in compliance and outcomes between patients using paper-based versus electronic PROs, ensuring that mode-related biases are minimized.

Frequent physical and radiological exams are a major logistical, psychological and financial burden for vulnerable and older cancer patients. We therefore expect de-intensification of FU to be especially welcomed by this group of patients. The trial offers efficient FU protocols that reduce unnecessary visits and risks, addressing QoL aspects often overlooked by current guidelines. For medical staff, it will generate evidence to optimize post-treatment care and inform international standards. The healthcare system may benefit through clearer value assessments of FU procedures and cost-efficient de-intensified practices if shown non-inferior, reshaping HNC FU worldwide.

Data will be securely coded with access limited to authorized personnel. The pilot trial is registered on a public registry (https://www.clinicaltrials.gov) The main study will also be registered on the above mentioned registry and on a public website providing details and regular updates. An abbreviated protocol of the main study will be published in an open access journal. Interim safety analyses, if deemed impactful on the trial or FU guidelines, may be communicated early, unless halted for safety reasons, in which case the interim forms the final analysis. The main findings, based on the final analysis, will be published in a peer-reviewed open-access journal within a year of the database lock, with further analyses following the main publication. Investigators involved are members of relevant medical societies and guideline committees and will work to reflect results in national and international guidelines. Results from the pilot study and the main trial will be presented to both patients and professionals through diverse formats, including congresses and media, to ensure practical application and influence on global FU practices.

## Supplementary Information

Additional file 1. DeintensiF-Full Protocol.

## Data Availability

An anonymized trial dataset will available after the completion of the study. This will be shared on request, at the discretion of the authors. The study is currently ongoing.

## Abbreviations

CXRT: Chemoradiotherapy
CT: Computed tomography
CTCAE: Common Terminology Criteria for Adverse Events
DMC: Data Monitoring Committee
DFS: Disease-free survival
ENT: Ear, nose, and throat
eCRF: Electronic Case Report Form
ePRO: Electronic Patient-Reported Outcomes
FU: Follow-up
HNC: Head and neck cancer
HNSCC: Head and neck squamous cell carcinoma
HPV: Human papillomavirus
MRI: Magnetic resonance imaging
NCCN: National Comprehensive Cancer Network
OS: Overall survival
PET: Positron emission tomography
PRO: Patient-reported outcomes
QALY: Quality-adjusted life year
QoL: Quality of life
REC: Recurrence
RMST: Restricted mean survival time
secTrial®: A secure online system for patient registration and randomization
SPM: Second primary malignancies
TNM: Tumor, node, metastasis
UICC: Union for International Cancer Control
