# Supplementary material for: DeintensiF: Standard versus individualized deintensified follow-up after curative treatment in head and neck cancer: protocol of a randomized pilot study
Source: Pilot Feasibility Stud. 2025 May 16;11:69. doi: 10.1186/s40814-025-01651-3 (PMC12083109; doi:10.1186/s40814-025-01651-3)
Supplement: Supplementary file 1 — Additional file 1. DeintensiF-Full Protocol. [file 40814_2025_1651_MOESM1_ESM.pdf]

DeintensiF: Multicenter randomized trial comparing an individualized de-intensified and conventional follow-up strategy after curative treatment in head and neck cancer

Study Type:	Other Clinical Trial according to ClinO, Chapter 4
Risk Categorisation:	Risk category A according to ClinO, Art. 61
Study Registration:	ClinicalTrials.gov (NCT05388136)<br>Swiss National Clinical Trial Portal (SNCTP000005198)
Study ID:	1685 DeintensiF
Sponsor:	Inselspital, Bern University Hospital,<br>University of Bern<br>3010 Bern, Switzerland<br><br>Represented by Coordinating-Investigator:<br>Prof. Dr. med. Roland Giger<br>Department of ENT, Head and Neck Surgery<br>Inselspital, Bern University Hospital<br>Bern, Switzerland<br>Tel: +41 31 632 29 31<br>Fax: +41 31 632 88 09<br>Email: roland.giger@insel.ch
Investigated Intervention:	De-intensified follow-up strategy after curative treatment in head and neck cancer
Version and Date:	Version 3.1 (dated 13/12/2022)

CONFIDENTIALITY STATEMENT

The information contained in this document is confidential and the property of the sponsor. The information may not - in full or in part - be transmitted, reproduced, published, or disclosed to others than the applicable Competent Ethics Committee(s) and Regulatory Authority(ies) without prior written authorisation from the sponsor except to the extent necessary to obtain informed consent from those who will participate in the study.

PROTOCOL SIGNATURE FORM

Study Title	Multicenter randomized trial comparing an individualized de-intensified and conventional follow-up strategy after curative treatment in head and neck cancer
Study ID	1685 DeintensiF

The Coordinating-Investigator has approved this protocol version and confirm hereby to conduct the study according to the protocol, current version of the World Medical Association Declaration of Helsinki, and ICH-GCP guidelines as well as the local legally applicable requirements.

Coordinating-Investigator:

Name: Prof. Dr. med. Roland Giger

Date: 15.12.2022 Signature: R Giger

Local Principal Investigator at study site:
(See list of involved institutions in **Appendix 4**)

I have read and understood this trial protocol and agree to conduct the trial as set out in this study protocol, the current version of the World Medical Association Declaration of Helsinki, ICH-GCP guidelines and the local legally applicable requirements.

Site (name & address):

Principal Investigator (printed name):

Date: ____________________________ Signature: ______________________________

Table of Contents

GLOSSARY OF ABBREVIATIONS 7
1 STUDY SYNOPSIS 9
2 BACKGROUND AND RATIONALE 15
2.1 Disease background 15
2.2 Background in follow-up for head and neck cancer patients 16
2.3 Rationales for the trial 18
2.4 Routine systematic follow-up 18
2.5 Imaging 21
2.6 The patient’s perspective 22
2.7 Costs 22
2.8 Other existing trials 23
3 STUDY OBJECTIVES AND DESIGN 24
3.1 Hypothesis and primary objective 24
3.1.1 Objectives of the pilot phase 24
3.1.2 Objectives of the main study 24
3.2 Primary and secondary endpoints 25
3.2.1 Main study 25
3.2.2 Pilot study 26
3.3 Estimands 27
3.3.1 Anticipated intercurrent events 27
3.3.2 Primary estimand 27
3.3.3 Secondary estimands 28
3.4 Study design 29
3.4.1 Internal pilot phase and progression criteria for moving to the main phase of the trial 30
3.4.2 Study duration 30
3.5 Study intervention 30
3.5.1 Experimental arm - Individualized de-intensified FU 30
3.5.2 Control arm - Conventional (standard) FU 31
4 STUDY POPULATION AND STUDY PROCEDURES 32
4.1 Inclusion and exclusion criteria, justification of study population 32
4.1.1 Inclusion criteria 32
4.1.2 Exclusion criteria 32
4.2 Recruitment, screening and informed consent procedure 33
4.2.1 Recruitment 33
4.2.2 Pre-screening 33
4.2.3 Informed consent procedure 33
4.3 Study procedures 34
4.3.1 Patient registration and randomization procedure 34
4.3.2 Electronic case report forms completion 35
4.3.3 Detailed study procedure at each visit 35
4.3.4 Other evaluation and supportive care 41

4.3.5 Study procedure outside of the scheduled in-person FU visits 42
4.4 Procedure in suspicion of recurrence/second primary malignancy 42
4.4.1 Assessments in case of recurrence/second primary malignancy 42
4.4.2 Procedure in case of death 43
4.5 Follow-up after 5 years 43
4.6 Strategies for increased retention and patient involvement 44
4.6.1 Engaging and sensibilize the participant 44
4.6.2 Recall 44
4.7 Discontinuation 45
4.8 Withdrawal 46
4.9 End of study 46
5 STATISTICS AND METHODOLOGY 47
5.1 Statistical analysis plan and sample size calculation 47
5.1.1 Justification of the sample size 47
5.1.2 Statistical analysis of the internal pilot phase 48
5.1.3 Statistical analysis of the main trial 48
5.2 Handling of missing data and drop-outs 51
5.2.1 Missing data 51
5.2.2 Drop-outs 51
6 REGULATORY ASPECTS AND SAFETY 52
6.1 Local regulations / Declaration of Helsinki 52
6.2 (Serious) adverse events and notification of safety and protective measures 52
6.2.1 Anticipated (S)AE in this study 53
6.2.2 Reporting of (serious) adverse events 54
6.3 (Periodic) safety reporting 55
6.4 Pregnancy 55
6.5 Amendments 55
6.6 Notification and reporting upon completion, discontinuation or interruption of the study 55
6.6.1 Overall study discontinuation 55
6.6.2 Site or investigator discontinuation 56
6.7 Insurance 56
7 FURTHER ASPECTS 57
7.1 Overall ethical considerations 57
7.2 Risk-benefit assessment 58
8 QUALITY CONTROL AND DATA PROTECTION 58
8.1 Quality measures 58
8.2 Data recording and source data 59
8.2.1 eCRF 59
8.2.2 Paper and electronic patient reported outcome questionnaire 59
8.2.3 Source data 60
8.3 Confidentiality and coding 60
8.4 Retention and destruction of study data and biological material 61

9 MONITORING AND STUDY REGISTRATION 62
9.1 Monitoring 62
9.2 Study registration 62
10 FUNDING / PUBLICATION / DECLARATION OF INTEREST 63
10.1 Funding 63
10.2 Publication 63
10.3 Declaration of interest 63
11 REFERENCES 64
12 CHANGE HISTORY 68
13 APPENDIXES 69

GLOSSARY OF ABBREVIATIONS

ADL	*Activities of Daily Living*
AE	*Adverse Event*
ASR/DSUR	*Annual Safety Repot / Development Safety Report*
ASHNS	*American Society of Head and Neck Surgeons*
BAHNO	*British Association of Head and Neck Oncologists*
BASEC	*Business Administration System for Ethical Committees*
CDMS	*Clinical Data Management System*
ClinO	*Ordinance on Clinical Trials in Human Research (in German: KlinV, in French: OClin, in Italian: OSRUm)*
CTCAE	*Common Terminology Criteria for Adverse Events*
CT scan	*Computed Tomography scan*
CTU	*Clinical Trial Unit*
DCHNO	*Dutch Cooperative Head and Neck Oncology Group*
DFS	*Disease-Free Survival*
DMC	*Data Monitoring Committee*
DNA	*Deoxyribonucleic Acid*
EC	*Ethics Committee*
eCRF	*Case Report Form*
ECOG	*Eastern Cooperative Oncology Group*
ENT	*Ear Nose Throat*
EORTC	*European Organisation for Research and Treatment of Cancer*
EQ-5D-(5L)	*EuroQol Five Dimensions Questionnaire*
FADP	*Federal Act on Data Protection (in German: DSG, in French: LPD, in Italian: LPD)*
FOPH	*Federal Office of Public Health*
FOS	*French ORL Society*
FU	*Follow-up*
GCP	*Good Clinical Practice*
H&N	*Head and Neck*
HNC	*Head and Neck Cancer*
HNSCC	*Head and Neck Squamous Cell Carcinoma*
HPV	*Human Papillomavirus*
HRA	*Human Research Act (in German: HFG, in French: LRH, in Italian: LRUm)*
IC	*Inclusion Criteria*
ICH	*International Conference on Harmonisation*
Mets	*Metastasis*
mths	*Months*
MRI	*Magnetic Resonance Imaging*
NCCN	*National Comprehensive Cancer Network*
OS	*Overall Survival*
PET/CT	*^{18}Fluorodeoxyglucose Positron Emission Tomography/Computer Tomography*

PI	*Principal Investigator*
PISIC	*Patient Information Sheet and Informed Consent*
(p/e)PROs	*(paper/electronic) Patient-Reported Outcomes*
PGfAR	*Program Grants for Applied Research*
QALYs	Quality-Adjusted Life Years
QLQ-30	*Core Quality of life of Cancer Patients*
QLQ-H&N43	*Quality of Life Questionnaire-Head & Neck 43*
QoL	*Quality of Life*
LRR	*Loco-Regional Relapse*
REC	*Recurrence*
RMST	*Restricted Mean Survival Time*
RT	*Radiotherapy*
RTCX	*Radiation Therapy with Chemotherapy*
rTNM	*Recurrent TNM Classification of Malignant Tumors*
rStaging	*Recurrent Staging*
SAE	*Serious Adverse Event*
SEER	*Surveillance, Epidemiology, and End Results*
SGORL	*Schweizerische Gesellschaft für Oto-Rhino-Laryngologie, Hals- und Gesichtschirurgie*
SNCPT	*Swiss National Clinical Trial Portal*
SNSF	*Swiss National Science Foundation*
SHNS	*Society of Head and Neck Surgeons*
SPM	*Second Primary Malignancy*
SSORL	*Swiss Society of Oto-Rhino-Laryngology, Head and Neck Surgery*
TNM	*TNM Classification of Malignant Tumors*
Tx	*Treatment*
UICC	*Union for International Cancer Control*
WHO	*World Health Organization*

1 STUDY SYNOPSIS

Sponsor / Coordinating-Investigator	Inselspital Bern (Sponsor) represented by<br><br>Prof. Dr. med. Roland Giger (Coordinating Investigator)<br><br>Department of ENT, Head and Neck Surgery, Inselspital<br><br>Bern University Hospital, University of Bern, Bern, Switzerland<br><br>Tel: +41 31 632 29 31<br><br>Fax: +41 31 632 88 09<br><br>Email: roland.giger@insel.ch
Study Title	Multicenter randomized trial comparing an individualized de-intensified and conventional follow-up strategy after curative treatment in head and neck cancer
Short Title / Study ID	DeintensiF
Protocol Version and Date	Version 3.1 (dated 13/12/2022)
Study Registration	ClinicalTrials.gov (NCT05388136)<br><br>Swiss National Clinic al Trial Portal (SNCTP000005198)
Study Category and Rationale	Other clinical trials, category A, according to the Ordinance on Clinical Trials in Human Research (ClinO), Chapter 4, Art. 60: Clinical trials with interventions recognized as standard in guidelines prepared in accordance with internationally accepted quality criteria.
Background and Rationale	**Background:**<br><br>There are estimated 900'000 head and neck cancer (HNC) patients with over 400'000 deaths per year worldwide (Europe: 150'000 new cases per year). Approximately 70% present with locoregionally advanced disease. The curative rate for early-stage disease is high (80-90%). On the contrary, despite advances in surgery, radiotherapy and the development of new systemic therapies, about 50-60% of advanced tumors will recur locoregionally after front line treatment, and 20-30% will have distant metastases. Additionally, about 2-4% of patients will develop a second primary malignancy (SPM) with each passing year.<br><br>Follow-up (FU) is important to detect disease recurrence (REC) and SPM at an early stage to put effective therapy in place, manage treatment-related sequelae, and provide physical rehabilitation and psychosocial support to patients and their families.<br><br>Due to the absence of prospective studies and evidence-based data, there is no clear consensus within national and international guidelines, on how to perform FU. There are only retrospective studies addressing this topic, showing no difference in overall survival (OS) between patients with detected REC in routine FU and symptom-driven self-referral visits. The use of imaging to detect REC and SPM is also a subject of debate. Moreover, many of the published studies did not include the logistical, psychological and financial consequences for the patients and their relatives and the increasingly relevant cost evaluations in todays' financially-focused healthcare systems.<br><br>**Rationale:**<br><br>Head and neck cancer patients represent a heterogeneous group, and there is currently an ongoing debate to determine the optimal frequency, content and duration of FU care. Today, there are no prospective studies with evidence-based data, which leads to the following two

	major problems: 1) the lack of consolidated data about FU (procedures and timing) among different institutional, national and international guidelines; and 2) the need for a tailored approach that considers different prognostic factors and patients' performance status/age. One crucial unsolved question is whether early detection of REC/SPM in asymptomatic patients has an impact on survival and quality of life (QoL).
Risk / Benefit Assessment	**Individualized de-intensified FU entailing less frequent clinical exams and no routine imaging:**<br><br>Benefits: increased individualized involvement during the FU, monthly surveillance of the patient-reported outcome questionnaire with triggers of alerts for a control earlier than scheduled visits depending on the ratings of signs and symptoms indicating possible REC/SPM followed by procedures to exclude REC/SPM. Possible early detection of REC/SPM caused by the more intensive surveillance of pathological signs and symptoms. Less frequent FU visits, no additional x-ray harm due to the absence of systematic radiological imaging, decreased stress due to decreased frequency of FU visits, decreased individual costs for transport/insurance.<br><br>Risks: possible late detection of REC/SPM due to less frequent FU visits or the absence of systematically planned imaging, increased stress caused by the absence of systematic radiological imaging confirming objectively disease-freeness.<br><br>**Conventional (standard) FU entailing frequent clinical exams and routine imaging:**<br><br>Benefits: more frequent FU visits, systematic radiological imaging confirming objectively and systematically disease-freeness (after 12 and 24 months and then yearly in heavy smokers), possible early detection of REC/SPM caused by more frequent FU visits and systematic imaging.<br><br>Risks: possible late detection of REC/SPM due to the absence of increased individualized involvement and no systematic surveillance of the patient-reported outcome questionnaire and no trigger of alerts for a control earlier than scheduled visits. Harm due to systematic radiological imaging, increased stress due to the frequent FU visits, increased individual costs for transport/insurance, radiological detection of lesions (also of benign nature) leading to further possible harmful investigations/interventions to exclude REC/SPM.<br><br>Both FU modalities under investigation are associated with distinct benefits and minimal, but potential risks.
Objective(s)	**Pilot phase**<br><br>Primary objective<br><br>The primary objective of the pilot phase of this trial is to evaluate_the feasibility of recruiting and randomizing patients with complete remission 6 months after treatment of head and neck squamous cell carcinoma (HNSCC) to a de-intensified and conventional (standard) FU in regard to recruitment rate.<br><br>Secondary objectives<br><br>The secondary objective relates to investigating adherence to the two different FU strategies. Good compliance is defined as the realization of the planned/organized visits and exams at the planned time +/- 4 weeks as well as regularly recording of signs and symptoms in the patient-reported outcomes (PROs) questionnaire.<br><br>**Main study**<br><br>Primary objective

<table>
<tr><td></td><td>The primary aim of the study is to determine whether an individualized de-intensified approach to surveil patients is non-inferior to a conventional FU in HNC with regard to 5-year restricted mean survival time (RMST).

<u>Secondary objectives</u>

Comparison of the impact of a de-intensified to a conventional (standard) FU from the date of randomization up to 1 and 5 years, or until the date of multidisciplinary tumor board decision for the treatment strategy concerning the first REC/SPM after the primary treatment or until death in regard to:

• HNC-specific survival, any cancer-specific survival
• Detection rate of REC/SPM (as “time to endpoint”)
• Incidence, site and stage of REC
• Incidence, histopathology, site (head and neck, lung, etc.) and stage of SPM
• Rate of REC/SPM amenable to curatively-intended therapy
• Type and the actual number of FU visits
• Assessment of patients’ compliance based on the rate of missed scheduled appointments → accepted or refused/ignored recall appointments within 4 weeks
• Assessment of patients’ additional visits between the scheduled visits
• Fear of REC
• Health-related QoL
• Healthcare costs

<u>Descriptive objectives</u>

• Late toxicities (types and grading) and outcome
• Characteristics of REC/SPM (diagnostic modalities that lead to detection of REC/SPM)
• Adherence to signs/symptom monitoring and triggers that lead to recommendation for a control earlier than scheduled visit</td></tr>
<tr><td>Endpoint(s)</td><td>Main study

<u>Primary endpoint</u>
Death from any cause

<u>Secondary endpoints (all at 5 years)</u>
• Death from HNC (time to HNC-specific death)

• Death from any cancer (time to cancer-specific death)

• First biopsy-proven REC or SPM (time to REC/SPM) based on participating centers’ assessment according to the UICC/TNM 8th ed.

• General health-related QoL as assessed every 6 months over 5 years by means of the European Organisation for Research and Treatment of Cancer, Core Quality of Life of Cancer Patients (EORTC QLQ-C30)

• Head and neck cancer-specific health-related QoL as assessed every 6 months over 5 years by means of the European Organisation for Research and Treatment of Cancer, Head and Neck Cancer Module (EORTC QLQ-HN43)

• Compliance with scheduled FU assessments

• Number of regularly scheduled in-person visits

• Number of in-person visits triggered by the recommendation of the PRO</td></tr>
</table>

	• Number of self-referral in-person visits<br>• Number of any in-person visits<br>• Fear of REC<br>• HNC-specific healthcare utilization<br><br>Description on:<br>• Type and grading of specific treatment-related adverse events and outcome<br>• Characteristics of REC/SPM (diagnostic modalities that lead to detection of REC/SPM, incidence, site, stage, and whether it is amenable to curatively-intended salvage therapy)<br>• Adherence to electronical signs/symptom monitoring and visits that are triggered by the PRO<br>• All assessments for secondary endpoints will end at 5 years FU, the date of multidisciplinary tumor board decision for the treatment strategy concerning the first REC/SPM after the primary treatment or at death.<br><br>Pilot phase<br><br>Besides the endpoints of the main trial, we will collect additional data to achieve the objectives of the pilot phase. Some of these are not patient-level assessments in the narrow sense but are still provided.<br>• Eligibility rate defined as the proportion of all eligible patients out of all screened patients<br>• Consent rate defined as the number of consenting patients out of all eligible patients<br>• Overall accrual per month<br>• Motivation for participation or not consenting based on a participation questionnaire
Study Design	Multicenter, randomized study evaluating a de-intensified and conventional (standard) FU after curative treatment in HNSCC patients.
Statistical Considerations	Sample size calculation<br><br>Sample size calculation is based on the primary endpoint of death from any cause, the effect measure of 5-year RMST, and the dual-criterion design. The sample size calculation does not take into account the interim analysis (at a minimal FU of 20 months) as this is designed as non-binding and therefore not formally influencing the type I (or II) error of the trial. Sample size calculation is based on simulations (10,000 replications for each scenario) where we also investigated the operating characteristics of the trial.<br><br>We used internal data of the Department of Oto-Rhino-Laryngology, Head and Neck Surgery, Inselspital, Bern University Hospital to inform overall survival in the control group. This is high-quality data prospectively collected from 2016-2019. Only eligible patients to the proposed trial are considered (N=179 of which 35 died) with a median follow-up of 2.9 years. The data could be well approximated by a Weibull distribution with shape parameter a=1.18 and scale parameter b=10.25. This corresponds to a 5-year RMST of 4.65 years.<br><br>Using the assumptions of 3 years of recruitment, 5-year FU per patient and a power >90%, we calculated a required sample size of 525 patients overall. To account for uncertainties in the

	assumptions, we fixed the final sample size at 550 patients overall.<br><br>Data analysis<br><br>Data for endpoints of the internal pilot phase will be analyzed descriptively. Endpoint data for the main trial that is collected during the internal pilot phase will only be used for the main trial and not analyzed at the end of the internal pilot phase unless the trial will not progress to the main trial.<br><br>In line with the estimands defined, we define three different analysis sets for this trial of a sustained intervention: 1) treatment policy or intention-to-treat; 2) while-on-treatment (per-protocol 1); 3) hypothetical (per-protocol 2). As primary effect measure, the difference in the 5-year RMST will be derived from a flexible parametric survival model adjusted for stratification factors used at randomization. It will be accompanied by a one-sided 97.5% confidence interval to test for non-inferiority of the experimental FU scheme.<br><br>Analyses of other endpoints will be done using the intention-to-treat analysis set only.
Inclusion- / Exclusion Criteria	**Inclusion criteria**<br><br>1. Histopathologically proven invasive HNSCC of the oral cavity (except lip), oropharynx, hypopharynx or larynx<br>2. ≥18 years of age<br>3. In non-surgically treated HNSCC: clinical/radiological stage II-IV (excluding M1) according to the UICC / TNM 8th ed. In surgically treated HNSCC: pathological stage II-IV (excluding M1) according to the UICC / TNM 8th ed.<br>4. Treatment with curative intent, regardless of treatment modality (mono- or multimodal), and FU planned at the participating study center.<br><br>Remark: Patients with one synchronous HNSCC of the oral cavity, oropharynx, hypopharynx and larynx, all treated with curative intent and all in complete remission are eligible. Synchronous tumor must have a less advanced stage than the index tumor used for stratification or in case of equal stage, the synchronous tumor must be the tumor with the better prognostic. (Rules: Better to worse prognostic: Larynx > Oropharynx > Oral cavity > Hypopharynx.) The modality of the treatment must be the same as for the index tumor or less intense.<br><br>5. Radiological confirmation of complete remission of disease and no SPM from the 3rd to 6th month after treatment for all stages (minimal demanded imaging: head and neck (H&N) MRI or H&N CT scan and CT scan covering chest to pelvis (with contrast if not contraindicated); or preferable whole-body ^{18}FDG-PET/CT or PET/MRI for patients with ≥T3 and/or N+).<br><br>Note: Patients with positive or equivocal imaging/clinical findings are allowed if the tumor is ruled out by multidisciplinary tumor board decision (e.g. as a consequence of biopsy and/or multiple imaging).<br><br>6. Clinical confirmation of complete remission of disease through H&N examination including endoscopy of the pharynx and larynx at the time of enrolment, that is 6 months (+/- 4 weeks) after the last HNSCC treatment<br>7. Agreement for long term FU (5 years) and all visits are to be performed at the participating center<br>8. Written informed consent, signed by the patient and the investigator<br><br>**Exclusion criteria**<br><br>1. Initial clinical stage I and/or M1 HNSCC (according to the UICC / TNM 8th ed.)<br>2. Nasopharyngeal cancer and carcinoma of unknown primary<br>3. Any other previously treated HNC (including parotid and thyroid gland cancer) except for curatively and adequately treated cutaneous carcinoma in-situ, basal cell carcinoma and locally confined T1 squamous cell carcinoma of the skin without any sign of tumor recurrence at the time of screening

	4. Any other synchronous malignancy except for one curatively and adequately treated HNSCC of the oral cavity, oropharynx, hypopharynx and larynx, basal cell carcinoma, locally confined T1 squamous cell carcinoma of the skin, low-risk prostate cancer, carcinoma in-situ of the skin or uterine cervix without any sign of tumor recurrence at the time of screening.<br>5. Any other metachronous malignancy within the last 5 years except for curatively and adequately treated basal cell carcinoma, locally confined T1 squamous cell carcinoma of the skin, low-risk prostate cancer, carcinoma in-situ of the skin or uterine cervix without any sign of tumor recurrence at the time of screening.<br>6. Participation in another study entailing regular medical exams by ENT specialists or persons involved in the oncological treatment, or regular imaging<br>7. Pregnant or breastfeeding women<br>8. Presence of any conditions that potentially hamper compliance with the study protocol and FU schedule at the participating center
Number of Participants with Rationale	**Pilot phase:**<br><br>The pilot phase will be conducted in three Swiss centers, which will also participate in the main trial. Within 1 year, we expect to include 20 patients (10 in each arm). Since feasibility is a crucial point in this kind of study, we will assess feasibility in terms of recruitment and compliance to FU exams among the first 20 patients and the first year of the trial.<br><br>**Main study:**<br><br>550 participants in total, 275 patients per arm<br><br>Sample size calculation is based on the primary endpoint of death from any cause, the effect measure of 5-year restricted mean survival time, and the dual-criterion design. Using these assumptions, we calculated a required sample size of 525 participants overall. To account for uncertainties in the assumptions, we fixed the final sample size at 550 participants overall.
Study (experimental) Intervention	Individualized de-intensified FU entailing less frequent clinical exams and no routine imaging (11 visits including visit at randomization) without any routinely planned imaging during 5 years of FU.<br><br>Participants receive intensive sensitization/training to recognize signs and symptoms of REC/SPM and treatment-related adverse events (standardized alarm flyers) and the H&N symptoms will be monitored via patient-reported outcomes (PRO) with rating scale.<br><br>In the pilot phase, participants will monitor their symptoms by completing a paper PRO questionnaire monthly and the study team will call and interview for the result. Recommendation for a control earlier than scheduled visit is triggered based on a set of pre-defined conditions indicating possible REC/SPM.<br><br>In the main study, an electronic patient-reported outcome (ePRO) with rating scale is to be completed monthly by the participant. The ePRO result will trigger an automated alert to the participant and to the site in conditions indicating possible REC/SPM. In case of possible REC/SPM, an 'open urgent appointment' will be arranged for the participant by the participating centers' team within 2 weeks.
Control Intervention	Conventional (standard) FU entails frequent clinical exams and routine imaging (17 visits including a visit at randomization, plus 4 or 7 imaging depending on smoking habits of the participant during 5 years of FU.<br><br>Participants receive intensive sensitization/training to recognize signs and symptoms of REC/SPM and treatment-related adverse events (standardized alarm flyers) and the H&N symptoms will be assessed via PROs with rating scale.<br><br>In the pilot phase, participants will assess their symptoms by completing a paper questionnaire monthly.

	In the main study, an ePRO with rating scale is to be completed monthly by the participant, but no alert will be generated and the participating center will not arrange urgent appointment, except in case of self-referral for any reasons.
Study procedures	1) Patient pre-screening base on baseline data per routine to access eligibility criteria<br>2) Oral and written patient information given by investigators<br>3) Obtain written participant informed consent<br>4) Assessment of baseline data before randomization<br>5) Assessment after randomization<br>6) FU schedules and exams/imaging depending on the assigned randomization arm<br>7) Assessment in case of REC/SPM
Study Duration and Schedule	**Pilot phase:**<br>Duration: 2 years (12 months recruitment, 12 months FU)<br>Planned First-Participant-In: Q4/2022<br>Planned Last-Participant-Out: Q4/2024<br>Data of this pilot study will be re-used in a subsequent, pivotal trial if key features of the study remain the same, either by pooling the data or using a Bayesian framework, with the main endpoint comparing death from any cause up to 5 years (5-year RMST) of the two study arms.<br>**Main study:**<br>Estimated duration: 8 years (recruitment period: 3 years, FU period: 5 years)<br>Planned First-Participant-In: Q4/2023<br>Planned Last-Participant-Out: Q4/2031
Investigator(s)	Please find the comprehensive list of all participating investigators in **Appendix 4**: List of involved institutions
Study Center(s)	Pilot phase: 3 centers in Switzerland<br><br>Main study: 34-42 centers expected from Switzerland, Germany, Austria, Belgium, France, Netherlands and Italy
Data privacy	A unique participant identification number will be attributed to each participant registered for the trial. The names of the participants will not be disclosed to persons outside of each participating site.
Ethical consideration	Given the lack of prospective studies and evidence-based data, there is a pressing need for a representative randomized trial to address the many issues related to the FU schedule for HNC patients. The trial will also allow assessment of the impact of the FU exams on QoL, an important issue that current guidelines fail to account for. The study will include only adult participants without any vulnerable populations.
GCP Statement	This study will be conducted in compliance with the protocol, the current version of the Declaration of Helsinki, the ICH-GCP, the HRA as well as other locally relevant legal and regulatory requirements.

2 BACKGROUND AND RATIONALE

2.1 Disease background

Head and neck squamous cell carcinoma (HNSCC) is the 6th most common non-skin cancer, arising from the oral cavity, pharynx and larynx. There are estimated 900’000 affected patients with over 400’000 deaths per year worldwide (Europe: 150’000 new cases per year). [1] Tobacco

and alcohol are the main risk factors. More recently, it was discovered that oropharyngeal HNSCC can be induced by human papillomavirus (HPV).

The main types of treatment are surgery, radiotherapy (RT) alone and radiation therapy with chemotherapy (RTCX). The optimal combination of these three modalities mainly depends on the site of the cancer and its stage. In general, early-stage HNSCC (stages I-II, 30% of cases) are treated with one single modality, either surgery or RT. Advanced stage diseases (stages III-IVA/B, 70% of cases – excluding stage IV HPV-positive oropharyngeal cancer) often require a combination of the above-mentioned treatment modalities. In those cases, therapeutic options are most often concomitant RTCX, or surgery followed by adjuvant RT(CX).

The curative rate for early-stage cancer is up to 80-95%. On the contrary, despite technological advances in surgery, RT and the developments of new types of systemic treatment, about 50-60% of the patients with locoregionally advanced HNSCC develop a locoregional recurrence (REC) mainly within 2 years [2-5]. In addition, 20-30% of patients with locoregionally advanced disease develop distant metastases. There is a cumulative +2-4%/year risk of second primary malignancies (SPM) due to common carcinogenesis and in-field cancerization (mostly for HPV-negative carcinoma) [6-8]. The most common sites of SPM are the lung (60%) and the superior aero-digestive tract (20%). Important differences exist in the clinical behaviour (response, pattern, and timing of REC/SPM) between HPV-positive and -negative oropharyngeal cancer. Patients with HPV-positive oropharyngeal cancer have a lower risk of REC/SPM [9].

According to an analysis of data derived from the Surveillance, Epidemiology, and End Results (SEER) limited-use database, the overall 5-year relative survival rate of head and neck cancer (HNC) patients treated between 2002-2006 was 65.9% [10].

2.2 Background in follow-up for head and neck cancer patients

The overall prevalence of patients living with the diagnosis of HNSCC is increasing in industrialized countries, thus management of survivors represents a daily practice challenge for both oncologists and primary care physicians. HNC patients have a poor prognosis, suffer from treatment-related toxicities and morbidities ((i.e., problems with swallowing and speaking) and are often disfigured. Therefore, the aim of a FU in this population in the curative setting is 5-fold: 1) evaluation of treatment response; 2) timely identification of tumor REC and SPM to allow a curative salvage treatment; 3) detection and management of treatment-related early and late complications; 4) restore nutritional status, and 5) providing psychosocial support to patients and their families [11]. But FU should avoid unnecessary investigations that may cause morbidity, discomfort or stress to the patient and may have significant financial implications without impact on survival. These goals are accepted worldwide, but there is neither consensus nor level 1 evidence on how to ideally achieve them.

Most guidelines advocate regular clinical visits with a head and neck (H&N) exam including optic evaluation of the nose, pharynx and larynx. Systematic evaluation of radiation-induced early and late toxicity, rehabilitation of functional loss (voice, breathing, swallowing), pain and nutritional management and psychosocial support, including tobacco and alcohol cessation, should be addressed during each FU visit [12]. After baseline imaging (magnetic resonance imaging (MRI), computed tomography (CT)) within 3-4 months recommended after surgery with altered anatomy and/or definitive RTCX in patients with locoregionally advanced cancer, systematic reimaging is deemed controversial. 18FDG-positron emission tomography (PET/CT) is recommended within 3-6 months after definitive or adjuvant RTCX for assessment of treatment response and to identify tumor persistence/progression; last one predicting impaired oncological outcome [13]. For further surveillance until 5 years and long-term, the value of routine locoregional and systemic imaging is not clear in asymptomatic patients. A chest CT scan should be performed if chest imaging is

requested (only 33% of intra-thoracic lesions picked up by chest CT scan were also detected by chest X-ray) [14]. However, guidelines for FU differ in terms of visit frequency, imaging and duration. There is a general agreement that FU should be more intensive during the first 2 years post-treatment (**Table 1**).

Table 1. Published guidelines on FU intervals

Study **Period**	**Year 1**	**Year 2**	**Year 3**	**Year 4**	**Year 5**	**>5 Years**
ASHNS (ASHNS, 1999) [15]	1-3	2-4	3-6	4-6	4-6	12
BAHNO (BAHNO, 2001) [16]	1-1.5	1-1.5	3	6	6	12
DCHNO (DCHNO, 2002) [17]	2	3	4	6	6	-
Lester and Wight, 2009 [18]	1	2	3	4	6	12
Digonnet et al., 2013 [12]	2-3	2-3	3-6	3-6	6	12
AIMO/AIRO (AIMO/AIRO, 2016) [19] [20]	1-3	3	3-6	6	6	12
FOS (FOS, 2016) [21]	2	3	4	6	6	12
SSORL (SSORL, 2019) [22]	1-3	1-3	4-6	4-6	4-6	(12))
EHNS/ESMO/ESTRO (EHNS/ESMO/ESTRO, 2020) [23]	2-3	2-3	6	6	6	12
NCCN (NCCN, 2022) [24]	1-3	2-4	4-6	4-6	4-6	6-12

Suggested interval of FU visits in months by selected authors and national committees. AIMO = Associazione Italiana Oncologia Medica; AIRO = Associazione Italiana Radioterapia Oncologica; ASHNS = American Society of Head and Neck Surgeons; BAHNO = British Association of Head and Neck Oncologists; DCHNO = Dutch Cooperative Head and Neck Oncology Group; EHNS = European Head and Neck Society; ESMO = European Society for Medical Oncology; ESTRO = European Society for Radiotherapy and Oncology; FOS = French ORL Society; NCCN = National Comprehensive Cancer Network, SSORL = Swiss Society of Oto-Rhino-Laryngology, Head and Neck Surgery

The recommendations from the NCCN Guidelines Version 1.2021 – Head and Neck Cancers showed in the following FU recommendations recapitulate many national and international FU schedules [13].

FOLLOW-UP RECOMMENDATIONS[a]
(based on risk of relapse, second primaries, treatment sequelae, and toxicities)

- H&P exam (including a complete head and neck exam; and mirror and fiberoptic examination):[b]
  - Year 1, every 1–3 mo
  - Year 2, every 2–6 mo
  - Years 3–5, every 4–8 mo
  - >5 years, every 12 mo
- Imaging (See Principles of Imaging, IMG-A)
- Thyroid-stimulating hormone (TSH) every 6–12 mo if neck irradiated.
- Dental evaluation[c] for oral cavity and sites exposed to significant intraoral radiation treatment.
- Consider EBV DNA monitoring for nasopharyngeal cancer (category 2B).
- Supportive care and rehabilitation:
  - Speech/hearing and swallowing evaluation[d] and rehabilitation as clinically indicated.
  - Nutritional evaluation and rehabilitation as clinically indicated until nutritional status is stabilized.[d]
  - Ongoing surveillance for depression (See NCCN Guidelines for Distress Management).
  - Smoking cessation[e] and alcohol counseling as clinically indicated.
  - Lymphedema evaluation and rehabilitation, as clinically indicated. (See LYMPH-A in the NCCN Guidelines for Survivorship).
- Integration of survivorship care and care plan within 1 year, complementary to ongoing involvement from a head and neck oncologist (See NCCN Guidelines for Survivorship).[f]

After 6 months until 5 years post-treatment, the Swiss FU Guidelines (Working Group of SSORL, 2019) recommend routine imaging within the first 2 years after treatment depending the stage and site of the primary tumor [22]. The NCCN Guidelines Version 1.202212 – Head and Neck Cancers state that there are no consensus guidelines on modality and frequency of routine post-treatment imaging in the asymptomatic patient. The NCCN Guidelines of Lung Cancer Screening (NCCN Guidelines, Version: 1.20222) instead recommend annual lung screening with a low-dose CT scan in high-risk candidates including HNC and smoking habits [25].

2.3 Rationales for the trial

Head and neck cancer patients represent a heterogeneous group, and there is currently an ongoing debate to determine the optimal frequency, content and duration of FU care, although prospective studies and evidence-based data do not exist. There are two major problems: 1) the lack of consolidated data about FU (procedures and timing) among different institutional, national and international guidelines; and 2) the need of a tailored approach that consider different prognostic factors and patients' performance status/age. One crucial unsolved question is whether an early detection of REC/SPM in asymptomatic patients has an impact on survival and quality of life (QoL).

2.4 Routine systematic follow-up

There are only retrospective observational studies addressing this topic (**Table 2**). Routine surveillance has been associated with a survival benefit in only two observational retrospective studies for REC diagnosed at routine FU visits when compared with those patients who self-referred with REC symptoms [26, 27]. Most other studies however have not observed a difference in survival between routine FU and symptom-driven self-referral detected REC, nor found a correlation between FU intensity and survival [28-34]. Some studies even challenge the role of the physician and imaging methods in identifying oncological events, suggesting that patients themselves may most efficiently detect REC [30, 35-37]. In the study by Pagh et al. (2013), they found that only few relapses are found in asymptomatic patients at routine FU, with one silent recurrence detected per 99 visits. Ilmarinen et al. (2018) showed, that all REC were identified in self-referral patients with new symptoms, and that no REC was detected during visits in asymptomatic patients [38]. In another study following the NCCN-recommended clinical surveillance in HPV-positive oropharyngeal cancer, the authors showed that using this FU regimen almost never allowed detecting an asymptomatic REC [39]. In the study by Jung et al. (2014), they could not show any impact of REC detection method (physician/routine imaging) on salvage treatments' success rate [40]. One explanation for the lack of a survival benefit may be the high proportion of REC that are symptomatic, with silent REC in only about 1.2% of patients [30, 41]. But there is another retrospective study demonstrating that 23.3% of patients with REC were asymptomatic at diagnosis [42]. Besides that, a new strategy has recently emerged that

may contribute to the optimization of FU concept. Electronic Patient-Reported Outcomes (ePROs), allowing real-time symptom monitoring and even offering a survival benefit as demonstrated in a randomized prospective trial from Memorial Sloan Kettering Cancer Center reported increased 1-year survival of patients with advanced solid (not site or histology-specific) tumors (75% vs. 69%, p=0.05) when patients reported symptoms via a web-based ePROs which triggered automated alerts [43] and in another study including patients with advanced non-progressive stage IIA to IV lung cancer, in which the median 2-year survival rose from 13.5 to 22.5 months (p=0.005) in the arm utilizing web-based PROs monitoring during chemotherapy versus standard scheduled imaging after treatment to detect symptomatic REC [44].

Table 2. Published studies on FU

Author	n	Routine FU	Recurrence / Second Primary	Results / Oncologic Outcome
Boysen, 1992 [45]	661	Clinical examination	185 LRR<br>35 Mets	No difference in OS between routine FU and self-referral
De Visscher, 1994 [26]	428	Clinical examination + yearly Thorax Xray	154 LRR<br>56 Mets<br>58 SPM	Mean survival better with routine FU than self-referral
Schwartz, 2003 [33]	100	Clinical examination + yearly H&N CT scan	22 LRR	No difference in OS between routine FU and self-referral
Kissun, 2006 [32]	278	Clinical examination	48 LRR<br>6 Mets	No difference in OS between routine FU and self-referral
Ritoe, 2007 [27]	402	Clinical data	94 LRR<br>15 Mets<br>47 SPM	Life expectance better with routine FU than self-referral, limited impact of life expectance on elderly patients
Agrawal, 2009 [28]	-	Clinical data	105 LRR/Mets	No difference in OS between compliant and noncompliant patient groups with recommended FU
Flynn, 2010 [30]	223	Clinical examination + yearly Thorax X-ray	49 LRR<br>16 Mets	No difference in OS between routine FU and self-referral
Kothari, 2011 [36]	1039	Clinical examination	156 LRR/Mets	Most REC identified in symptomatic patients
Jung, 2014 [40]	520	Clinical examinations + H&N imaging (twice / year within first 2 years, then annually)	86 LRR<br>9 Mets<br>6 SPM	No impact of REC detection method on salvage success rate
Pagh, 2013 [37]	619	Clinical examination (cross sectional study)	29 LRR/Mets	Most REC identified in symptomatic patients; more salvage therapies possible in asymptomatic patients
Zatterstrom, 2014 [46]	537	Clinical examination	149 LRR<br>19 Mets<br>28 SPM	Most recurrences identified by self-referral patients with symptoms (78%); only 22% of REC detected in asymptomatic patients at scheduled FU visits. No difference in DFS between routine FU and self-referral
De Zoysa, 2017 [47]	47	Clinical data: enhanced “traffic light” FU (red flag signs) vs.	1 LRR	0% vs. 42% non-compliance in enhanced vs. traditional clinician-arranged FU. All REC identified after noticing red flag signs.

		traditional clinician-arranged FU		
Boysen, 2016 [35]	1678	Clinical data	525 LRR/Mets	Most REC identified in symptomatic patients; better prognosis in patients with subjective symptoms in oral cavity and larynx cancer
Ilmarinen, 2018 [38]	153	Clinical examination	3 LRR<br>1 Mets	All recurrences identified in self-referral patients with new symptoms; no REC detected during visits in patients without symptoms
Imbimbo, 2019 [31]	326	Clinical examinations + H&N imaging (twice/year within first 2 years, then annually within the following 2 years) + whole-body CT (yearly during 5 years)	38 LRR<br>44 Mets<br>24 SPM	Clinical and radiologic FU allowed identification of a higher number of REC/SPM than the symptom-driven monitoring; no difference in median OS
Masroor, 2019 [39]	233	NCCN recommended post-treatment surveillance in HPV-pos. oropharynx cancer	22 LRR/Mets	NCCN-recommended clinical surveillance regimen almost never detects an asymptomatic REC; adherence to this schedule is not associated with OS improvements; loco-regional REC were not detected beyond 2 years; support of reduction in the number of post-treatment clinical surveillance visits for patients with HPV-associated oropharyngeal cancer

DFS = disease-free survival; FU = follow-up; H&N = head and neck; LRR = loco-regional relapse; Mets = Metastasis; n = number of patients; OS = overall survival; REC = recurrences; SPM = second primary malignancy

It can be argued that the reason why the majority of REC are symptomatic is the insufficient detection capacity of a physical examination supplemented by endoscopy as indicated in early-stage disease and/or the inadequacy of the time interval between the visits. This seems to be a credible statement since very small neoplastic changes remain clinically silent. In this respect, biochemical tumor markers are commonly prescribed in oncology practices with varying degree of supporting evidence. In HNSCC, this diagnostic approach lacks sufficient sensitivity until now, with the only possible exceptions, Epstein-Barr virus deoxyribonucleic acid (DNA) analysis in nasopharyngeal cancer survivors and emerging new data on HPV cell-free DNA monitoring in viral-related oropharyngeal cancer, not yet standardized for routine clinical use [48-50].
Second primary malignancies are developing in 2-4% of HNSCC patients yearly and have a negative impact on their survival [51]. The etiologic shift of oropharyngeal cancer requires to investigate more for distant metastases than for SPM in HPV-positive cases [52]. Since some estimates show that by 2030 the incidence of HPV-positive cancers may be superior to that of negative ones, the applicability of current FU regimens is also called into question.
Follow-up guidelines in HNC patients have not been systematically and prospectively investigated. Although on a purely intuitive base, it could be assumed that regular scheduled physical and radiological exams would be beneficial concerning the detection of REC/SPM, available data do not allow answering this very basic question. In contrary, in a recent prospective diagnostic cohort study by Malik et al. (2020), they found that a symptom-based telephone questionnaire had good sensitivity (90%) and negative predictive value (99.3%) for detecting REC in patients with oral cancer on FU evaluation after completion of definitive treatment [53]. This may question the necessity of systematic physical exams. It has been shown, however, that salvage treatment has

a higher success rate when REC is detected at an early stage [54-56], as curative treatment options are limited in this setting and may not be available for REC/SPM in an advanced stage. This highlights the importance of early REC/SPM detection and of an efficient FU.

2.5 Imaging

There is persisting controversy on the use of periodic radiological exams in the FU of HNSCC patients. Early detection of a locoregional REC may be more beneficial than detection of asymptomatic distant metastatic disease, because curatively intended treatment may still be possible. In the post-treatment setting, MRI is preferred to CT scan because of superior visualization of soft tissue and skull base anatomy [57, 58]. However, the use of periodical MRI in the detection of REC recurrence is unclear. One study showed a sensitivity of only 50% and specificity of 83%, arguing that MRI in patients with low suspicion of REC is of limited value [59]. Another study did show much better accuracy of MRI when using diffusion-weighted sequencing techniques [60]. Sensitivity and specificity were 94.6% and 95.9%, respectively. Al-Shwaiheen et al. (2015) showed that out of an original cohort of 533 oral cavity squamous cell carcinoma patients, 46 patients, who were disease-free 6 months after treatment, had undergone 108 routine H&N-MRIs from 6 to 48 months after surgery without the presence of concurrent suspicious symptoms or signs and had 6 months of subsequent follow up. Only 1 out of 46 (2.2%) had a true positive regional recurrence. Ten out of 46 (21.7%) patients experienced a false positive locoregional finding. They concluded that routine H&N-MRI for locoregional surveillance of oral cavity squamous cell carcinoma, when used in patients without concurrent suspicious symptoms or exam findings over 6 months since treatment, may be unnecessary and costly, given the very low rate of recurrence and high false positive rate [61].
PET/CT after RTCX in advanced-stage disease within the post-treatment 3-6-month period offers notable benefits. It was significantly more sensitive than regular FU for the identification of disease relapse, in several retrospectives and prospective analyses [62-65]. Kao et al. (2009) showed that 2-year progression-free survival and 2-year OS rates were significantly different between patients who had a negative and those with a pathologic PET/CT result within 6 months of the completion of RT (93% vs 30% and 100% vs 32% ($p<0.001$), respectively) [62]. Ho et al. (2013) studied the impact of PET/CT surveillance for detecting HNSCC REC at 12- and 24-months post-treatment [66]. In a 10-year retrospective analysis of 284 patients, the PET/CT detection rate of occult REC was 9% at 12 months and 4% during the second year, without 3-year disease-free (DFS) and OS difference compared to a larger group of patients who did not receive annually PET/CT. Similarly, Dunsky et al. (2013) confirmed the role of PET/CT in early detection of asymptomatic disease, but the outcome of those patients with identified REC remained poor [67]. In 2016, Mehanna et al. demonstrated in the randomized PET-NECK trial, that survival was similar after PET/CT-guided surveillance 12 weeks post-RTCX and those who underwent planned neck dissection. However, surveillance resulted in considerably fewer operations, and it was more cost-effective [68]. In another study in HPV-associated oropharyngeal cancer, Corpman et al. (2019) could demonstrate that the PET/CT is very effective as a rule-out test for REC, however, resulting in many equivocal or false-positive results that precipitate clinical decision-making dilemmas and without OS difference when compared with REC detected clinically [69].
Standard orthogonal chest radiography for detection of pulmonary metastases or SPM has been discarded in most national guidelines due to its low sensitivity [70]. The use of chest CT scan has been recommended instead by Hsu et al. (2008), but the interval and duration of screening remain to be evaluated [71]. Results of the National Lung Screening trial suggest a potential reduction in mortality of lung cancer by means of screening patients at high risk for lung cancer with low-dose helical CT scan, which was also confirmed by the NELSON trial, but this has not been evaluated in the FU of HNC patients [72-74]. The National Lung Screening trial reported that just 3.6% of positive screening tests ultimately led to the diagnosis of cancer, at the cost of a very large number of FU imaging studies, procedures, and complications [72, 73]. Other studies confirmed this high rate of false-positive findings in imaging after HNSCC treatment, entailing additional unnecessary exams that are strenuous to patients and costly to healthcare [57, 75]. Padegar et al. (2015)

compared the survival of HNSCC patients with second primary lung cancers with the survival of early lung cancer-only patients and found a worse survival outcome after early second primary lung cancer in HNC patients (median survival: 22 months) than in patients with lung cancer only (median survival: 38 months) [76].
Resumed to that is a recent retrospective study of 326 patients in which a clinical and radiological FU involving periodic CT scan, MRI, and PET/CT identified more REC in the asymptomatic phase than were patient-detected cases, which were symptomatic at a scheduled appointment or revealed during an unplanned, symptom-driven consultation. However, the proportion of patients eligible for a curative treatment remained comparable as well as their survival outcomes [31].
In conclusion, while the benefit of imaging to confirm response to treatment has been well demonstrated, the role of routine imaging after 6 months post-treatment in the FU of HNC patients after proven complete disease remission remains unclear.

2.6 The patient's perspective

The frequent physical and radiological exams may be a major logistical, psychological and also a financial burden for the patients. During these visits, patients are confronted with their fear of REC. As a result, they may be worried about what examinations and tests may show, and also by the possibility of seeing an unknown physician in high-volume centers [77-80]. On the other hand, a negative image study might improve the QoL due to "objective" reassurance. Nevertheless, fear of REC is a common feature among HNC patients with a proven impact on QoL [77, 78, 81-84]. A survey study (101 patients) performed by our team assessing patients' preferences concerning post-treatment surveillance (choice between 4 different FU schemes: non-intensive, de-escalated +/- periodic imaging, conventional intensive +/- periodic imaging) and fear of REC demonstrated an elevated fear in 36.6% of treated HNC patients [85]. Concerning the preferences of FU visits, the survey clearly showed that the majority of patients (89%) wish to have regular FU exams. The majority (85.1%) of the patients favoured a FU scheme, which includes periodic imaging. Concerning the frequency of the exams, however, 57% preferred de-escalated schedules with less visits than currently advised in national and international guidelines. This highlights the fact that although patients value periodic exams, they are a certain burden to them. The feeling of reassurance and satisfaction with the care they get may in some cases be counterbalanced by harmful aspects of close surveillance including imaging-associated distress, ultimately leading to a worse QoL, excessive radiation exposure, false positivity causing unnecessary additional work-up, low cost-effectiveness, and even distraction from other recommended FU procedures [86, 87].
Patients with locoregionally advanced HNC experience important impair to their QoL after treatment. A recent study regarding the 2-year QoL outcome reported significantly impaired functioning scores, as well as significantly increased symptom burden in the corresponding scales of the European Organisation for Research and Treatment of Cancer, Core Quality of Life of Cancer Patients (EORTC QLQ-C30) 24 months after the end of RT(CX), compared to a population reference group [88]. Twelve and 24 months after RT(CX), QoL improved. But still, HNC survivors have a substantial need for management of sequelae. In this context, a centralized routine FU may still be worthwhile.
The impact of an individualized de-intensified FU scheme with less planned clinical and imaging exams on patients' QoL is unclear and warrants further research.

2.7 Costs

In addition to the unanswered medical controversies, cost evaluations of FU are rare. Given that FU in HNC according to current guidelines extends over a minimum of 5 years, it entails important financial consequences for both, patients and the healthcare system, which have to be weighed against its medical benefits. While in other solid cancer (breast, colon and lung cancer) a more intensive FU did not offer a survival advantage with unjustified costs, in HNC FU, costs have not been deeply studied. Despite HNSCC are expensive to treat, have a high morbidity, and of those individuals that survive, only 48% return to work, research on the direct and indirect cost burden

are limited. In 2001, van Agthoven et al. reported the costs of 10-year FU, calculating retrospectively on patients treated between 1994 and 1996 [89]. In total, average costs per new patient were Euro 31,829, which covered discounted costs of treating the primary tumor, costs of treating recurrent tumors in 40% of all patients, and the costs of 10 years of FU. A systematic literature review (2003-2013) on more recent resource use and costs associated with the diagnosis and treatment of HNC divides the economic burden into direct (treatment-related) and indirect costs (patient time reduced workforce participation) [90]. Direct costs depend on the treatment modality and may range from USD 50’444 (surgery) to USD 334’754 (IMRT and concomitant chemotherapy) in T1-T3 laryngeal cancer. The average productivity losses per person attributable to temporary and permanent work absence and reduced work hours ranged from USD 101’187 to 222’000. Cost analyses focusing specifically on FU have not been performed.
Given the lack of evidence on how to follow curatively treated HNC patients and mostly no observed difference in OS between routine FU with/without imaging and symptom-driven self-referral-detected REC in retrospective studies from the literature, and no existing comparable trial with the same design and outcome measures, there is a pressing need for this representative multicenter, prospective study comparing an individualized de-intensified FU strategy with a conventional (standard) one, with increased patient involvement in both arms, in order to define an oncological, patient-adapted, and cost-efficient FU strategy. Our study will deliver long awaited results to modify existing or design new, evidence-based national and international FU guidelines tailored for HNC patients. The study's results could change the practice concerning the HNC FU worldwide.

2.8 Other existing trials

Two ongoing trials exist with an escalating study design using PET/CT yearly in the more intensive FU schedule: 1. Health and Economic Outcomes of Two Different Follow up Strategies in Effectively Cured Advanced Head and Neck Cancer (HETeCo); Fondazione IRCCS Istituto Nazionale dei Tumori, Milano, ClinicalTrials.gov Identifier: NCT02262221; 2. Randomized Multicentric Comparative Study Between a Conventional and an Intensive Follow up Strategy (PET/CT 1/year for 3 years after Treatment of a Head and Neck Squamous Cell Carcinoma (SURVEILL'ORL), Gustave Roussy, Cancer Campus, Grand Paris; ClinicalTrials.gov Identifier: NCT03519048. The first one has a smaller sample size (n=330) and the recruitment status is unknown. The primary outcome measure is the evaluation of cost-effectiveness within 3 years. The primary outcome measure of the SURVEILL’ORL study is 5-year OS, but also, contrary to our trial, with an escalating design.
Another study, the PETNECK2, is a NIHR PGfAR funded Programme to develop a new way to FU patients, guided by their symptoms, and compare it to the current way. The new patient-initiated FU method involves low risk HNC patients having a PET/CT scan 1 year after finishing treatment. If no cancer is detected, they will be educated, by a nurse, about what symptoms of recurrent cancer to look out for. The information will also be given in print and electronically (via a mobile App and website). An ‘open urgent appointment’ guarantees review by their clinical team within 2 weeks if they develop worrying symptoms, instead of regular clinic visits (PET/CT guided, symptom-based, patient-initiated surveillance versus clinical FU in advanced head neck cancer (PETNECK 2), Institute of Head and Neck Studies and Education, University of Birmingham, NIHR200861).
A trial comparing two surveillance approaches for people who have received treatment for HPV-associated HNC and show no signs of disease is recruiting (Memorial Sloan Kettering Cancer Center, ClinicalTrials.gov Identifier: NCT05048459). They compare a surveillance telemedicine approach with a standard FU strategy. The primary outcome measure is progression-free survival after 2 years. The sample size is much smaller (n=160) and they include only patients with HPV-positive cancer, which is not comparable with our foreseen trial.

3 STUDY OBJECTIVES AND DESIGN

3.1 Hypothesis and primary objective

It is hypothesized that an individualized de-intensified FU strategy does not increase the risk of death as compared to a conventional (standard) FU strategy in HNC patients after curatively intended treatment and with radiological and clinical complete remission from the 3rd to 6th month and at the 6th month (+/- 4 weeks), respectively. In addition, it is hypothesized that symptom-driven visits in well-instructed patients with regular recording of signs and symptoms have a comparable diagnostic yield in the detection of REC or SPM than systematically scheduled in-person visits.

3.1.1 Objectives of the pilot phase

Primary objective

The primary objective of the pilot phase of this trial is to evaluate the feasibility of recruiting and randomizing patients with complete remission 6 months after treatment of head and neck squamous cell carcinoma (HNSCC) to a de-intensified and conventional (standard) FU.
Secondary objectives
The secondary objective is to investigate adherence to the two different FU strategies. Good compliance is defined as the realization of the planned/organized visits and exams at the planned time +/- 4 weeks as well as regularly recording of signs and symptoms in the patient-reported outcomes (PRO) questionnaire.
Section 0 below describes the respective progression criteria for moving to the main trial.

3.1.2 Objectives of the main study

Primary objective

The primary aim of the study is to determine whether an individualized de-intensified approach to surveil patients is non-inferior to a conventional FU in HNC with regard to 5-year restricted mean survival time (RMST).
Secondary objectives

Comparison of the impact of a de-intensified to a conventional (standard) FU from the date of randomization up to 1 and 5 years, or until the date of multidisciplinary tumor board decision for the treatment strategy concerning the first REC/SPM after the primary treatment or until death in regard to:

- HNC-specific survival, any cancer-specific survival
- Detection rate of REC/SPM (as “time to endpoint”)
- Incidence, site and stage of REC
- Incidence, histopathology, site (head and neck, lung, etc.) and stage of SPM
- Rate of REC/SPM amenable to curatively-intended therapy
- Type and the actual number of FU visits
- Assessment of patients’ compliance based on the rate of missed scheduled appointments → accepted or refused/ignored recall appointments within 4 weeks
- Assessment of patients’ additional visits between the scheduled visits
- Fear of REC
- Health-related QoL
- Healthcare costs

Descriptive objectives

- Late toxicities (types and grading) and outcome
- Characteristics of REC/SPM (diagnostic modalities that lead to detection of REC/SPM)
- Adherence to signs/symptom monitoring and visits that are triggered by the PRO

3.2 Primary and secondary endpoints

Endpoints relate to patient-level assessments. Estimands and effect measures are provided in section 3.3.
Selection of endpoints was based on clinical relevance, aspects to substantiate safety of the individualized de-intensified schedule, procedural aspects to assess adherence to the different FU schedules, and to allow fulfilling other secondary objectives. *Outcomes* related to the descriptive objectives are not listed, but the data that will be collected to allow to address them is shown in section 4. It should be noted that additional secondary or exploratory outcomes might be added after the pilot phase based on input from participants. A search in the COMET database (https://comet-initiative.org/Studies) revealed no available Core Outcome Set. A MEDLINE search and a search on the website of the US National Cancer Institute also revealed no recommendations (that are based on solid methodology) on endpoints for HNC clinical trials.
If not stated otherwise, the outcome measurements relate to the time period of up to 5 years after randomization.

3.2.1 Main study

3.2.1.1 Primary endpoint

Death from any cause.
Defined as the time interval between the date of randomization and the date of death up to 5 years i.e. patients who are not known to have died will be censored at the date of the last time point at which they were known to be alive (quantified by the 5-year RMST).

3.2.1.2 Secondary endpoints (all at 5 years)

- Death from HNC (time to HNC-specific death)

Defined as the time interval between the date of randomization and the date of death due to original primary disease up to 5 years. Patients who are not known to have died will be censored at the date of the last timepoint at which they were known to be alive (quantified by the 5-year RMST). Death from causes other than the original primary disease is considered as a competing risk event in the analysis of this endpoint.

- Death from any cancer (time to cancer-specific death)

Defined as the time interval between the date of randomization and the date of death due to any type of cancer up to 5 years. Patients who are not known to have died will be censored at the date of the last timepoint at which they were known to be alive (quantified by the 5-year restricted mean survival time). Death from causes other than cancer is considered as a competing risk event in the analysis of this endpoint.

- First biopsy-proven REC or SPM *(time to REC/SPM)* based on participating centers' assessment according to the UICC/TNM 8th ed.

Note: In special scenarios, such as co-morbidities, patient's rejection or other medical conditions, a biopsy can be omitted if there is a consensus for a REC/SPM at the participating centers' multidisciplinary tumor board based on the clinical and/or radiologic findings. Time to REC/SPM is defined as the time interval between the date of randomization and the date of REC or SPM (whatever comes first) up to 5 years. Patients who are not known to have REC/SPM will be censored at the date of the last time point at which they were known to be REC/SPM-free (quantified by the 5-year RMST).

- General health-related QoL as assessed every 6 months over 5 years by means of the

European Organisation for Research and Treatment of Cancer, Core Quality of Life of Cancer Patients (EORTC QLQ-C30).

- Head and neck cancer-specific health-related QoL as assessed every 6 months over 5 years by means of the European Organisation for Research and Treatment of Cancer, Head and Neck Cancer Module (EORTC QLQ-HN43).
- Compliance with scheduled FU assessments

Patients' compliance is defined as the number of completed visits/exams out of all scheduled visits/exams (self-assessments, remote, or on-site). Rescheduling of visits by the patients within +/- 4 weeks of the scheduled visit window does not count as non-compliance. The number of recalls will be recorded and reported.

- Number of regularly scheduled and non-scheduled in-person visits
- Number of in-person visits triggered by the recommendation of the PRO
- Number of self-referral in-person visits
- Number of any in-person visits
- Fear of REC assessed monthly
- HNC-specific healthcare utilization
  Although we aim to compare healthcare costs, assessing and measuring actual healthcare costs is deemed not feasible. We, therefore, use HNC-specific healthcare utilization as a proxy. Country-specific unit costs over the 5 years will be used to arrive at a single number that allows quantitative comparison between trial groups. Note: Data on health-related quality of life as measured by EuroQol five dimensions questionnaire (EQ-5D(-5L)) will also be collected once a year over 5 years. However, this data will only be analyzed as part of the cost effectiveness/utility analyses and not as separate endpoint (as there are more specific instruments used as described above).

Description on:

- Characteristics of REC/SPM (diagnostic modalities that lead to detection of REC/SPM, incidence, site, stage, and whether it is amenable to curatively-intended salvage therapy)
- Type and grading of specific treatment-related adverse events and outcome. Treatment-related adverse events will be assessed and graded using the Common Terminology Criteria for Adverse Events (CTCAE, version 5.0) at baseline and every visit (scheduled and non-scheduled) in both study arms.
- Adherence to electronical signs/symptom monitoring and triggers that lead to recommendation for a control earlier than scheduled visit with the visit organized by the participating center

All assessments for secondary endpoints will end at 5 years FU, the date of multidisciplinary tumor board decision for the treatment strategy concerning the first REC/SPM after the primary treatment or at death.

3.2.2 Pilot study

Besides the endpoints of the main trial, we will collect additional data to achieve the objectives of the pilot phase. Some of these are not patient-level assessments in the narrow sense but are still provided here:

- Eligibility rate defined as the proportion of all eligible patients (all eligibility criteria met but signed informed consent) out of all screened patients
- Consent rate defined as the number of consenting patients out of all eligible patients (all eligibility criteria met but signed informed consent)
- Overall accrual per month
- Motivation for participation or not consenting based on a participation questionnaire

- Adherence to the randomized FU schedule

3.3 Estimands

3.3.1 Anticipated intercurrent events

- Death
- Definite discontinuation from randomized FU scheme due to non-medical reasons: participant definitely stops the randomized FU scheme but continues to attend study visits/survival status can still be elicited. Note that temporary (less than one year) non-adherence is not considered definite. The reason for stopping is not a medical one but rather a personal decision.
- Definite discontinuation from randomized FU scheme due to medical reasons: participant definitely stops the randomized FU scheme but continues to attend study visits/survival status can still be elicited. Note that temporary (less than one year) non-adherence is not considered definite. The reason for stopping is a medical one for example, a participant randomized to the experimental arm is not able to assess symptoms anymore because of dementia. The most likely reasons are:
  a. Cancer REC: any recurrence of a cancer, whether the *index* HNSCC or other
  b. Second primary malignancy: any newly diagnosed cancer different to the *index* HNSCC or previously diagnosed once

Note: Classification of whether a patient definitely stopped the assigned FU scheme will be done by at least two physicians (at least one head and neck surgeon and one radio-oncologist) at the study end. Given the intervention, no blinding is possible. The following will serve as guidance to determine whether a patient is non-adherent (note: sites will additionally indicate in the case report form whether a patient definitely stopped the assigned FU scheme and switched to a regular scheme):

- Control arm → regular visit only every 6 months and no regular imaging
- Experimental arm → scheduled (i.e. not symptom-driven or on request) visits every 3 months and regular imaging (i.e. not symptom-driven or on request)

Any other significant deviation from the assigned schedule not classified as definite stop or other intercurrent event for at least one year will be considered non-adherent. Note that patients who are non-adherent for at least one year but become adherent afterwards are still considered non-adherent for the analyses.
The following are not per se intercurrent events. Although they result in missing data, data would theoretically be available and interpretable. It is important that a careful consideration is made in the below cases whether an intercurrent event preceded one of the following. In general, if there is no explicit information that an intercurrent event happened, such participants should not be classified as having an intercurrent event.

1. Lost to FU: participant does not attend visits, is not reachable, and survival status cannot be confirmed by other means
2. Withdrawal of consent: participant (actively) withdraws consent with the consequence that no further data collection is allowed. It is important in such situations to elaborate with the participant whether she/he wants to stop the assigned follow-up scheme (intercurrent event) or wants to end trial participation only (see also sections 4.6).

3.3.2 Primary estimand

Table 3. Primary estimand

Type	Treatment-policy (intention-to-treat)
Population	All randomized participants in the group were allocated regardless of any protocol deviations or premature stop
Interventions to be	Control: Conventional FU entailing frequent clinical exams and

compared	routine imaging<br>Experimental: Individualized de-intensified FU entailing less frequent clinical exams and no routine imaging<br>Both regardless of any changes, interruptions, premature stopping
Endpoint	Death from any cause from randomization to up to 5 years of FU
Population-level summary (effect measure)	Difference in 5-year RMST
Intercurrent event(s)	Death: endpoint event<br>Any other: not considered<br>Lost to FU/consent withdrawal: censored at the last date known to be alive

3.3.3 Secondary estimands

Table 4. Secondary estimands

Type	Per-protocol I (while on treatment)
Population	All randomized participants that met all eligibility criteria and who were adherent to the allocated FU scheme as defined in the protocol
Interventions to be compared	Control: Conventional FU entailing frequent clinical exams and routine imaging<br>Experimental: Individualized de-intensified FU entailing less frequent clinical exams and no routine imaging
Endpoint	Death from any cause from randomization to up to 5 years of FU
Population-level summary (effect measure)	Difference in 5-year RMST
Intercurrent event(s)	Death: endpoint event<br>Definite withdrawal from randomized FU scheme for non-medical reasons: censored at the date of definite withdrawal<br>Definite withdrawal from randomized FU scheme for medical reasons: not considered as intercurrent event (ignored)<br>Lost to FU/consent withdrawal: censored at the last date known to be alive
Type	Per-protocol II (hypothetical)
Population	All randomized participants that met all eligibility criteria and who were adherent to the allocated FU scheme
Interventions to be compared	Control: Conventional FU entailing frequent clinical exams and routine imaging<br>Experimental: Individualized de-intensified FU entailing less frequent clinical exams and no routine imaging
Endpoint	Death from any cause from randomization to up to 5 years of FU
Population-level summary (effect measure)	Difference in 5-year RMST
Intercurrent event(s)	Death: endpoint event<br>Definite withdrawal from randomized FU scheme for non-medical reasons: censored at the date of definite withdrawal<br>Definite withdrawal from randomized FU scheme for medical reasons: censored at the date of definite withdrawal<br>Lost to FU/consent withdrawal: censored at the last date known to

	be alive FU

3.4 Study design

This is a randomized-controlled, multicenter, open-label non-inferiority trial with an explicit internal pilot phase. Establishing non-inferiority (trial success) is based on two criteria (dual-criterion design [91, 92]): 1) a traditional non-inferiority margin to which the one-sided confidence interval of the effect estimate is compared (statistical significance) and 2) a more clinically motivated decision value to which the effect estimate itself (point estimate) is compared. Both criteria have to be met to declare trial success. Although the design has been proposed for proof-of-concept (phase II) trials, it is well-suited for other settings as well: setting the non-inferiority margin is fraught with difficulties because the traditional criterion mixes type I error control and clinical relevance [92]. This is overcome by also setting a decision value to which the point estimate is compared. It might also be considered to be a more conservative approach because two criteria must be met to declare non-inferiority, which is a welcome property for definite trials that aim to inform clinical practice. The non-inferiority margin is set to a difference of 5 months between the two groups for the 5-year RMST and the decision value at 2 months.
Two interim analyses are planned in this study: A first interim (safety) analysis will be done after the first 100 patients in each group were followed for at least 18 months after inclusion (2 years after the end of treatment) (see section 5.1.3). Herein, we will assess whether omission of chest CT scan in the experimental arm is safe. The parameter for this analysis is isolated SPM or metastases of the lung. A second safety interim analysis is planned after minimal FU of 20 months of the last enrolled participant (see section 5.1.3).

Figure 1 below exemplifies the study design:

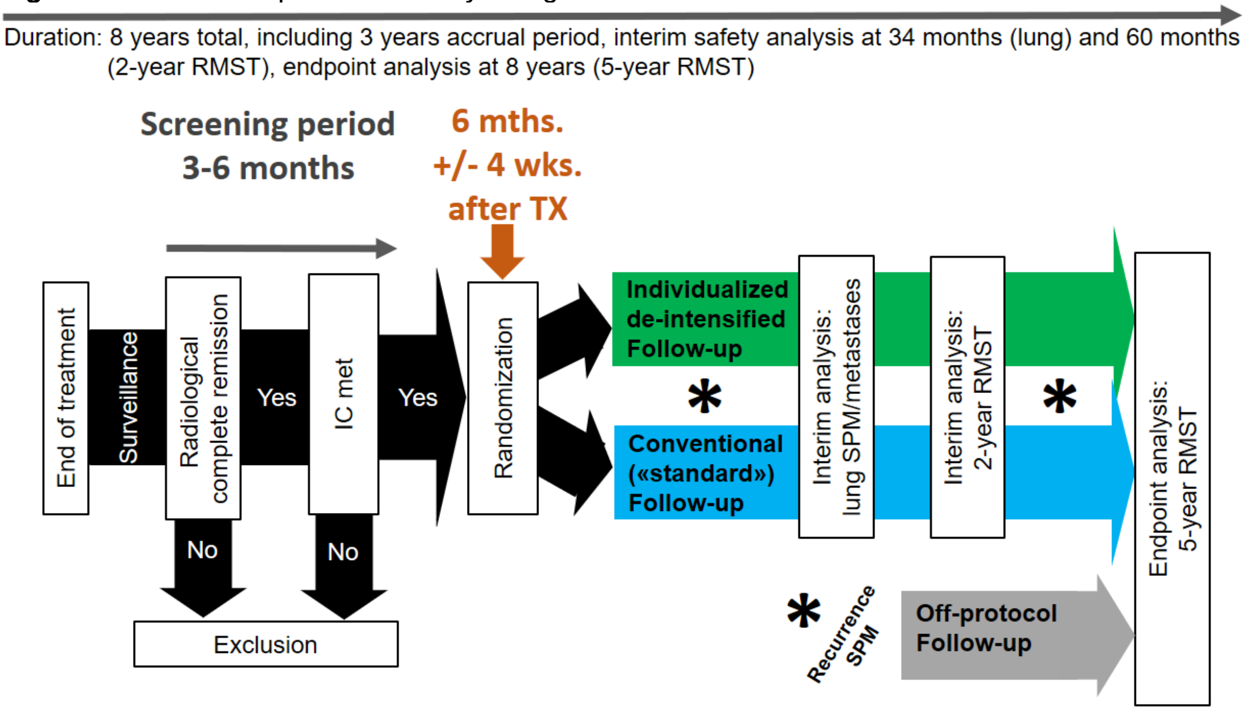

Flow chart of participants' recruitment and study procedure.
Abbreviations: IC=inclusion criteria; mths. = months; RMST = restricted mean survival time; SPM = second primary malignancy; TX = treatment; wks. = weeks

3.4.1 Internal pilot phase and progression criteria for moving to the main phase of the trial

Expected accrual time of the pilot phase is 1 year and of the main trial 3 years (4 years overall). Because feasibility is an important issue in this kind of study, we will evaluate it in the internal pilot phase in terms of recruitment and compliance to FU exams.
The study flow for individual participants in the pilot phase will exactly mimic the study flow of the main trial. The only difference will be the minimal additional data that will need to be collected on the participation questionnaire on motivation or not consenting.
Success of the pilot and therefore progression to the main trial will be based on observed enrolment and adherence. The criteria for enrolment were based on the number of sites planned for the main trial, the size of those sites, and the duration of the main trial. For the main trial, 34-42 sites committed to participate with an expected enrolment of >700 patients within three years. The sites (Bern, Luzern, Zurich) are committed to enrol 20 patients in one year during the pilot phase. As recommended, we will use a traffic light system [93].

Table 5. Traffic light system for moving from the pilot phase to the main phase

Traffic light*	Enrolment rate	Adherence
(green)	≥16 participants/year in two best enrolling sites	And ≥50% completeness (of all checklists) in recording signs and symptoms in experimental arm and adherence to the assigned FU scheme
(yellow)	≥12 participants/year in two best enrolling sites	And ≥40% completeness (of all checklists) in recording signs and symptoms in experimental arm and adherence to the assigned FU scheme
(red)	<12 participants/year in two best enrolling sites	Or <40% completeness (of all checklists) in recording signs and symptoms in experimental arm and adherence to the assigned FU scheme

*Green: continue (i.e. no concerning issues that threaten the success of the trial); Yellow: either adapt or continue with caution (i.e. remediable issues); Red: stop or at least halt (i.e. intractable issues that cannot easily be remedied). Although it is foreseen to have only two to three sites in the pilot, using enrolment in the two best enrolling sites allows also for more sites in the pilot.

3.4.2 Study duration

	**Pilot phase**	**Main phase**
Duration of the entire trial (recruitment, FU, analysis):	24 months	96 months
First participant-in to last participant-in/Recruitment period:	12 months	36 months
Follow-up per participant:	12 months (minimum)	60 months

3.5 Study intervention

3.5.1 Experimental arm - Individualized de-intensified FU

- Scheduled outpatient visits with clinical H&N examinations with endoscopy every 6 months during the 5 year of FU period.
- Monitoring of H&N REC/SPM symptoms by completing a monthly (p/e)PRO questionnaire with rating scale. In the pilot phase, the study team will call the participant to interview for the pPRO result and the study team will analyse if the PRO trigger indicating possible REC/SPM. In the main phase, the ePRO result will trigger an automated alert to the participant and to the site in conditions indicating possible REC/SPM. In case of possible REC/SPM, an 'open urgent appointment' will be arranged for the participant by the participating centers' team within 2 weeks.

3.5.2 Control arm - Conventional (standard) FU

- Scheduled outpatient visits with clinical H&N examinations with endoscopy every 3 months in year 1-3 and every 6 months in year 4-5.
- Scheduled imaging: H&N MRI or CT scan with contrast and chest CT scan with contrast at 6 and 18 months after randomization
- Chest CT scan without contrast at 30, 42 and 54 months only in participants with smoking habits.
- Monitoring of H&N REC/SPM symptoms by completing a monthly (p/e) PRO questionnaire with rating scale, but the study team will not call the participant to record the PRO results in the pilot study and no alert will be generated in the main study by the ePRO, and the participating center will not arrange urgent appointment, except in case of self-referral for any reasons.

In addition to these monthly self-monitoring of signs and symptoms with the Symptom Tracker, this questionnaire will also be filled out at each scheduled and non-scheduled visit for both arms.

4 STUDY POPULATION AND STUDY PROCEDURES

4.1 Inclusion and exclusion criteria, justification of study population

4.1.1 Inclusion criteria

1. Histopathologically proven invasive HNSCC of the oral cavity (except lip), oropharynx, hypopharynx or larynx
2. ≥18 years of age
3. In non-surgically treated HNSCC: clinical/radiological stage II-IV (excluding M1) according to the UICC / TNM 8th ed. In surgically treated HNSCC: pathological stage II-IV (excluding M1) according to the UICC / TNM 8th ed.
4. Treatment with curative intent, regardless of treatment modality (mono- or multimodal), and FU planned at the participating study center.
   Remark: Patients with one synchronous HNSCC of the oral cavity, oropharynx, hypopharynx and larynx, all treated with curative intent and all in complete remission are eligible. Synchronous tumor must have a less advanced stage than the index tumor used for stratification or in case of equal stage, the synchronous tumor must be the tumor with the better prognostic. (Rules: Better to worse prognostic: Larynx > Oropharynx > Oral cavity > Hypopharynx.) The modality of the treatment must be the same as for the index tumor or less intense.
5. Radiological confirmation of complete remission of disease and no SPM from the 3rd to 6th month after treatment for all stages (minimal demanded imaging: head and neck (H&N) MRI or H&N CT scan and CT scan covering chest to pelvis (with contrast if not contraindicated); or preferable whole-body ^{18}FDG-PET/CT or PET/MRI for patients with ≥T3 and/or N+).
   Note: Patients with positive or equivocal imaging/clinical findings are allowed if the tumor is ruled out by multidisciplinary tumor board decision (e.g. as a consequence of biopsy and/or multiple imaging).
6. Clinical confirmation of complete remission of disease through H&N examination including endoscopy of the pharynx and larynx at the time of enrolment, that is 6 months (+/- 4 weeks) after the last HNSCC treatment
7. Agreement for long term FU (5 years) and all visits are to be performed at the participating center
8. Written informed consent, signed by the patient and the investigator

4.1.2 Exclusion criteria

1. Initial clinical stage I and/or M1 HNSCC (according to the UICC / TNM 8th ed.)
2. Nasopharyngeal cancer and carcinoma of unknown primary
3. Any other previously treated HNC (including parotid and thyroid gland cancer) except for curatively and adequately treated cutaneous carcinoma in-situ, basal cell carcinoma and locally confined T1 squamous cell carcinoma of the skin without any sign of tumor recurrence at the time of screening
4. Any other synchronous malignancy except for one curatively and adequately treated HNSCC of the oral cavity, oropharynx, hypopharynx and larynx, basal cell carcinoma, locally confined T1 squamous cell carcinoma of the skin, low-risk prostate cancer, carcinoma in-situ of the skin or uterine cervix without any sign of tumor recurrence at the time of screening.
5. Any other metachronous malignancy within the last 5 years except for curatively and adequately treated basal cell carcinoma, locally confined T1 squamous cell carcinoma of the skin, low-risk prostate cancer, carcinoma in-situ of the skin or uterine cervix without any sign of tumor recurrence at the time of screening.
6. Participation in another study entailing regular medical exams by ENT specialists or persons involved in the oncological treatment, or regular imaging

7. Pregnant or breastfeeding women
8. Presence of any conditions that potentially hamper compliance with the study protocol and FU schedule at the participating center

4.2 Recruitment, screening and informed consent procedure

4.2.1 Recruitment

For the information of patients about the trial, a recruitment flyer is developed together with our Bern University Hospital patient advisory board and, upon EC approval, will be distributed to the relevant departments of participating centers.

Pilot phase

The goal is to include ≥16 participants in one year by three Swiss centers. Since feasibility is an important issue in this study, we will evaluate it in terms of recruitment rate within one year and participant's compliance to FU exams for at least one year after randomization.

A participation questionnaire will be completed by all patients of the pilot study who are informed about the study by the physician. The main goal of the questionnaire is to better understand the specific motivation of patients to participate or not in the proposed trial.

Main study

Patients' recruitment will be ensured through the collaboration with several large hospitals in Switzerland and other European countries (34-42 participating centers).

4.2.2 Pre-screening

Each center will pre-screen all clinical stage II-IV (non M1) patients between the 3rd and 6th month after the end of treatment, when the scheduled FU usually starts. Pre-screening is based on data collected as per routine to assess eligibility to participation in this study.

4.2.3 Informed consent procedure

After initial confirmation of fulfilling all eligibility criteria, the investigators will explain to the potential participant the nature of the study, its purpose, the procedures involved, the expected duration, the potential risks and benefits and any discomfort it may entail. In addition, all participants will be informed about:

- The possible adverse events
- The procedures and possible hazards to which the participant will be exposed
- The mechanism of FU strategy allocation
- Strict confidentiality of any participant data
- Medical records possibly being reviewed for trial purposes by authorized individuals other than their treating physician

Each potential participant will be informed that the participation in the study is voluntary and that he or she may withdraw from the study at any time and that withdrawal of consent will not affect his or her subsequent medical assistance and treatment.

All potential participants of the study will be provided with a patient information sheet and informed consent (PISIC) describing the study and providing sufficient information for the participant to decide whether to participate in the study. A recall by phone is foreseen within 1 week after the information, the patient will be asked if she/he would participate in this trial. In case of acceptance, enrolment/randomization visit will be scheduled at 6 months (+/- 4 weeks) after the end of the last

treatment, physical examination will be arranged, and all baseline data will be collected during the visit.

A participation questionnaire will be distributed to all patients who have been informed about the study by the physician in the pilot phase.

The formal consent of a participant, using the approved informed consent form, will be obtained before the participant undergoes any study procedure.

The PISIC will be signed and dated by the investigator or his designee at the same time as the participant signs it during the FU visit with physical examination at 6 months (+/- 4 weeks; in case of rescheduling of visit by the patients, as soon as possible) after the end of treatment. A copy of the signed informed consent will be handed to the participant. The PISIC will be retained as part of the study records. The informed consent process must be documented in the patient file and any discrepancy to the process described in the protocol must be explained.

The PISIC is given as a separate document dated and version controlled. The translated informed consent documents are to be submitted to ethics committees for approval. All the above must be done in accordance with the applicable national legislation and local regulatory requirements.

4.3 Study procedures

4.3.1 Patient registration and randomization procedure

Patient registration and randomization are performed via an internet-based clinical data management system (CDMS) that also hosts the electronic case report forms (eCRFs). The CDMS will be provided by the Clinical Trials Unit, University of Bern (CTU)**.** Patient registration and randomization will only be accepted from member of the study team as authorized by the principal investigator (PI) in the task delegation log.
To access the study CDMS, the study team member needs a username and a password. This can be requested by sending an e-mail with a completed task delegation log and the training log to: **datamanagement@ctu.unibe.ch**.

Participant registration
Prior to registration, the following steps have to be taken:

1. Fill in the patient pre-screening log (used for monitoring potentially eligible patients and will be destroyed after the end of the accrual period).
2. Check the eligibility criteria.
3. Obtain signed and dated written PISIC from the patient prior to any protocol-specific procedure according to ICH/GCP [94] and local guidelines.
4. Fill in the enrolment and identification lists.

Afterwards, the patient can be registered in the study CDMS through the link below:
https://secutrial.insel.ch/DC2

A participant ID will be generated by the CDMS upon patient registration. Baseline data and eligibility criteria can be entered into the system after patient registration.

Randomization

After entering all the baseline information and eligibility criteria, participant will be randomized in the respective section in the CDMS. Participants will be assigned to either the experimental (individualized de-intensified FU) or control (conventional FU) arm in a 1:1 ratio. Stage (according to the UICC / TNM 8th ed. and in case of presence of synchronous tumour, the more advanced stage will be used for randomization) and whether patients underwent uni- or multi-modal treatment are strong prognostic factors. In addition, the trial site appears to be important given the nature of the intervention. These factors will therefore be used for stratification. We are aware that there are additional prognostic factors such as smoking etc. but already the three mentioned factors result in too many strata to use stratified randomization. Therefore, we will use probabilistic minimization for the allocation.

We acknowledge that minimization entails the possibility of foreknowledge and has the potential to undermine concealment [95]. However, no other allocation procedure was deemed preferable when balancing all advantages and disadvantages, especially when considering whether software implementation of other techniques is validated. The minimization algorithm that is implemented in the CDMS is validated. If implementations of other covariate balancing allocation techniques e.g. the Minimal Sufficient Balance method [96] become available before trial start, we will consider them.

Recruiting investigators receive the allocation only after randomizing a participant. Only system administrators who are otherwise not involved in the trial will have access to the algorithm during the enrolment period.

4.3.2 Electronic case report forms completion

Data will be reported on the eCRFs via the CDMS. The list of study members authorized to enter data must be identified on the task delegation log and they must have completed the training of the study and the CDMS. In all cases, it remains the responsibility of the PI to check data that are entered in the database as soon as possible and that the electronic forms are filled out completely and correctly. Central Data Monitor will check the data in the eCRFs and raise queries directly in the CDMS and site can answer and correct the data directly in the eCRFs as applicable. In case, a form has been locked by the Central Data Monitor after review and site wants to change data in these eCRFs, the site must contact the Central Data Monitor to reopen the forms for correction.

All original source data should be kept at the site, including the pPRO that were completed by the patients in the pilot phase. Patients should bring all pPROs back to the site at each scheduled visit or send them to the site by post or email until completion of the study.

4.3.3 Detailed study procedure at each visit

The timing of FU visits and the assessments to be performed for the participants of the control arm (conventional FU) and experimental arm (individualized de-intensified FU) are detailed in Appendix 1: Schedule of assessments, where summaries all study visits, relevant procedures, and exams as well as all timelines are listed. All assessments for secondary endpoints will end at 5 years FU, the date of multidisciplinary tumor board decision for the treatment strategy concerning the first REC/SPM after the primary treatment or at death.

Demographics, height/weight, level of education, and physical exam

Gender, height measure, and level of education are recorded only once at the enrollment visit. Weight and examination including rigid/flexible endoscopy by the physician(s) (ENT specialist, H&N Surgeon) are assessed during the enrolment (range: maximum 4 weeks before enrolment and ideally on the day of enrolment) and during each study-specific visits.

Highest level of education is categorized as follow:

Highest levels of education	Description
No formal education	Preschool or home school
Primary education	Compulsory school only
Secondary education	High school after compulsory school, vocational training
Tertiary education	University or comparable degree including doctorate

Use of electronic devices and internet

The frequency of use of electronic devices (including smartphones and computers) and internet will be recorded during the baseline visit. In case a participant is not comfortable using electronic device or the internet, participants have the opportunity to continue the study using the pPRO instead of the ePRO.

Tumor characteristics (only once at the enrollment/randomization visit)

- Date of diagnosis
- Tumor localization (oral cavity, oropharynx, hypopharynx, larynx)
- HPV status (p16), differentiation
- TNM (according to the UICC / TNM 8th ed.)
  - in non-surgically treated HNSCC: Initial clinical/radiological stage II-IV (excluding M1) of the oral cavity, oropharynx, hypopharynx and larynx, histopathologically confirmed
  - in surgically treated HNSCC: pathological stage II-IV (excluding M1) of the oral cavity, oropharynx, hypopharynx and larynx
- TNM of a synchronous tumor (according to the UICC / TNM 8th ed.)
  - in non-surgically treated HNSCC: Initial clinical/radiological stage II-IV (excluding M1) of the oral cavity, oropharynx, hypopharynx and larynx, histopathologically confirmed
  - in surgically treated HNSCC: pathological stage II-IV (excluding M1) of the oral cavity, oropharynx, hypopharynx and larynx
- Treatment (surgery, radiotherapy, concomitant radio-(immuno-)chemotherapy, neoadjuvant chemotherapy) including start and end date

Tobacco and alcohol consumption

Smoking and excessive alcohol consumption should be strictly discouraged, and participants should be referred to a smoking and/or alcohol cessation professional if required. Past and current smoking habits and status, and alcohol consumption must be recorded during the enrolment and every 6 months.

Smoking habits (cigarettes, cigars, cigarillos, pipe, tobacco, water pipe): 1) never, 2) former smoker, 3) current smoker.

Amount in pack-years: smoking pack-years are calculated using Dr N J Masters and Catherine Tutt smoking pack-year calculator. [97, 98] https://www.smokingpackyears.com/

Alcohol consumption: 1) never, 2) former alcohol drinker, 3) current alcohol drinker

Amount: Frequency of units per week (1 unit = 0.3 dl spirit, 1 dl wine, 3 dl beer), year of alcohol drinking cessation.

All current smokers should be advised to quit smoking, and previous smokers should be advised[i] to remain abstinent from smoking. For additional cessation support, the participating center should recommend the patient to consult Provider Smoking Cessation Resources up to the participating centers' standards. Alcohol counseling should be performed as clinically indicated. All additional visits/cares must be assessed as events under concomitant care.

Performance status assessment
All participants will be assessed with Eastern Cooperative Oncology Group/ World Health Organization (ECOG/WHO) performance scale [99] at the date of enrolment and every 6 months.

ECOG/WHO Performance Status Scale	
Grade	Descriptions
0	Fully active, able to carry on all pre-disease performance without restriction.
1	Restricted in physically strenuous activity but ambulatory and able to carry out work of a light or sedentary nature (e.g., light housework, office work).
2	Ambulatory and capable of all self-care, but unable to carry out any work activities. Up and about more than 50% of waking hours.
3	Capable of only limited self-care, confined in bed or chair more than 50% of waking hours.
4	Completely disabled. Cannot carry on any self-care. Totally confined to bed or chair.
5	Deceased.

Instruction and training on signs and symptoms alarming for REC and SPM

During the baseline and randomization visit, a personal and individual, intensive onsite training of the signs and symptoms alarming for REC and SPM will be performed by head and neck surgeons and/or radiation oncologists. This training also includes the use of the paper or web-based Symptom Tracker (p/ePRO) questionnaire. The understanding and correct use of this tool in the experimental arm is monthly surveyed by the study nurse or the electronic application, warning the patient and the center in case of alarming conditions indicating a possible REC or SPM or in case of non-completing the questionnaire. In case the participants missed to complete 3 p/e PRO questionnaire, a refresher training will be provided through phone call by the study team. This should increase the adherence to the experimental arm strategy. In the standard arm, refresher training on recording/monitoring signs and symptoms in the p/ePRO will be given during the regular scheduled visits if necessary. Study nurses will be trained for the alarming signs and symptoms and the correct completion of the p/ePRO questionnaire. Independent of the training, patients of both arms will be reminded at each scheduled visit to an early self-referral in case of alarming signs and symptoms suspicious for REC/SPM.

Assessment of signs and symptoms on head and neck / chest using the PROs

All participants will be assessed with a PRO predefined signs/symptoms monitoring rating scale at the date of enrolment and during the whole study or until REC/SPM or death. PRO with weight measures and rating scale either in paper-base (pilot phase) or browser-base (main study) has to be filled out monthly and at each scheduled or non-scheduled visit by the participants before the medical consultation with the physician (Appendix 2). This PRO monitoring at each visit serves only to assess the signs and symptoms at these specific moments and are not prone to be compared with the monthly filled out PRO.

In the experimental arm, participants will get a reminder to complete the PRO the day before the expected date to fill out the PRO and up to three reminders in case of missed completion after the expected date in the main study. For the pilot phase, the study team will call to interview for the PRO results on the day after the fixed date of PRO completion. In case the patient did not fill in the PRO, the study team will call up to 2 times within 1 week of the initial call to interview for the results. Depending on the rating, the PRO will trigger an alert (phone call by the study team during the pilot phase; automated electronic alert in the main study) with recommendation for a control earlier than scheduled visit in case of conditions indicating possible REC/SPM. In this case, an ‘open urgent appointment’ will be arranged for the participant by the participating center within 2 weeks.

In the conventional arm, the participant will also complete the PRO monthly and at every visit, but study team will not call the participant and no alert will be sent.

The assessment of fear of recurrence is included in the PROs monitoring and will be assessed also every month using the single-item screening question developed by Rogers et al. (2015) “Fear of the cancer coming back: 0 = I have no fear of recurrence, 1 = I have a little fear, with occasional thoughts but they don’t really bother me, 2 = I am sometimes having fearful thoughts but I can usually manage these, 3 = I get a lot of fears of recurrence and these can really preoccupy my thoughts, 4 = I am fearful all the time that my cancer might return and I struggle with this”. [100] As there is no validated version in German, the original English version was translated and back-translated according to the standards of the European Organization for Research and Treatment of Cancer (EORTC) [101] by a native German speaker and back translated by an independent bilingual native German/English speaker. A third person mediated deviations from the original, and the final version was reached in consensus.

For all participants’ visit due to PRO triggers or self-referral, diagnostic work-up will be done according to the participating centers’ standards for excluding REC/SPM and/or adverse events. All additional visits/cares must be assessed as events under concomitant care.

Head and neck MRI (or CT scan) and CT scan of the trunk

All participants with staged T2 N0 of both arms will be assessed by head and neck MRI (or CT scan) (according to local protocols of participating centers) and CT scan covering chest to pelvis with contrast between the 3rd and 6th month after treatment to confirm complete remission and exclude a SPM before randomization.

- Head and neck MRI: morphological sequences must include T2 weighted sequences as well as T1 weighted sequences with contrast administration (with fat saturation) according to the standards of each center.
- Head and neck CT scan (in case MRI is not available): contrast-enhanced if no contraindication, CT scan according to the standards of each center.
- CT scan covering chest to pelvis: standard contrast-enhanced according to the standards of each center.

The same method of assessment and the same technique should be used in the head and neck region during the study-specific time points at 6 and 18 months (+/- 4 weeks) after the randomization in the participants of the conventional (standard) FU arm. A chest CT scan should be performed with contrast in participant of the conventional (standard) FU arm at 6 and 18 months. In case of contraindication for contrast application, MRI and/or a CT scan without contrast should be performed.

In case of suspicious findings, diagnostic work-up will be done according to the participating centers' standards for excluding persistent/progredient disease/SPM (between the 3rd and 6th month after treatment) and/or REC/SPM (>6 months after treatment).

^{18}F-FDG-PET/CT or -PET/MRI (or head and neck MRI (or CT scan) and CT scan covering chest to pelvis)

All participants staged ≥T3 and/or N+ should be assessed by a whole body ^{18}F-FDG-PET/CT or PET/MRI with additional head and neck acquisitions and contrast-enhanced CT scan of the head and neck region between the 3rd and 6th month after treatment to confirm complete remission and exclude a SPM before randomization. In case of non-availability of PET/CT or PET/MRI, head and neck MRI (or CT scan) and CT scan covering chest to pelvis with contrast according to local protocols of participating centers (see above) will be performed to confirm complete remission and exclude a SPM before randomization. In case of contraindication for contrast application, MRI and/or CT scan without contrast should be performed.

In case of suspicious lesions, diagnostic work-up will be done according to the participating centers' standards for excluding persistent/progredient disease/SPM (between the 3rd and 6th month after treatment).

Low-dose chest CT scan without contrast

Participants of the conventional (standard) FU group with

1) active smoking ≥20 pack-years or

2) smoking habits at least within the last 15 years before cancer diagnosis and ≥20 pack-years

will be assessed by a low-dose chest CT scan without contrast yearly (+/- 4 weeks of the study specific time point, as soon as possible in case of rescheduling by patient) from 18 months to 5 years post-randomization to exclude metastasis/SPM as recommended by the Lung Cancer Screening NCCN Guidelines, Version 1.2022 [25]. Current active smokers or former smokers who smoke less than 20 pack-years do not get regular chest CT. The same method of assessment and the same technique should be used during the whole study and the study-specific time points. In case of suspicious findings, work-up should be performed according to local standard.

Adverse events

Specific treatment-related adverse events (**Appendix 3**) will be assessed and graded using the Common Terminology Criteria for Adverse Events (CTCAE version 5.0) at baseline and every scheduled and non-scheduled visit in both study arms. Assessment of each shortlisted AE including thyroid function should be performed according to local standard and covering the period since the last assessment. All AEs (also those not specifically assessed in the study) should be managed up to the participating centers' standards. All additional visits/cares must be recorded as non-scheduled visit and/or in the concomitant care and cost questionnaire.

Concomitant care questionnaire

Any hereunder mentioned event since the last scheduled visit will be assessed:

- Regular and emergency visits to any physician, hospitalizations and treatments for non-HNC-related reasons
- Additional, non-scheduled visits to physicians, recommendation for a control earlier than scheduled visit triggered by PRO, any patients self-referral visits, imaging, hospitalizations and treatments for cancer-/cancer treatment-related reasons (see **Appendix 3**: Adverse events), dental evaluation and treatment, care for dysphagia, radio necrosis, malnutrition, etc.
- All supportive therapy (including nutrition, antibiotics, analgesics, transfusion of blood products, etc.) for optimal medical care at the discretion of the attending physician(s) within the parameters of the protocol

Health-related quality of life

Health-related QoL is a multidimensional construct, which can be defined as a state of general well-being reflecting physical, psychological, and social well-being and the impact of the disease and/or treatment-related symptoms on daily-life functioning. The patient's subjective perspective is an inherent component of health-related QoL and is, therefore, best assessed via self-administration.

Reducing mortality and morbidity is still the most important factor in cancer clinical research. Nevertheless, issues such as reducing side effects, symptom relief and improving patients' satisfaction have also become relevant parameters in the evaluation of medical strategies. Cancer treatments may produce adverse effects and diminish a patient's QoL even when survival is extended. Progress in the acceptance of new cancer therapies is sometimes critically dependent on their health-related QoL consequences.

In this study, health-related QoL will be assessed using the EORTC core questionnaire EORTC QLQ-C30 and the HNC-specific module EORTC QLQ-HN43 [102, 103]. The QLQ-H&N43 is comprised of 43 items measuring health-related QOL aspects specific for HNC patients.

The assessment of health-related QoL will be performed at baseline and six-monthly in both study arms. Changes in health-related QoL scores over time will be analyzed.

Health-related QoL as measured by EQ-5D(-5L) [104] will be assessed at baseline and then yearly in each study arm. However, this data will only be analyzed as part of the cost effectiveness/utility analyses and not as a separate endpoint.

Number of scheduled and non-scheduled visits, compliance with the symptoms monitoring (p/e)PRO

To evaluate the compliance with the scheduled FU visits/procedures and the recording of the symptoms monitoring by completing the paper or electronic (p/e)PRO questionnaire, any hereunder mentioned event since the last scheduled visit will be assessed:

- Number of occurred regularly scheduled FU visits (physical ENT-examination / imaging)
- Number of visits' rescheduling/recalls for missed scheduled FU visits (rescheduling of visits within +/- 4 weeks of the visit window by the participant does not count as non-compliance)
- Number of any self-referral FU visits
- Number of recommendation for a control earlier than scheduled visit triggered by the symptoms monitoring (p/e)PRO
- Number of FU visits occurred due to recommendations by the symptoms monitoring (p/e)PRO

Health care utilization (cost components)

The following cost components have been identified as being related to head-and-cancer care. As a consequence, all the listed procedures happening over the FU period must be reported in the eCRF to allow calculation of the costs. Cost components can be categorized in FU visits (scheduled/non-scheduled), imaging, complications, diagnostic tests, drugs and out-of-pocket cost. A concomitant care and cost questionnaire was designed to capture all additional care and cost.

FU visits

- Scheduled FU visits
- Non-scheduled additional visits due to self-referral or referral via other physicians at the participating center and outside

Imaging

- Scheduled FU imaging (H&N MRI or CT scan / chest CT scan)
- Non-scheduled imaging (H&N MRI or CT scan / CT scan of the trunk / PET/CT resp. PET/MRI) due to adverse events
- Non-scheduled imaging (H&N MRI or CT scan / CT scan of the trunk / PET/CT resp. PET/MRI) due to suspicion of REC/SPM
- Other imaging performed outside of the participating center

Complications

- Treatment-related procedures
  - Out-patient visits/treatment for adverse events, malnutrition, dental control or complications, physical rehabilitation, psycho-oncological support, speech therapy, and out-patient care
  - Hospitalization/treatment for adverse events, malnutrition, dental control or complications, physical rehabilitation, psycho-oncological support

Diagnostic tests

- Cancer-related procedures
  - Out-patient procedures for suspicion of REC/SPM (biopsy)
  - Hospitalization due to suspicion of REC/SPM (panendoscopy)

Supplements
- Nutritional supplements (e.g. protein shake, multi-vitamin)

Out-of-pocket costs

- Work status
- Number of days lost/unable to work due to treatment-related adverse events
- Number of days lost/unable to work due to procedures for suspicion of REC/SPM

4.3.4 Other evaluation and supportive care

Thyroid hormone evaluation/substitution, dental evaluation/rehabilitation and supportive care and rehabilitation (e.g. speech/hearing and swallowing evaluation and rehabilitation, nutritional evaluation and rehabilitation, depression evaluation and treatment, smoking cessation and

alcohol counseling, lymphedema evaluation and rehabilitation) must be performed as clinically indicated according to the standards of the participating center.

4.3.5 Study procedure outside of the scheduled in-person FU visits

Paper patient-reported outcomes (PRO) - pilot phase

A paper-based PRO questionnaire containing a list of predefined signs/symptoms indicating the need for a control earlier than scheduled visit in conditions indicating possible REC/SPM will be completed by participants monthly (see section 4.3.3 Detailed study procedure at each visit). Study team will call and interview the participant of the experimental arm monthly to collect the results of the PROs questionnaire. In case of non-completion of the questionnaire, the study team will remind the patient a maximum of 3 times to fill out the PROs questionnaire, which should increase the adherence to the experimental arm strategy. After the third recall, a new training for the PROs questionnaire use will be performed by telephone call or a personal visit. The study team will control the records together with the patient. Depending on the conditions, the study team will give recommendation for a control earlier than scheduled visit. In conditions indicating possible REC/SPM, the participating center will organize this visit within 2 weeks.

For participant of the control arm, study team will not call to collect the results and no urgent appointment will be arranged by the study team.

Electronic patient-reported outcomes (ePRO) - main study

A browser-based predefined signs/symptoms monitoring (ePRO with rating scale) will be filled in monthly by participants. An automated alert with recommendation for a control earlier than the scheduled visit in conditions indicating possible REC/SPM will be sent to the participant of the experimental arm and the participating center, which will organize a non-scheduled control within 2 weeks.

For participant of the control arm, no alert will be generated, and the participating center will not arrange urgent appointment.

All participants can contact the participating site for a self-referral, non-scheduled FU visit at any time during the study period. In case of the demand for any self-referral, non-scheduled FU visit, an ‘open urgent appointment’ will be arranged by the participating centers’ team within 2 weeks.

4.4 Procedure in suspicion of recurrence/second primary malignancy

In case of suspicion of REC/SPM (signs/symptoms, imaging, physical examination), diagnostic work-up (additional procedures and imaging) will be done according to the participating centers’ standards. All these procedures have to be assessed and recorded in the eCRF.

4.4.1 Assessments in case of recurrence/second primary malignancy

In case of REC/SPM, diagnostic work-up and treatment will be done according to the participating centers’ multidisciplinary tumor board decision. Participants continue with off-protocol FU according to the center standards. The participants will be monitored until the end of 5 years or death for the primary endpoint. The information will be acquired through participating center’s information system or via direct or indirect communication with the participant, treating physician (e.g. medical oncologist responsible for systemic treatment), family physician or any caregiver.

All following assessments have to be recorded in the eCRF:

- Date of biopsy-proof (Note: In special scenarios such as co-morbidities, patient's rejection or other medical conditions, a biopsy can be omitted if there is a consensus for a REC/SPM at the participating centers' multidisciplinary tumor board based on the clinical and/or radiologic findings)
- Date of the first multidisciplinary tumor board decision for the treatment strategy for REC/SPM
- In case of REC: local, regional, metastatic with localization (brain, lymph nodes other than neck, peritoneum, adrenal glands, skin, lung, liver, bone and others)
- In case of SPM: head and neck localization (oral cavity, nasopharynx, oropharynx, hypopharynx, larynx, nose and paranasal sinuses, major salivary glands, thyroid gland, soft tissue, and bone/cartilage); other localizations (lung, bladder, prostate, stomach, renal, esophagus, and others), histopathology (Solid tumors, Leukemia and Myeloma, Lymphoma, Group solid tumors: squamous cell carcinoma, adenocarcinoma, and others) (if available)
  - TNM of a REC (according to the UICC / TNM 8th ed.): clinical/radiological recurrent TNM (rTNM) and recurrent Staging (rStaging)
  - TNM of a SPM tumor (according to the UICC / TNM 8th ed.): clinical/radiological stage SPM-TNM and SPM-Staging
- Treatment: curatively intended treatment (surgery, radiotherapy, concomitant radio-(immuno-)chemotherapy, neoadjuvant chemotherapy) including start and end date, palliatively intended treatments, best supportive care
- Weight, performance status (ECOG), tobacco and alcohol consumption of the last records
- Signs/symptoms rating of the last record of the p/e PRO monitoring
- Circumstances triggering for diagnosis of REC/SPM (see *Table 6. Categorization of circumstances for diagnosis of recurrence or second primary malignancy.*

Table 6. Categorization of circumstances for diagnosis of recurrence or second primary malignancy.

**Circumstances for diagnosis of recurrence / second primary malignancy**	**Category**
Signs/symptoms remarked by the patient + self-referral before the scheduled visit → diagnosis	A
Signs/symptoms mentioned by the patient at the scheduled visit → diagnosis	B
No signs/symptoms mentioned by the patient at the scheduled visit, but pathological physical findings during examination → diagnosis	C
Signs/symptoms remarked by the patient + suspicion of REC/SPM in the systematic imaging → diagnosis	D
No signs/symptoms + suspicion of REC/SPM in the systematic imaging → diagnosis	E
PRO triggered recommendation for non-scheduled visit → diagnosis	F

4.4.2 Procedure in case of death

In case of death, the following assessments have to be recorded in the eCRF:

- Date of death
- Cause of death: primary cancer related death, second primary malignancy related death, death related to treatment complication (at the discretion of the local PI) and other causes

4.5 Follow-up after 5 years

Follow-up after the period of the trial will be performed according to the participating centers' standards.

4.6 Strategies for increased retention and patient involvement

4.6.1 Engaging and sensibilize the participant

To increase the compliance/adherence, the schedule for the assigned FU integrated in a standardized alarm flyer will be distributed with space to insert the next appointment. We will use different colors for chronological progression, emphasizing the positive implications associated with the time passed after the treatment. Engaging the patients in their own care and rehabilitation is consistent with a fundamental feature of all enhanced recovery programs. On the alarm flyer, information will be provided to remind the participant of persisting/worsening signs and symptoms (red flags) suspicious for REC/SPM. The importance of following strictly the FU schedules and early self-referral in case of alarming signs and symptoms is highlighted on the flyer. This flyer also includes the contact information of the participating sites' unit undergoing the FU to facilitate fast self-referral and review if required.

Participants of both arms receive intensive sensitization/training to recognize signs and symptoms of REC/SPM and treatment-related adverse events (standardized alarm flyer). A browser-based predefined signs/symptoms monitoring (ePRO with rating scale) is to be filled in monthly (or paper-based in the pilot phase) by participants. According to a set of pre-defined conditions indicating possible REC/SPM, an automated alert with recommendation for a control earlier than scheduled visit will be sent to the participant of the experimental arm and the participating center. Systematic reminders generated will increase and assure compliance. During the pilot phase, the study team will contact the participants by phone one day after each expected recording, control the completion of the questionnaire, eventually remind them to complete the questionnaire and recommend for a control earlier than the scheduled visit depending on the ratings/conditions. In conditions indicating possible REC/SPM, the participating center will then organize this control within the next 2 weeks.

4.6.2 Recall

Participants with no-show at the scheduled visit will be contacted by phone or mail for rescheduling a new appointment. Rescheduling will be performed three times after each missed visit before declaring as "discontinuation of FU scheme" in the eCRF (see section 4.7), as long as the primary endpoint information can still be retrieved from the family physician at the end of the 5-year FU. If the survival status at 5 years cannot be retrieved, this is recorded as "Lost to FU".

The compliance/adherence with the study will be assured via regular central data monitoring and on-site monitoring, which will be coordinated by the CTU, University of Bern, Switzerland. Each participating center is responsible for assuring participants' compliance/adherence and is responsible for developing and implementing institutional standards to avoid a rate over 5% of lost-to-FU.

Participants' retention can be improved by a high-quality consenting procedure. Special care will be given to the design of the PISIC form, and to how the study personnel communicate the study design to the eligible patients.

4.7 Discontinuation

Participants may discontinue from the randomized follow-up scheme at any time at the discretion of the investigator due to:

- REC/SPM
- Serious adverse event (SAE)
- Based on any other relevant medical condition, e.g. comorbidity
- Pregnancy
- Discontinuation after 3 recalls
- Participant's wish

If participants discontinue from the trial FU scheme but did not withdraw consent from the trial, the participant remains in the trial. Participants who wish to discontinue from the trial follow up scheme will be recommended to follow the participating centers' standard FU scheme.

We collect FU visits data available per routine (including date of follow up, physical examination, imaging and diagnostic performed and their results), as well as oncological outcome and survival status at 5 years. The information will be acquired through the participating center's information system or via direct or indirect communication with the participant, participant's family members, treating physician (e.g. medical oncologist), family physician or any caregiver.

For participants discontinuing or deviating from the assigned FU schedule in case of REC/SPM, symptom recording via the PRO will be disabled and no routine FU data as per protocol will be collected. The following outcome data should be collected:

- Date of biopsy-proof (Note: In special scenarios such as co-morbidities, patient's rejection or other medical conditions, a biopsy can be omitted if there is a consensus for a REC/SPM at the participating centers' multidisciplinary tumor board based on the clinical and/or radiologic findings)
- Date of the first multidisciplinary tumor board decision for the treatment strategy for REC/SPM
- In case of REC: local, regional, metastatic with localization (lung, liver, bone, and others)
- In case of SPM: head and neck localization (oral cavity, nasopharynx, oropharynx, hypopharynx, larynx, nose and paranasal sinuses, major salivary glands, thyroid gland, soft tissue, bone/cartilage); other localizations (Lung, bladder, stomach, renal, esophagus, others), histopathology (if available)
  - TNM of a REC (according to the UICC / TNM 8th ed.): clinical/radiological rTNM and rStaging
  - TNM of a SPM tumor (according to the UICC / TNM 8th ed.): clinical/radiological stage SPM-TNM and SPM-Staging
- Treatment: curatively intended treatment, palliatively intended treatments, best supportive care
- Date of treatment start

4.8 Withdrawal

Participants have the right to withdraw consent and refuse further participation for any reason and at any time. Participants who wish to withdraw from the trial will be recommended to follow the participating centers' standard FU scheme.

For the participant's security, a last examination should be performed and a further FU according to the participating centers' standard. Data collected up to the time point of withdrawal and, if performed, the above-mentioned final visit will be used for analysis. It is not possible to anonymize the patient data upon withdrawal, therefore, the data will continue to be coded after withdrawal.

Participant will be asked if data collected as per clinical routine (result of the physical examination with fiber- or rigid endoscopy, imaging and diagnostic that has been performed and their results, and oncological outcome) and survival status can still be collected for the study. If participant agrees, he/she will sign another informed consent to allow further collection of his/her data for the trial.

4.9 End of study

Study team shall complete the end of study form in the study database when the participant has completed 5 years of follow-up, death or withdrawal of consent and the additional consent for further collection of data until the end of study at 5 years was not signed. Discontinuation or withdrawal with further consent on data collected as per clinical routine do not consider as "end of study". Participant is considered as completed the study according to protocol until the end of the 5 years follow-up if he/she is alive without discontinuation or withdrawal or lost to follow-up or withdrawal of consent at the end of the 5-year follow-up.

5 STATISTICS AND METHODOLOGY

5.1 Statistical analysis plan and sample size calculation

The statistical analysis of the trial will be done at CTU, University of Bern based on well-established standard operating procedures. This process calls for a statistical analysis plan to be written at the start of enrolment and to be finalized before 25% of participants are enrolled. The plan will determine all necessary data preparation steps (e.g. additional validations, generation of new variables), definitions (e.g., analysis sets), and statistical analyses (e.g., models, outputs such as tables and graphs).

5.1.1 Justification of the sample size

Sample size calculation is based on the primary endpoint of death from any cause, the effect measure of 5-year restricted mean survival time, and the dual-criterion design as described in section 3. The sample size calculation does not take into account the interim analysis (at a minimal FU of 20 months) as this is designed as non-binding and therefore not formally influencing the type I (or II) error of the trial. Sample size calculation is based on simulations (10,000 replications for each scenario) where we also investigated the operating characteristics of the trial. Calculations were done using R and Stata.
We used internal data of the Department of Oto-Rhino-Laryngology, Head and Neck Surgery, Inselspital, Bern University Hospital to inform OS in the control group. This is high-quality data prospectively collected from 2016-2019. Only eligible patients to the proposed trial are considered (N=179 of which 35 died) with a median follow-up of 2.9 years. The data could be well approximated by a Weibull distribution with shape parameter a=1.18 and scale parameter b=10.25. This corresponds to a 5-year restricted mean survival time of 4.65 years.
The graph below shows the data used and the fitted Weibull model:

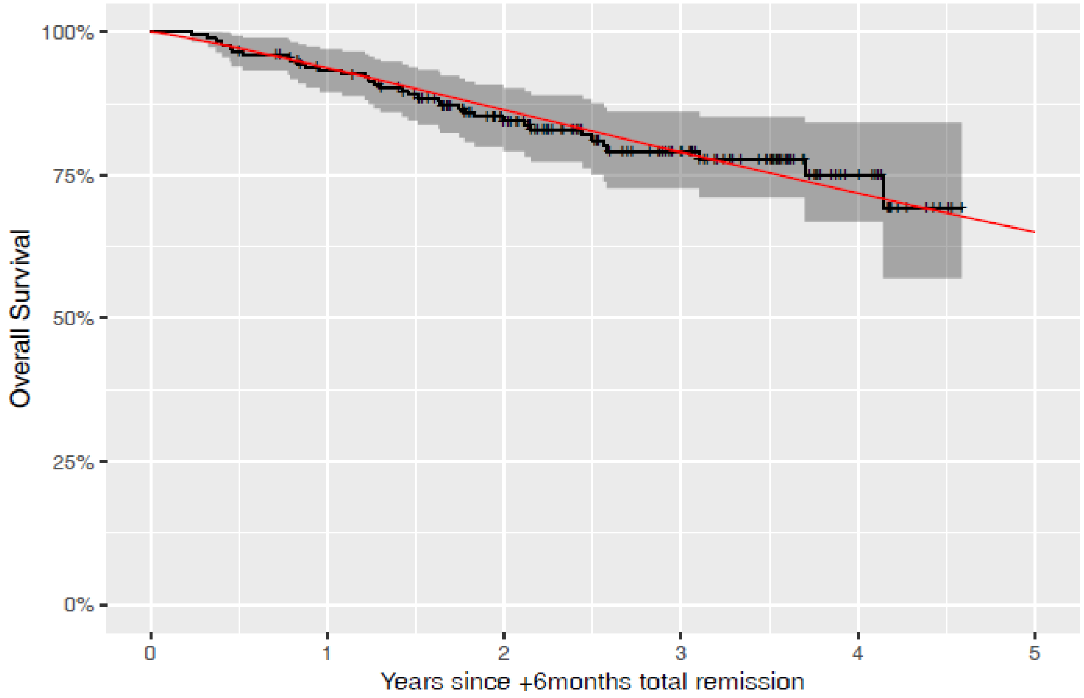

In addition, the following assumption were used:

- Enrolment period: 36 months (uniform)
- Allocation ratio: 1 to 1
- Follow-up per participant: 5 years
- Losses to follow-up for the primary endpoint will be minimal. As long as a participant does

not withdraw consent, the participant, family doctor, treating physician and/or relatives can be contacted to collect the survival status. Therefore, we considered 5% cumulative attrition at 5 years.

- Difference between the two trial groups in terms of OS: none
- Non-inferiority criteria:
  - Decision value: 2 months
  - Non-inferiority margin: 5 months
- One-sided alpha: 0.025
- Power: ≥90%

Using these assumptions, we calculated a required sample size of 525 participants overall. To account for uncertainties in the assumptions, we fixed the final sample size at 550 participants overall.

5.1.2 Statistical analysis of the internal pilot phase

Data for endpoints of the internal pilot phase (see section 3.2.2) will be analyzed descriptively. Endpoint data for the main trial that is collected during the internal pilot phase will only be used for the main trial and not analyzed at the end of the internal pilot phase unless the trial will not progress to the main trial.
The data from the participation questionnaire will be analyzed descriptively plus qualitatively (free text responses). The number of completed surveys is too small to allow for generalizable conclusions. Rather, the intention of the questionnaire is to complement other experiences acquired during the pilot to potentially modify the definite main trial, especially with respect to enrolment strategy and patient information approaches.

5.1.3 Statistical analysis of the main trial

5.1.3.1 Datasets to be analyzed, analysis populations

In line with the estimands defined in section 3.3, we define three different analysis sets for this trial of a sustained intervention: 1) treatment policy or intention-to-treat; 2) while-on-treatment (per-protocol 1); 3) hypothetical (per-protocol 2). The intention-to-treat analysis set consists of all randomized participants in the allocated group regardless of any protocol violations. For the per-protocol analysis sets, we will first (before the main trial starts to allow for an appropriate data collection) carefully define criteria for adherence to the respective follow-up scheme and reasons for non-adherence. Two per-protocol sets will be derived: 1) we will classify participants as non-adherent only if non-adherence is not medically indicated (while-on-treatment) and 2) we consider any non-adherence regardless of the reason (hypothetical). The hypothetical analysis set will only be used for exploratory analyses.
Adherence is defined as the number of completed scheduled visits/exams out of all scheduled visits/exams (clinical and radiological); (re)scheduling of visits by the participants within +/- 4 weeks does not count as non-compliance. The number of recalls will be recorded. Adherence will not be studied after the occurrence of a REC/SPM i.e. participants will be classified as adherent/non-adherent only based on the time before REC/SPM.

5.1.3.2 Primary endpoint

As primary effect measure, the difference in the 5-year RMST will be derived from a flexible parametric survival model adjusted for stratification factors used at randomization [105, 106]. It will be accompanied by a one-sided 97.5% confidence interval to test for non-inferiority of the experimental follow-up scheme. Two analyses sets will be used for the primary analysis as described in the previous section and in section 3.3: treatment-policy (intention-to-treat) and while-on-treatment (per-protocol). The while-on-treatment estimand is prone to selection bias and post-randomization confounding. Participant will be considered censored at the first time interval data indicates non-adherence according to the pre-specified definition of non-adherence not

medically indicated. Inverse probability of censoring weights will be used to recreate an unbiased scenario [107]. Weights will be derived by regression modelling taking into account baseline and time-dependent prognostic factors for non-adherence and the endpoint. Non-inferiority will be declared if both of the following criteria are met in both analyses sets: 1) the point estimate is not larger than the decision value of 2 months and 2) the confidence interval does not cross the non-inferiority margin of 5 months.

5.1.3.3 Other endpoints

No formal testing will be done for other endpoints but the primary. Instead, effect measures will be accompanied by 95% confidence intervals. Analyses of other endpoints will be done using the intention-to-treat analysis set only (section 3.3). Other time-to-event endpoints will be analyzed with the same method as the primary endpoint. Some of these entail competing events e.g., cancer death. The Royston-Parmar flexible parametric survival model allows calculating restricted mean survival time while taking competing risks into account [108]. Continuous endpoints measured longitudinally e.g., health-related QoL or HNC-specific healthcare utilization costs, will be analyzed using a mixed-effects model adjusted for stratification factors used at randomization. We will also model trends over time by introducing treatment-by-time interactions in the model. Because continuous outcomes over time cannot be meaningfully analyzed without taking censoring events e.g., death into account, we will additionally analyze them using a joint model [109, 110]. Count outcomes (number of in-person visits) will be analysed using a negative binomial model adjusted for stratification factors. See below for analysis of costs and related analysis.

5.1.3.4 Additional analysis

In addition to the primary analysis that adjusts for stratification factors, we will also present unadjusted estimates and estimates adjusted for additional covariates (beyond stratification factors).

We will explore the heterogeneity of the treatment effect of the primary endpoint by stratified analyses introducing an interaction term in the model. Subgroup-specific estimates will be accompanied by p-values for interaction. These analyses will only be done in the intention-to-treat analysis set. The following subgroups will be explored: tumor stage, HPV status, tumor site, primary treatment modality.

Finally, we will also provide the hypothetical estimand for the primary endpoint but consider this exploratory.

5.1.3.5 Interim analyses

There will be two formalized interim analyses in the main trial: 1) one to ensure the safety of participants regarding detection of SPM/metastases of the lung and 2) one to continue the trial beyond the foreseen funding period of five years after the start of the main trial (see section 10.1). An independent Data Monitoring Committee (DMC) will evaluate unblinded data of the interim analyses and provide recommendations. Based on their expertise, the DMC may provide recommendations to continue the study unchanged or modify or stop the study after reviewing the result of the interim analyses. The Sponsor will decide on the appropriate action to take and request approval from the EC if any substantial changes to the study are to be implemented. Details of the DMC procedure is available in a separate DMC Charter.

The first interim analysis will be done after the first 100 participants in each group were followed for at least 18 months after inclusion (two years after the end of treatment). The safety parameter is isolated SPM or metastases of the lung. If at the interim analysis, at least seven (7) more participants in the individualized de-intensified FU group have isolated SPM or metastases of the

lung as compared to the conventional FU group, the DMC might recommend amending the protocol by adding chest CT scan assessment for the individualized de-intensified FU group at 6 and 18 months (1 and 2 years after the end of treatment) and once per year in participants with smoking habits similar to the conventional (standard) FU group.

Justification for the (non-binding) threshold: this interim analysis takes a pragmatic approach to ensure safety while allowing the main study question to be answered. An incidence proportion of 5% isolated second primary malignancy or metastases of the lung is assumed in the control group. Based on the simulation, the table below provides proportions of trials that would be modified under various scenarios. A maximum of 5% trials would be modified under reasonable assumptions for this incidence proportion (3-8%) and actually no difference between the arms. In contrast, under the assumption that the experimental follow-up group actually has an increased risk, the chosen threshold will lead to modifications in more than 50% of trials assuming a relative risk increase of 150% and an incidence proportion of 5% in the control group.

**Incidence proportion in control group**	**Actual risk increase in experimental group**	**Proportion of modified trials (rounded)**
0.03	0% (Relative Risk of 1.0)	<0.01
0.03	100% (RR 2.0)	0.10
0.03	150% (RR 2.5)	0.25
0.03	200% (RR 3.0)	0.45
0.05	0% (RR 1.0)	0.03
0.05	100% (RR 2.0)	0.35
0.05	150% (RR 2.5)	0.60
0.05	200% (RR 3.0)	0.80
0.08	0% (RR 1.0)	0.05
0.08	100% (RR 2.0)	0.65
0.08	150% (RR 2.5)	0.85
0.08	200% (RR 3.0)	0.98

After a minimal FU of 20 months of the last enrolled participant, we will perform an interim safety analysis to ensure that the de-intensified arm is not unsafe i.e. overly inferior. We will compare both arms using the difference in 2-year RMST. No formal and binding statistical testing will be done as we cannot use the same effect measure that is used for the final analysis (and no data is available on their correlation). We will either present the difference in 2-year RMST accompanied by confidence levels [111] or by providing posterior probabilities from a Bayesian analysis [112] for different thresholds ('safety margins'). Decision about the final approach will be made by the Steering Committee with the involvement of the independent DMC.

5.1.3.6 Costs and cost-utility analysis

It should be noted that the following analyses will only be done in case the trial shows promising results i.e., non-inferiority is shown. Healthcare costs will be quantified using observed HNC-specific healthcare utilizations multiplied by standardized/average unit costs (for the respective country) and summed per participants from enrolment/randomization up to REC/SPM detection or death.

Cost-utility will be analyzed using the incremental cost-utility ratio as defined by the following formula:

$$\frac{\text{(cost of de-intensified FU – cost of standard FU)}}{\text{(utility of de-intensified FU – utility of standard FU)}}$$

In this study, utility will be expressed as quality-adjusted life years (QALYs). QALYs are calculated by multiplying life years by utility coefficients which are weights, ranging from 0 to 100, where 100

is "perfect health" and 0 corresponds to "worst possible health". The utility coefficients will be measured using the EQ-5D(-5L) [104] completed at baseline and once per year after randomization (see section 4.3.3) which is a standardized instrument for measuring generic health status. Willingness-to-pay thresholds that are considered acceptable by the World Health Organization range from 30,000 to 90,000 USD per QALY gained.

Note: cost effectiveness analysis, where life-years saved are considered in the denominator (instead of quality-adjusted life-years), will not be done.

5.1.3.7 Deviations from the original statistical plan

Any deviation from the original planned analyses will be described and justified in the statistical analysis plan and/or in the final report, as appropriate.

5.2 Handling of missing data and drop-outs

5.2.1 Missing data

In contrast to “discontinuation of the assigned FU scheme” where primary endpoint information could be retrieved (see section 4.7), “lost to FU” is defined as information of the primary endpoint of a patient, which cannot be retrieved anyhow because participant cannot be contacted anymore. In case of lost to FU, patient will be censored at the last time point known to be alive. If cause of death is missing, we will consider the death as endpoint event for the respective (secondary) endpoint (worst case scenario). In a sensitivity analysis, we will run a best-case scenario analysis assuming that the death is not an endpoint event.

Missing data for continuous endpoints will be considered as missing at random. Repeated-measures mixed-effects models implicitly account for missing data if at least one assessment at one time point is available. We will explore robustness of results by sensitivity analyses using single imputation techniques within best- and worst-case scenarios. Multiple imputation is not foreseen.

5.2.2 Drop-outs

We do not expect more than 5% drop-outs for the primary endpoint in this population. Drop-outs/losses to FU will be handled as described above (see section 5.2.1).

6 REGULATORY ASPECTS AND SAFETY

6.1 Local regulations / Declaration of Helsinki

This study is conducted in compliance with the protocol, the current version of the Declaration of Helsinki, [113] the ICH-GCP [94], the HRA [114] as well as other locally relevant legal and regulatory requirements.

6.2 (Serious) adverse events and notification of safety and protective measures

Study duration encompassed the time from when the participant signs the informed consent until the last protocol-specific procedure has been completed. The collection period will start after registration. Ongoing events with onset before the start of treatment will be considered as baseline and will be used as a reference to interpret adverse event occurring during the FU period.

An Adverse Event (AE) is any untoward medical occurrence in a patient or a clinical investigation subject which does not necessarily have a causal relationship with the trial procedure. An AE can therefore be any unfavorable or unintended finding, symptom, or disease temporary associated with a trial procedure, whether or not related to it.

A Serious Adverse Event (SAE) (ClinO, Art. 63) [115] is any untoward medical occurrence that

- results in death or is life-threatening,
- requires in-patient hospitalization or prolongation of existing hospitalization,
- results in persistent or significant disability or incapacity, or
- causes a congenital anomaly or birth defect

Both Investigator and sponsor make a causality assessment of the event to the intervention, (see table below based on the terms given in ICH E2A guidelines) [116]. Any event assessed as possibly, probably, or definitely related is classified as related to the trial intervention.

Relationship	Description
Definitely	Temporal relationship<br>Recurrence after rechallenge<br>(or other proof of drug cause)
Probably	Temporal relationship<br>No other cause evident
Possibly	Temporal relationship<br>Other cause possible
Unlikely	Any assessable reaction that does not fulfil the above conditions
Not related	Causal relationship can be ruled out

- Both Investigator and sponsor make a severity assessment of the event using the CTCAE, version 5.0.
- **Grade 1** Mild; asymptomatic or mild symptoms; clinical or diagnostic observations only; intervention not indicated.
- **Grade 2** Moderate; minimal, local or noninvasive intervention indicated; limiting age-appropriate instrumental activities of daily living (ADL)*.
- **Grade 3** Severe or medically significant but not immediately life-threatening; hospitalization or prolongation of hospitalization indicated; disabling; limiting self-care ADL**.

- **Grade 4** Life-threatening consequences; urgent intervention indicated.
- **Grade 5** Death related to AE.

*Instrumental ADL refer to preparing meals, shopping for groceries or clothes, using the telephone, managing money, etc.

**Self-care ADL refer to bathing, dressing and undressing, feeding self, using the toilet, taking medications, and not bedridden.

6.2.1 Anticipated (S)AE in this study

The following SAEs are either a clear result of the underlying disease therefore it can be excluded that the events were attributable to the intervention under investigation, or are well-known preconditions, these SAEs are thus exempted from reporting to the sponsor and the EC:

- Cancer-/cancer-treatment related imaging procedures
- Cancer-/cancer-treatment related physical examinations
- Cancer-/cancer-treatment related invasive diagnostic procedures (i.e. biopsies, fine needle aspiration, panendoscopy)

Examples of anticipated (S)AE in this study

Related to biopsy, namely (non-exhaustive list):<br>• Hematoma<br>• Bleeding<br>• Feeling unwell, vasovagal syncope<br>• Infection (abscess, septicemia…)<br><br>Related to rigid/flexible endoscopy:<br>• Sneezing and clear discharge from the nose disappearing after examination<br>• Slight pain when passing the rigid/flexible endoscope<br><br>Related to scheduled MRI/CT scan and other imaging (PET/CT) in diagnostic work-up for suspicion of REC/SPM:<br>• Allergic reaction to contrast product<br>• Renal insufficiency<br><br>Related to further investigations in case of suspicion of REC/SPM (ex. panendoscopy):<br>• Pain<br>• Bleeding<br>• Infection<br>• Tear of the tissue<br>• Allergic reaction to anesthetic or sedative

In addition, the following situations are not reportable SAEs:

- Elective hospitalization for pre-existing conditions that have not been exacerbated by trial treatment.
- Hospitalization which was planned before the patient consented for study participation and admission did not take longer than anticipated.
- Hospitalization planned for protocol related treatment or protocol related procedure as per institutional standard timelines.
- Social and/or convenience admission to a hospital.
- Medical or surgical procedure (e.g. endoscopy, appendectomy); the condition that leads to the procedure is an SAE.

- Situations where an untoward medical occurrence did not occur (palliative care, rehabilitation, overdose without occurrence of an AE).
- Anticipated day-to-day fluctuations of pre-existing conditions
- SAEs in participants who discontinued in the study or withdraw his/her consent

6.2.2 Reporting of (serious) adverse events

Only treatment related AEs shortlisted in **Appendix 3** will be documented in the eCRF during FU visit, no other AE will be recorded in the eCRF or reported to the sponsor.

All SAEs that are not exempted for reporting to the Sponsor as defined in section 6.2 are documented and reported to the sponsor. SAE reporting to the sponsor is done via the study CDMS which generates an automatic email notification to the sponsor once a reportable SAE is entered and saved in the system. The sponsor will re-evaluate the reportable SAE and send a confirmation of receipt to the reporting site via email.

Reportable SAEs are collected, fully investigated and documented in source documents and eCRFs. Hospitalization in reportable SAEs is defined as any unplanned, formal in-patient admission, even if the hospitalization is a precautionary measure for continued observation.

The only study specific procedure in this study is the signs and symptoms monitoring via paper or electronic PRO questionnaire, any other interventions to be proceeded on the patients are standard of care. It is not foreseen that any SAE could be attributable to the intervention under investigation, but is a clear result of the underlying disease or the related treatment or diagnosis procedure. If it cannot be excluded that the SAE occurring in Switzerland is attributable to the intervention under investigation, the Sponsor reports it to the concerned EC via BASEC within 15 calendar days and within 7 calendar days in the case of a fatal SAE. SAEs will be reported to all EC involved in the trial according to local regulations and through established reporting channels. Reporting will be done by the Sponsor when this is authorized, and via the PI at the local sites if this is compulsory.

Follow-up of (Serious) Adverse Events

Participants with any reported ongoing SAE at the last scheduled study contact will be followed until resolution of the event or a stabilized condition of the subject has been achieved or until the subject is lost to FU or end of FU.

Notification of safety and protective measures (see ClinO, Art 62, b) [115]

If immediate safety and protective measures have to be taken during the conduct of the study, the sponsor notifies the EC of these measures, and the circumstances necessitating them, within 7 calendar days or according to local regulations. The sponsor must immediately inform all participating Investigators about all the safety and protective measures.

6.3 (Periodic) safety reporting

An annual safety report (ASR/DSUR) is submitted once a year to the local EC by the Investigator (ClinO, Art. 43 Abs). [115]

In this international multicentric study, the ASR/DSUR contains information from all sites including information from sites of all participating countries. The sponsor distributes the ASR/DSUR to all the participating Investigators.

6.4 Pregnancy

Pregnancy is an exclusion criterion for the study. Pregnancy occurring during study participation in this trial, must be reported as discontinuation in the CDMS. Participant's continuation in the study will follows as in section 4.7 Discontinuation.

6.5 Amendments

The sponsor will decide on protocol amendments. Any investigator, DMC, or competent EC may provide suggestions for a protocol amendment. Important protocol modifications will be communicated by the sponsor to the relevant parties (Principal investigators (PIs), competent EC, trial registries and the funding agency as applicable). PIs are responsible for the communication of relevant modifications to the study participants at their respective sites.

Amendments are submitted as required per local law and regulations and will be implemented only after approval of the competent EC. In Switzerland, any substantial amendments will be submitted to the competent EC for approval before implementation (Art. 29 ClinO).

Under emergency circumstances, deviations from the protocol to protect the rights, safety and well-being of human subjects may proceed without prior approval of the Sponsor or the competent EC. Such deviations must be documented and reported by the investigator to the Sponsor within 24 hours and by the Sponsor to the competent EC within the applicable timelines.

A list of all non-substantial amendments will be submitted once a year to the competent EC together with the ASR.

6.6 Notification and reporting upon completion, discontinuation or interruption of the study

6.6.1 Overall study discontinuation

Upon regular study completion, the EC is notified via BASEC within 90 days (ClinO, Art. 38).

Coordinating Investigator and any other competent authority may terminate the study prematurely according to certain circumstances, e.g:

- Ethical concerns
- Insufficient participant recruitment
- When the safety of the participants is doubtful or at risk (e.g. when the benefit-risk assessment is no longer positive)
- Alterations in accepted clinical practice that make the continuation of the study unwise, or
- Early evidence of harm or benefit of the experimental intervention

The DMC might recommend the steering team to stop the trial if the treatment shows harm according to the procedure laid down in the DMC Charter. The Coordinating investigator will

decide on the appropriate action to take based on the recommendation from the DMC and submit to the competent EC for approval before implementation in case of substantial amendment to the protocol.

Upon premature study termination or study interruption, the EC is notified via BASEC within 15 days (ClinO, Art. 38). A final report is submitted to the EC via BASEC within a year after completion or discontinuation of the study (ClinO, Art. 38).

6.6.2 Site or investigator discontinuation

The Sponsor can replace a site at any time. Reasons for replacing a site may include, but are not limited to, the following:

- Slow recruitment
- Poor protocol adherence / Serious breach* of the protocol
- Inaccurate or incomplete data recording
- Non-compliance with the International Council on Harmonisation (ICH) guideline for Good Clinical Practice

The Sponsor can temporarily or permanently discontinue an investigator from participation in the clinical trial at any time. Reasons may include, but are not limited to, the following:

- Poor protocol adherence / Serious breach* of the protocol
- Major deviation** to the protocol
- Non-compliance with the International Conference on Harmonisation (ICH) guideline for Good Clinical Practice

*Serious breach is defined as any conditions, practices or processes that adversely affect the rights, safety or wellbeing of the subjects and/or the quality and integrity of data.

**Major deviation is defined as any conditions, practices or processes that might adversely affect the rights, safety or wellbeing of the subjects and/or the quality and integrity of data.

6.7 Insurance

In Switzerland, the Inselspital, Bern University Hospital, Bern will indemnify participants for any damage they may suffer due to participation in this trial. For this purpose, Inselspital, Bern University Hospital, Bern has taken out insurance for clinical trials with insurance company Zurich Versicherungs-Gesellschaft AG.

For sites outside of Switzerland, the sponsor will provide insurance that covers the requirement of the country of the sites as requested by the concerning EC.

7 FURTHER ASPECTS

7.1 Overall ethical considerations

Given the lack of prospective studies and evidence-based data, there is a pressing need for a representative randomized trial to address the following primary issues:

1. The impact of differently scheduled FU visits with/without routine imaging on OS
2. Oncological outcome and detection of REC/SPM
3. The role of patient-reported signs/symptoms in predicting REC/SPM (self-awareness → self-referral)
4. The role of patients' demographics and tumor characteristics (age, education level, tumor localization, staging, human papillomavirus status, etc.) in the surveillance
5. The impact of two different FU schemes on patients' QoL
6. The financial burden and health costs (direct and indirect) for the patients and the entire healthcare system of two different FU schemes

The results will have a major impact on the development of evidence-based future guidelines with the final goal of developing possible personalized surveillance plans depending different variables, like tumor localizations, stages, other prognostic factors (aggressivity, smoking/alcohol consumption, human papillomavirus, etc.), patients characteristics (age, education level, performance status, comorbidities, etc.) and treatment modalities, and are thus of high relevance for patients, medical staff, and healthcare. The pilot phase of this study will evaluate the feasibility of randomizing patients with complete remission 3-6 months after treatment of HNSCC to a de-intensified and conventional (standard) intensive FU in regard to recruitment rate and adherence to the randomized FU scheme.

There will be only adult participants, but no vulnerable participants included in this study. Participation in this project is of one's own free and participants have the right to withdraw from the study at any point of time. Participants will be informed on any new information about the study throughout the project. The participant data collected in the database will be coded and the code list will be kept at the study site with access to authorized persons only.

For patients

This potentially practice-changing trial will provide high-quality data that will help to develop safe and efficient FU protocols that avoid unnecessary visits (clinical exams and imaging) and their immanent risks. The trial will also allow assessment of the impact of the FU exams on QoL, an important issue that current guidelines fail to account for.

For the medical community

The results of the trial may help medical staff involved in the post-treatment management of HNC patients to guide and justify their decisions based on high-quality scientific evidence, which is not currently available. They may help to design new evidence-based international guidelines.

For healthcare in general

The results of this trial will elucidate the value of different medical procedures of HNC FU. If the trial succeeds in proving the non-inferiority of the individualized de-intensified FU, it may contribute to efforts improving cost-efficiency of healthcare.

7.2 Risk-benefit assessment

Both FU modalities under investigation are associated with distinct benefits and minimal but potential risks.

Table 7. Risk and benefits assessment

	Individualized de-intensified FU	Conventional (standard) FU
**Benefits**	Increased individualized involvement during the FU, monthly surveillance of the patient-reported outcome questionnaire with trigger of alerts for a control earlier than scheduled visits depending on the ratings of signs and symptoms indicating possible REC/SPM followed by procedures to exclude REC/SPM.<br><br>Possible early detection of REC/SPM caused by the more intensive surveillance of pathological signs and symptoms. Less frequent FU visits, no additional x-ray harm due to the absence of systematic radiological imaging, decreased stress due to decreased frequency of FU visits, decreased individual costs for transport/insurance.	More frequent FU visits, systematic radiological imaging confirming objectively and systematically disease-freeness (after 12 and 24 months and then yearly in heavy smokers), possible early detection of REC/SPM caused by more frequent FU visits and systematic imaging.
**Risks**	Possible late detection of REC/SPM due to less frequent FU visits or the absence of systematically planned imaging, increased stress caused by the absence of systematic radiological imaging confirming objectively disease-freeness.	Possible late detection of REC/SPM due to the absence of increased individualized involvement and no systematic surveillance of the patient-reported outcome questionnaire and no trigger of alerts for a control earlier than scheduled visits.<br><br>Harm due to systematic radiological imaging, increased stress due to the frequent FU visits, increased individual costs for transport/insurance, radiological detection of lesions (also of benign nature) leading to further possible harmful investigations/interventions to exclude REC/SPM.

8 QUALITY CONTROL AND DATA PROTECTION

8.1 Quality measures

Study center adherence to the study protocol will be monitored regularly. Protocol deviations will be reported by study centers using the protocol deviation form in the study CDMS. The form will be filed in the investigator site file. Sponsor will discuss the protocol deviation with the study centers by telephone or by written notes as soon as possible.

In addition, on-site and central data monitoring will also monitor adherence. Trial sites will be regularly visited by monitors of the **CTU, University of Bern, Switzerland**. Details on the frequency and contents of the monitoring is detailed in the monitoring plan. Visits will focus on controlling regulatory compliance, monitoring of processes, and verification of source data collection. The central data monitoring encompasses monitoring of data entry progress and procedures for data collection. Any change to the data will be traceable through an audit trail.

Authorities have the right to perform inspections, and Inselspital, Bern University Hospital has the right to perform on-site auditing upon reasonable prior notice. The auditor/inspector must have access to all medical records, the investigator's trial-related files and correspondence, and the informed consent documentation that is relevant for this clinical trial.

PIs will allow the persons responsible for the monitoring, audit or the inspection to have access to the source data/documents and PIs will answer any questions arising. All involved parties will keep the participant's data strictly confidential.

8.2 Data recording and source data

8.2.1 eCRF

The eCRF will be implemented in a GCP-compliant clinical trial management system (CDMS) (secuTrial®) hosted at CTU, University of Bern, Bern. The CDMS is activated for the trial only after successfully passing a formal test procedure. All data entered in the eCRFs are stored on a Linux server in a dedicated Oracle database. Only eCRFs will be used. Templates of blank worksheets are documented in the trial master file (TMF).

Trained study personnel will enter baseline, and FU data for each enrolled participant in the centralized, trial-specific eCRF. All processes regarding the CDMS follow standard operating procedures of CTU, University of Bern, Bern. Access to the system will be granted only after documented training.

In case of questions for data recording in the eCRF, investigators can send an email to DeintensiF@insel.ch or contact the study coordinator (contact details see **Appendix 4**).

For technical difficulties concerning the database and randomization, investigators are recommended to contact data management of CTU, University of Bern, Bern, Switzerland.

E-mail: datamanagement@ctu.unibe.ch

In order to receive authorization for online registration/data entry, sites must send a copy of the completed staff list and training logs to CTU, University of Bern, Bern. Login details for the online database will be sent to authorized persons.

8.2.2 Paper and electronic patient reported outcome questionnaire

Data from the pPRO will be entered by the trained study personnel into the CDMS for data analysis.

Data from the ePRO will be transferred to the study database hosted by CTU Bern for data analysis. Data security and data handling and processing will be compliant to GCP, FADP [117] and GDPR [118].

Since the ePRO is still in development and the process will be revised based on the experience from the pilot phase of the study, details on data security and data processing will be provided in the protocol upon a major amendment of the protocol for EC approval before implementation.

8.2.3 Source data

Source data must be available at the site to document the existence of the trial participants.

The following source documents will be stored at the local trial site:

- Informed consent form
- Patient (pre-)screening, enrolment and identification log
- Patient records and medical history including reports from the treating physician
- Documentation of the patients' medical treatment
- Imaging acquisitions including corresponding reports
- Laboratory reports
- Questionnaires
- Paper PROs
- Worksheets
- Any other relevant record to document SAEs

Trained study personnel will enter the source data in the eCRF.

8.3 Confidentiality and coding

Source data containing participant identifying information will be kept at the participating sites. Direct access to source documents will be permitted for purposes of monitoring, audits, and inspections. The monitoring institution (i.e. CTU, University of Bern, Bern) and the local authorities will have access to all information necessary for such tasks during and after the study. If any source data needs to be transferred out of the participating site, all personal information must be redacted before any data transfer.

A unique participant identification number will be attributed to each participant registered for the trial. For each enrolled trial participant, an eCRF will be maintained. Coded identification for each participant will be as follows:

1685-[site-no]-consecutive number

Authorized to enter data into the eCRF are the local trial team staff according to the authorization list. Authorized persons will be identified by their usernames. The local PI is responsible for proper training and instruction of the trial personnel in filling data into the eCRF.

Identification of participants must be guaranteed at each site using the patient screening, enrolment and identification list. The participant identification list will be stored under the responsibility of the PI in the investigator site file. Participant confidentiality will be maintained according to local applicable legislation. The PI at each study site safeguards the confidentiality of participating patients' data, ensuring that no participant information containing identifying data will leave the study site.

8.4 Retention and destruction of study data and biological material

For interim and final analyses, data files will be extracted from the database into statistical packages to be analyzed. Before database lock, the PI will validate the data collected at his or her site with his or her signature. After database lock, the status of the database is recorded in special archive tables.

All study data is archived for 10 years after study termination or premature termination of the study.

Data collected in this study will be stored and/or possibly transmitted in coded form to other researchers and may be used for both open- or non-open access publications or databases, as well as for future research projects that are yet to be defined. Only data from participants who provided consent for further use of their data will be used for future research projects. Any further use of the data for research projects will be submitted to the EC for approval before conduct.

No biological materials will be collected or retained in this study.

9 MONITORING AND STUDY REGISTRATION

9.1 Monitoring

On-site and centralized monitoring (including central data monitoring) will be part of the quality control activities implemented for this study. Interim on-site monitoring visits will be performed either at pre-defined time points during study conduct or visits may be scheduled based on key performance indicators. Sites may also be selected for on-site monitoring by chance. Monitoring will be performed according to a separate monitoring plan.

For the purposes of monitoring, the PI at each site will provide the monitor with access to study documentation, patient records, facilities, and any other resources as necessary. During on-site monitoring visits, the PI or his/her designee will support the monitor in his/her activities and answer questions arising. Questions and queries arising during centralized monitoring will be handled in a timely manner.

All involved parties will keep participant data strictly confidential.

9.2 Study registration

The trial is registered on clinicaltrials.gov (NCT05388136) and on the Swiss National Clinical Trial Portal (SNCPT000005198).

10 FUNDING / PUBLICATION / DECLARATION OF INTEREST

10.1 Funding

The pilot study is supported by funding of Swiss cancer research - Health Services Research: 250,000.- CHF.

Other submitted grant application includes:

SNSF Grant (IICT) submitted 11/2022: 3,300,000.- CHF

10.2 Publication

A public website providing details on the trial will be implemented before enrolment and regularly updated. We will publish the trial protocol in abbreviated form in an open-access journal.

First data analysis based on the results of the interim safety analysis may be communicated precociously, due to the possible impact on the trial and FU guidelines. The report will therefore only be made available by the trial statistician to the DMC and SNSF (unless the trial is stopped early for safety reasons in which case the interim safety analysis forms the final analysis).

The publication presenting the main findings of the trial will be written based on the final analysis report and will be published in a peer-reviewed open-access journal within one year after the database lock. Additional analyses will be published only after the main publication is publicly available. Investigators from the participating centres are members of corresponding medical societies respectively guideline committees in their respective countries. They will ensure that results will appropriately be reflected in national and, if possible, international guidelines.

Approaches to presenting results to the public will be discussed with the Patient Advisory Board. As a minimum, each participant will be informed after the main publication. In addition, formats such as patient congresses will be evaluated.

Once results have been published, trial data will be accessible to external researchers, and anonymized datasets corresponding to each publication will be made available. Investigators wishing to replicate the analyses or to do an individual patient meta-analysis may request the data from the steering committee. Access to data will be granted in an unbureaucratic way and after signature of a Data Transfer Agreement.

10.3 Declaration of interest

There are no conflicts of interest or conflicts of private, economical, or financial interest to declare. The parties agree with these statements by signing this trial protocol.

11 REFERENCES

1. Stenson, K.M., *Epidemiology and risk factors for head and neck cancer.* UpToDate, 2022.
2. *Bernier J, Domenge C, Ozsahin M, et al. Postoperative Irradiation with or without concomitant chemotherapy for locally advanced head and neck cancer. N Engl J Med 2004; 350: 1945–52.*
3. *Cooper JS, Pajak TF, Forastiere AA, et al. Postoperative Concurrent Radiotherapy and Chemotherapy for High-Risk Squamous-Cell Carcinoma of the Head and Neck. N Engl J Med 2004; 3350: 1937-44.*
4. *Bourhis J, Overgaard J, Audry H, et al. on behalf of the Meta-analysis of Radiotherapy in Carcinomas of Head and Neck (MARCH) Collaborative Group. Hyperfractionated or accelerated radiotherapy in head and neck cancer: a meat-analysis. Lancet 2006; 368: 843-54.*
5. *Pignon JP, Le Maître A, Bourhis J. et al. on behalf of the MACH-NC Collaborative Group. Meta-analysis of chemotherapy in head and neck cancer (MACH-NC): An update on 93 randomized trials and 17,346 patients. Rad Oncol 2009; 92: 4-14.*
6. *Cooper JS, Pajak TF, Rubin P, et al. Second malignancies in patients who have head and neck cancer: incidence, effect on survival and implications based on the RTOG experience. Int J Radiat Oncol Biol Phys 1989; 17: 449-456.*
7. *Gregoire V, Lefebvre JL, Licitra L, Felip E. EHNS-ESMO-ESTRO Guidelines Working Group. Squamous cell carcinoma of the head and neck: EHNS-ESMO-ESTRO Clinical Practice Guidelines for diagnosis, treatment and follow-up. Ann Oncol 2010; 21 Suppl 5: v184-6.*
8. *Yamamoto E, Shibuya H, Mura M. Site specific dependency of second primary cancer in early stage head and neck squamous cell carcinoma. Cancer 2002; 94: 2007-14.*
9. *Ang KK, Harris J, Wheeler R, et al. Human papillomavirus and survival of patients with oropharyngeal cancer. N Engl J Med 2010; 363: 24-35.*
10. *Pulte D, Brenner H. Changes in survival in head and neck cancers in the late 20th and early 21st century: a period analysis. Oncologist 2010; 15: 994-1001.*
11. *Roman BR, Goldenberg D, Givi B. Education Committee of American Head and Neck Society (AHNS). AHNS Series-Do you know your guidelines?: guideline recommended follow-up and surveillance of head and neck cancer survivors. Head Neck 2016; 38:168-74.*
12. *Digonnet A, Hamoir M, Andry G, et al. Post-therapeutic surveillance strategies in head and neck squamous cell carcinoma. Eur Arch Otorhinolaryngol 2013; 270:1569-80.*
13. *NCCN Clinical Practice Guidelines in Oncology (NCCN Guidelines®): Head and Neck Cancers, Version 3.2021.*
14. *Hermans R, Pameijer FA, Mancuso AA, et al. Laryngeal or hypopharyngeal squamous cell carcinoma: can follow-up CT after definitive radiation therapy be used to detect local failure earlier than clinical examination alone? Radiology 2000; 214: 683-7.*
15. Paniello, R.C., et al., *Practice patterns and clinical guidelines for posttreatment follow-up of head and neck cancers: a comparison of 2 professional societies.* Arch Otolaryngol Head Neck Surg, 1999. **125**(3): p. 309-13.
16. *Practice care guidance for clinicians participating in the management of head and neck cancer patients in the UK. Drawn up by a Consensus Group of Practising Clinicians.* Eur J Surg Oncol, 2001. **27 Suppl A**: p. S1-17.
17. Kaanders, J.H. and G.J. Hordijk, *Carcinoma of the larynx: the Dutch national guideline for diagnostics, treatment, supportive care and rehabilitation.* Radiother Oncol, 2002. **63**(3): p. 299-307.
18. Lester, S.E. and R.G. Wight, *'When will I see you again?' Using local recurrence data to develop a regimen for routine surveillance in post-treatment head and neck cancer patients.* Clin Otolaryngol, 2009. **34**(6): p. 546-51.
19. *Associazione Italiana di Radioterapia Oncologica (AIRO). Linee guida AIRO [Internet]. Roma: Associazione Italiana di Radioterapia Oncologica; [cited 2016 May 8].* . Available from: http://www.radioterapiaitalia.it/cont_169.phtml.
20. *Associazione Italiana di Oncologia Medica (AIOM). Linee guida AIOM [Internet]. Milano: Associazione Italiana di Oncologia Medica; [cited 2016 May 8].* . Available from: http://www.aiom.it/professionisti/documenti-scientifici/linee-guida/1%2C413%2C1%2C#TopList.
21. Robard, L., et al., *Guidelines of the French Society of Otorhinolaryngology (SFORL) (short version). Specific treatment of epistaxis in Rendu-Osler-Weber disease.* Eur Ann Otorhinolaryngol Head Neck Dis, 2017. **134**(1): p. 37-41.
22. *SSORL - Swiss-Guidelines: Empfehlungen für die Betreuung (Abklärungen, Behandlung und Nachsorge) von Patienten mit Karzinomen des Kopf-Hals-Bereiches. Konsensusbericht der Arbeitsgemeinschaft für Hals- und Gesichtschirurgie der Schweizerischen Gesellschaft für ORL, Hals- und Gesichtschirurgie, Ausgabe 2019.*
23. Machiels, J.P., et al., *Squamous cell carcinoma of the oral cavity, larynx, oropharynx and hypopharynx: EHNS-ESMO-ESTRO Clinical Practice Guidelines for diagnosis, treatment and follow-up.* Ann Oncol, 2020. **31**(11): p. 1462-1475.
24. *NCCN Guidlines.* 2020, National Comprejensive Cancer Network Clinical Practice Guidelines in Oncology.
25. *NCCN Clinical Practice Guidelines in Oncology (NCCN Guidelines®): Lung Cancer Screening, Version 1.2022.*
26. *De Visscher AV, Manni JJ. Routine long-term follow-up in patients treated with curative intent for squamous cell carcinoma of the larynx, pharynx, and oral cavity. Does it make sense? Arch Otolaryngol Head Neck Surg 1994; 120: 934-9.*
27. *Ritoe SC, de Vegt F, Scheike IM, et al. Effect of routine follow-up after treatment for laryngeal cancer on life expectancy and mortality: results of a Markov model analysis. Cancer 2007; 109: 239-47.*
28. *Agrawal A, Hammond TH, Young GS, et al. Factors affecting long-term survival in patients with recurrent head and neck cancer may help define the role of post-treatment surveillance. Laryngoscope 2009; 119: 2135-40.*

29. *Boysen M, Lövdal O, Tausjö J, et al. The value of follow-up in patients treated for squamous cell carcinoma of the head and neck. Eur J Cancer 1992; 28: 426-30.*
30. *Flynn CJ, Khaouam N, Gardner S, et al. The value of periodic follow-up in the detection of recurrences after radical treatment in locally advanced head and neck cancer. Clin Oncol 2010; 22: 868-73.*
31. *Imbimbo M, Alfieri S, Botta L, et al. Surveillance of Patients with Head and Neck Cancer with an Intensive Clinical and Radiologic Follow-up. Otolaryngol Head Neck Surg 2019;161: 635-42.*
32. *Kissun D, Magennis P, Lowe D, et al. Timing and presentation of recurrent oral and oropharyngeal squamous cell carcinoma and awareness in the outpatient clinic. Br J Oral Maxillofac Surg 2006; 44: 371-6.*
33. *Schwartz DL, Barker J Jr, Chansky K, et al. Postradiotherapy surveillance practice for head and neck squamous cell carcinoma--too much for too little? Head Neck 2003; 25: 990-9.*
34. *Francis DO, Yueh B, Weymuller EA Jr, et al. Impact of surveillance on survival after laryngeal cancer in the medicare population. Laryngoscope 2009; 119: 2337–44.*
35. *Boysen ME, Zätterström UK, Evensen JF. Self-reported Symptoms to Monitor Recurrent Head and Neck Cancer-Analysis of 1,678 Cases. Anticancer Res 2016; 36: 2849-54.*
36. *Kothari P, Trinidade A, Hewitt RJD, et al. The follow-up of patients with head and neck cancer: an analysis of 1,039 patients. Eur Arch Otorhinolaryngol 2011; 268:1191-1200.*
37. *Pagh A, Vedtofte T, Lynggaard CD, et al. The value of routine follow-up after treatment for head and neck cancer. A national survey from DAHANCA. Acta Oncol 2013; 52: 277-84.*
38. *Ilmarinen T, Keski-Säntti H, Markkanen-Leppänen M, et al. De-escalation of post-treatment surveillance in oropharyngeal cancer. Head Neck 2018. DOI; 10.1002/hed.25593.*
39. Masroor, F., et al., *Association of NCCN-Recommended Posttreatment Surveillance With Outcomes in Patients With HPV-Associated Oropharyngeal Squamous Cell Carcinoma.* JAMA Otolaryngol Head Neck Surg, 2019. **145**(10): p. 903-908.
40. *Jung YH, Song CM, Park JH, et al. Efficacy of current regular follow-up policy after treatment for head and neck cancer: Need for individualized and obligatory follow-up strategy. Head Neck 2014; 36: 715-21.*
41. *Agrawal A, de Silva BW, Buckley BM, Schuller DE. Role of the physician versus the patient in the detection of recurrent disease following treatment for head and neck cancer. Laryngoscope 2004; 114: 232-5.*
42. *Nisa L, La Macchia R, Boujelbene N, et al. Correlation between subjective evaluation of symptoms and objective findings in early recurrent head and neck squamous cell carcinoma. JAMA Otolaryngol Head Neck Surg 2013; 139: 687-93.*
43. *Basch E, Deal AM, Kris MG, et al. Symptom Monitoring With Patient-Reported Outcomes During Routine Cancer Treatment: A Randomized Controlled Trial. J Clin Oncol 2016; 34: 557-65.*
44. *Denis F, Basch E, Septans AL, et al. Two-year survival comparing web-based symptom monitoring vs routine surveillance following treatment for lung cancer. JAMA 2019; 321: 306-7.*
45. Boysen, M., et al., *The value of follow-up in patients treated for squamous cell carcinoma of the head and neck.* Eur J Cancer, 1992. **28**(2-3): p. 426-30.
46. Zätterström, U., M. Boysen, and J.F. Evensen, *Significance of self-reported symptoms as part of follow-up routines in patients treated for oral squamous cell carcinoma.* Anticancer Res, 2014. **34**(11): p. 6593-9.
47. De Zoysa, N., et al., *Developing a follow-up surveillance protocol in head and neck oncological surgery: enhanced 'traffic light' surveillance - a prospective feasibility study.* Clin Otolaryngol, 2017. **42**(2): p. 446-450.
48. *Chan KC, Hung EC, Woo JK, et al. Early detection of nasopharyngeal carcinoma by plasma Epstein-Barr virus DNA analysis in a surveillance program. Cancer 2013; 119: 1838–44.*
49. *Hanna GJ, Supplee JG, Kuang Y, et al. Plasma HPV cell-free DNA monitoring in advanced HPV-associated oropharyngeal cancer. Ann Oncol 2018; 29: 1980–6.*
50. *Szturz P, Van Laer C, Simon C, et al. Follow-Up of Head and Neck Cancer Survivors: Tipping the Balance of Intensity. Front Oncol 2020; 6; 10: 688. doi: 10.3389/fonc.2020.00688. eCollection 2020.*
51. *Lee DH, Roh JL, Baek S, et al. Second cancer incidence, risk factor, and specific mortality in head and neck squamous cell carcinoma. Otolaryngol Head Neck Surg 2013; 149: 579-86.*
52. *Jain KS, Sikora AG, Baxi SS, Morris LG. Synchronous cancers in patients with head and neck cancer: risks in the era of human papillomavirus-associated oropharyngeal cancer. Cancer 2013; 119: 1832-7.*
53. Malik, A., et al., *Outcomes of a Telephone-Based Questionnaire for Follow-up of Patients Who Have Completed Curative-Intent Treatment for Oral Cancers.* JAMA Otolaryngol Head Neck Surg, 2020. **146**(12): p. 1102-1108.
54. *Kim AJ, Suh JD, Sercarz JA, et al. Salvage surgery with free flap reconstruction: factors affecting outcome after treatment of recurrent head and neck squamous carcinoma. Laryngoscope 2007; 117:1019-23.*
55. *Tan HK, Giger R, Auperin A, et al. Salvage surgery after concomitant chemoradiation in head and neck squamous cell carcinomas - stratification for postsalvage survival. Head Neck 2010; 32: 139-47.*
56. *Zafereo ME, Hanasono MM, Rosenthal DI, et al. The role of salvage surgery in patients with recurrent squamous cell carcinoma of the oropharynx. Cancer 2009; 115: 5723-33.*
57. *Sullivan BP, Parks KA, Dean NR, et al. Utility of CT surveillance for primary site recurrence of squamous cell carcinoma of the head and neck. Head Neck 2011; 33:1547–50.*
58. *Hermans R. Posttreatment imaging in head and neck cancer. EurJRadiol 2008; 66: 501–11.*
59. *Kangelaris GT, Yom SS, Huang K, Wang SJ. Limited utility of routine surveillance MRI following chemoradiation for advanced-stage oropharynx carcinoma. Int J Otolaryngol 2010; 2010: 904297. doi: 10.1155/2010/904297.*

60. *Vandecaveye V, De Keyzer F, Nuyts S, et al. Detection of head and neck squamous cell carcinoma with diffusion weighted MRI after (chemo)radiotherapy: correlation between radiologic and histopathologic findings. Int J Radiat Oncol Biol Phys 2007; 67: 960–71.*
61. Hemmat, S.M., et al., *The Limitations of Routine Magnetic Resonance Imaging for Long-Term Post-Treatment Locoregional Surveillance of Salivary Carcinoma.* ORL J Otorhinolaryngol Relat Spec, 2015. **77**(3): p. 132-40.
62. *Kao J, Vu HL, Genden EM, et al. The diagnostic and prognostic utility of positron emission tomography/computed tomography-based follow-up after radiotherapy for head and neck cancer. Cancer 2009;115: 4586-94.*
63. *Krabbe CA, Pruim J, Dijkstra PU, et al. 18F-FDG PET as a routine posttreatment surveillance tool in oral and oropharyngeal squamous cell carcinoma: a prospective study. J Nucl Med 2009; 50: 1940-7.*
64. *Manikantan K, Dwivedi RC, Sayed SI, et al. Current concepts of surveillance and its significance in head and neck cancer. Ann R Coll Surg Engl 2011; 93: 576-82.*
65. *Wang SJ. Surveillance radiologic imaging after treatment of oropharyngeal cancer: a review. World J Surg Oncol 2015; 13: 94.*
66. *Ho AS, Tsao GJ, Chen FW, et al. Impact of positron emission tomography/computed tomography surveillance at 12 and 24 months for detecting head and neck cancer recurrence. Cancer 2013; 119: 1349-56.*
67. *Dunsky KA, Wehrmann DJ, Osman MM, et al. PET-CT and the detection of the asymptomatic recurrence or second primary lesions in the treated head and neck cancer patient. Laryngoscope 2013; 123: 2161-4.*
68. *Mehanna H, Wong WL, McConkey CC, et al. PET-NECK Trial Management Group. PET-CT Surveillance versus Neck Dissection in Advanced Head and Neck Cancer. N Engl J Med 2016: 14; 374: 1444-54.*
69. *Corpman DW, Masroor F, Carpenter DM, et al. Posttreatment surveillance PET/CT for HPV-associated oropharyngeal cancer. Head Neck 2019; 41: 456-462.*
70. *Warner GC, Cox GJ. Evaluation of chest radiography versus chest computed tomography in screening for pulmonary malignancy in advanced head and neck cancer. J Otolaryngol 2003; 32: 107-9.*
71. *Hsu Y-B, Chu P-Y, Liu J-C, et al. Role of chest computed tomography in head and neck cancer. Arch Otolaryngol Head Neck Surg 2008; 134: 1050-54.*
72. *Aberle DR, Adams AM, Berg CD, et al. Reduced lung-cancer mortality with low-dose computed tomographic screening. N Engl J Med 2011; 365: 395-409.*
73. *Church TR, Black WC, Aberle DR, et al. Results of initial low-dose computed tomographic screening for lung cancer. N Engl J Med 2013; 368:1980-91.*
74. *de Koning HJ, van der Aalst CM, de Jong PA, et al. Reduced Lung-Cancer Mortality with Volume CT Screening in a Randomized Trial (NELSON trial). Randomized Controlled Trial N Engl J Med 2020; 382: 503-13.*
75. *Cheung MK, Ong SY, Goyal U, et al. False Positive Positron Emission Tomography / Computed Tomography Scans in Treated Head and Neck Cancers. Cureus 2017; 9: e1146. doi:10.7759/cureus.1146.*
76. *Pagedar NA, Jayawardena A, Charlton ME, Hoffman HT. Second Primary Lung Cancer After Head and Neck Cancer: Implications for Screening Computed Tomography. Ann Otol Rhinol Laryngol 2015; 124: 765-9.*
77. *Ghazali N, Cadwallader E, Lowe D, et al. Fear of recurrence among head and neck cancer survivors: longitudinal trends. Psychooncol 2013; 22: 807-13.*
78. *Ghazali N, Roe B, Lowe D, et al. Uncovering patients' concerns in routine head and neck oncology follow up clinics: an exploratory study. Br J Oral Maxillofac Surg 2013; 51: 294-300.*
79. *Koch L, Jansen L, Brenner H, Arndt V. Fear of recurrence and disease progression in long-term (≥5 years) cancer survivors-a systematic review of quantitative studies. Psychooncol 2013; 22:1–11.*
80. *Lampic C, Wennberg A, Schill JE, et al. Anxiety and cancer-related worry of cancer patients at routine follow-up visits. Acta Oncol 1994; 33:119–25.*
81. *Handschel J, Naujoks C, Kübler NR, Krüskemper G. Fear of recurrence significantly influences quality of life in oral cancer patients. Oral Oncol 2012, 48: 1276-80.*
82. *Kanatas A, Ghazali N, Lowe D, et al. Issues patients would like to discuss at their review consultation: variation by early and late stage oral, oropharyngeal and laryngeal subsites. Eur Arch Oto-Rhino-Laryngology 2013; 270: 1067–74.*
83. *Rogers SN, Scott B, Lowe D, et al. Fear of recurrence following head and neck cancer in the outpatient clinic. Eur Arch Oto-Rhino-Laryngology 2010; 267: 1943-9.*
84. *Rogers SN, Cross B, Talwar C, et al. A single-item screening question for fear of recurrence in head and neck cancer. Eur Arch Oto-Rhino-Laryngology 2016; 273:1235-42.*
85. *Mueller SA, Riggauer J, Elicin O, et al. Patients' preferences concerning follow-up after curative head and neck cancer treatment: A cross-sectional pilot study. Head Neck 2019;41: 2174-81.*
86. *Roman BR, Goldenberg D, Givi B; Education Committee of American Head and Neck Society (AHNS). AHNS Series–Do you know your guidelines? Guideline recommended follow-up and surveillance of head and neck cancer survivors. Head Neck 2016; 38: 168–74.*
87. *Portman D. Scanxiety. Fam Syst Health 2018; 36: 415. doi: 10.1037/fsh0000353.*
88. *Tribius S, Meyer MS, Pflug C, et al. Socioeconomic status and quality of life in patients with locally advanced head and neck cancer. Strahlenther Onkol 2018; 194:737-49.*
89. *van Agthoven M, van Ineveld BM, de Boer MF, et al. The costs of head and neck oncology: primary tumours, recurrent tumours and long-term follow-up. Eur J Cancer. 2001; 37:2204-11.*

90. *Neuenschwander B, Rouyrre N, Hollaender N, et al. A proof of concept phase II non-inferiority criterion. Stat Med 2011; 30: 1618-27.*
91. *Roychoudhury S, Scheuer N, Neuenschwander B. Beyond p-values: A phase II dual-criterion design with statistical significance and clinical relevance. Clin Trials 2018; 15: 452-61.*
92. *Wissinger E, Griebsch I, Lungershausen J, et al. The economic burden of head and neck cancer: a systematic literature review. Pharmacoeconomics 2014; 32: 865-82.*
93. Avery, K.N., et al., *Informing efficient randomised controlled trials: exploration of challenges in developing progression criteria for internal pilot studies.* BMJ Open, 2017. **7**(2): p. e013537.
94. *International Conference on Harmonization (ICH) E6(R2) Guideline for Good Clinical Practice.*
95. Lin, Y., M. Zhu, and Z. Su, *The pursuit of balance: An overview of covariate-adaptive randomization techniques in clinical trials.* Contemp Clin Trials, 2015. **45**(Pt A): p. 21-5.
96. Lauzon, S.D., et al., *Statistical properties of minimal sufficient balance and minimization as methods for controlling baseline covariate imbalance at the design stage of sequential clinical trials.* Stat Med, 2020. **39**(19): p. 2506-2517.
97. Roberts NJ, E.G., Blenkhorn P, Partridge MR. Nigel Masters, Catherine Tutt, *NHS Atlas of Variation in Healthcare for People with Respiratory Disease*
98. Wood, D.M., et al., *"Pack year" smoking histories: what about patients who use loose tobacco?* Tob Control, 2005. **14**(2): p. 141-2.
99. Group, E.-A.C.R., *ECOG/WHO performance scale* 1982.
100. Rogers, S.N., et al., *A single-item screening question for fear of recurrence in head and neck cancer.* Eur Arch Otorhinolaryngol, 2016. **273**(5): p. 1235-42.
101. Koller, M., et al., *Translation procedures for standardised quality of life questionnaires: The European Organisation for Research and Treatment of Cancer (EORTC) approach.* Eur J Cancer, 2007. **43**(12): p. 1810-20.
102. *Fayers P, Bottomley A; EORTC Quality of Life Group; Quality of Life Unit. Quality of life research within the EORTC-the EORTC QLQ-C30. European Organisation for Research and Treatment of Cancer. Eur J Cancer 2002; 38 Suppl 4: S125-33.*
103. *Singer S, Amdal CD, Hammerlid E, et al. EORTC Quality of Life and the EORTC Head and Neck Cancer Groups. International validation of the revised European Organisation for Research and Treatment of Cancer Head and Neck Cancer Module, the EORTC QLQ-HN43: Phase IV. Head Neck 2019; 41: 1725-37.*
104. *EuroQol--a new facility for the measurement of health-related quality of life.* Health Policy, 1990. **16**(3): p. 199-208.
105. *Royston P, Parmar MK. The use of restricted mean survival time to estimate the treatment effect in randomized clinical trials when the proportional hazards assumption is in doubt. Stat Med 2011; 30: 2409-21.*
106. *Royston P. Estimating the treatment effect in a clinical trial using difference in restricted mean survival time. Stata J 2015; 15: 1098-1117.*
107. *Murray EJ, Swanson SA, Hernan MA. Guidelines for estimating causal effects in pragmatic randomized trials (last updated: Nov 19, 2019). Available at: https://arxiv.org/abs/1911.06030v2. Accessed: Feb 20, 2020.*
108. *Mozumder SI, Rutherford MJ, Lambert PC. Estimating restricted mean survival time and expected life-years lost in the presence of competing risks within flexible parametric survival models. BMC Med Res Methodol 2021; 21: 52. doi: 10.1186/s12874-021-01213-0.*
109. *Ibrahim JG, Chu H, Chen LM. Basic concepts and methods for joint models of longitudinal and survival data. J Clin Oncol 2010; 28: 2796-2801.*
110. *Tsiatis AA, Davidian M. Joint modeling of longitudinal and time to event data: an overview. Stat Sin 2004; 14: 809?34.*
111. *Shakespeare TP, Gebski VJ, Veness MJ, et al. Improving interpretation of clinical studies by use of confidence levels, clinical significance curves, and risk-benefit contours. Lancet 2001; 357: 1349-53.*
112. *Spiegelhalter DJ, Abrams KR, Myles JP. Bayesian approaches to clinical trials and health care evaluation. 2004, Chichester: John Wiley & Sons.*
113. *Declaration of Helsinki.*
114. *Human Research Act (HRA).*
115. *Ordinance on Clinical Trials in Human Research (ClinO).*
116. *International Conference on Harmonization (ICH) E2A Clinical Safety Data Management: Definitions and Standards for Expedited Reporting.*
117. *Federal Act on Data Protection (FADP).*
118. *Regulation (EU) 2016/679 of the European Parliament and of the Council. General Data Protection Regulation (GDPR).* Official Journal of the European Union.

12 CHANGE HISTORY

Version	Date	Chapter	Summary of changes
1.0	18.05.2021	N/A	N/A – new document
1.1	05.07.2022	2.1<br>4.2.1<br><br>4.2.1<br>4.3.3<br><br>4.7/4.8<br><br>5.1.2<br><br>8.2	Updated disease background<br>Explained the aim and the procedure of the participation questionnaire at recruitment<br>Detailed the procedure on participation questionnaire<br>Added acquiring the information on the use of electronic devices and internet<br>Updated the information collected for cost analysis<br>Separated discontinuation and withdrawal and clarified both conditions<br>Added analysis of the participation questionnaire<br>Clarified the role of DMC during interim analyses<br>Rearranged Data recording and source data
2.0	15.9.2022	4.1.2<br><br>4.3<br><br><br><br><br><br><br><br><br>Appendix 4	Clarified inclusion criteria 4, and added new exclusion criteria 4<br>- Clarified stage selection in case of synchronous tumor for randomization<br>- Added in level of education “No formal education”<br>- Removed record of date of multidisciplinary tumor board decision<br>- Updated ECOG table according to the latest version<br>- Added a new concomitant care and cost questionnaire to capture additional care and cost, refined record of all concomitant cost<br>Updated PAB member list
3.0	14.11.2022	3.3<br>4.1.2<br><br>4.3.3<br><br><br><br><br><br><br><br><br>4.3.4<br>4.7- 4.8<br><br><br>4.9<br>6.2<br><br>6.4<br>11<br>Appendix 1<br><br><br>Appendix 2	Corrected withdrawal to discontinuation<br>Clarified inclusion criteria 1 except lip<br>Updated notes of inclusion criteria 4<br>- Added smoking pack-years calculator<br>- Added section on training on signs and symptoms alarming for REC and SPM<br>- Clarified AE and PRO are recorded also in each scheduled and non-scheduled visit<br>- Added section “Instruction and training on signs and symptoms alarming for REC and SPM”<br>- Added section “other evaluation and supportive care”<br>Clarified discontinuation/withdrawal criteria<br>- Re-defined SAE reporting criteria and defined method of assessment for AEs at the discretion of each site<br>- Added section End of study<br>- Revised pregnancy reporting procedure as discontinuation but not as an SAE<br>Updated schedule and clarified according to changes in the protocol<br>Added change history<br>Updated visit schedule for AEs, training on PROs and more help text are added, clarified Pro completion at every visit<br>Complete revision of the questions and conditions of the PRO and changed to English version.
3.1	23.11.2022	4.1.1<br>3.5, 4.3.5<br><br><br>6.2<br>Appendix 1<br><br>Appendix 2	Synchronize inclusion criteria with synopsis<br>Clarified during the pilot study, signs of REC/SPM indicated by the PRO results are assessed by the study team during a telephone call.<br>Restructured section on SAE reporting<br>Added column during non-scheduled visit<br>Added description on assessment of alert for REC/SPM during the pilot study.<br>Death is reported in end of study instead of SAE.

		Appendix 3	

13 APPENDIXES

Appendix 1: Schedule of assessments
Appendix 2: Patient-report outcomes questionnaire
Appendix 3: List of selected adverse events
Appendix 4: List of involved institutions

Appendix 1: Schedule of assessments

Individualized de-intensified and conventional follow-up schedule

		Year 1					Year 2				Year 3				Year 4		Year 5		
**X: For both arms**<br>**O: For conventional arm only**	Before inclusion[1]	Months after randomization (+/- 4 weeks)[2]																	Non-scheduled visit at anytime
		0	3	6	9	12	15	18	21	24	27	30	33	36	42	48	54	60	
Patient pre-screening	X																		
Oral (recruitment flyers) and written patient information (PISIC)	X																		
Participation questionnaire	X[8]																		
MRI or CT Head and Neck with contrast and CT covering chest to pelvis in T2 N0 / PET/CT or PET/MRI for ≥ T3 and/or N+	X[4]																		
Inclusion and exclusion criteria[3]	X	X																	
Obtain signed informed consent		X																	
Randomization		X																	
Patient tumor, treatment characteristics		X																	
Height measurement		X																	
Weight measurement		X	O	X	O	X	O	X	O	X	O	X	O	X	X	X	X	X	X
Physical exam with fiber-/rigid endoscopy		X[9]	O	X	O	X	O	X	O	X	O	X	O	X	X	X	X	X	X
Instruction and training of signs/symptoms for REC/SPM, use of paper-/web-based PRO questionnaire and self-referral (Baseline, later if needed)		X	O	X	O	X	O	X	O	X	O	X	O	X	X	X	X	X	X
Assessment of signs and symptoms using the patient-reported outcomes (PRO) questionnaire		X	O	X	O	X	O	X	O	X	O	X	O	X	X	X	X	X	X
Schedule compliance, number of visit rescheduling			O	X	O	X	O	X	O	X	O	X	O	X	X	X	X	X	
Assessment of REC/SPM			O	X	O	X	O	X	O	X	O	X	O	X	X	X	X	X	X
Assessment self-referral/PRO alert-initiated visits, other cancer-/treatment-related visits/procedures																			X
Tobacco/Alcohol consumption		X		X		X		X		X		X		X	X	X	X	X	
Assessment of specific treatment-related adverse events[10] (CTCAE),SAE and outcome (since therapy or last assessment)[11]		X	O	X	O	X	O	X	O	X	O	X	O	X	X	X	X	X	X
Performance status assessment (ECOG)		X		X		X		X		X		X		X	X	X	X	X	
Health-related Quality of Life (EORTC QLQ-C30 and QLQ-H&N43)		X		X		X		X		X		X		X	X	X	X	X	
EQ-5D-5L		X				X				X				X		X		X	
Concomitant care questionnaire				X		X		X		X		X		X	X	X	X	X	
MRI or CT Head and Neck with contrast and chest CT with contrast[12]				O				O											
Chest CT without contrast[5,12]												(O)			(O)		(O)		
Patient-reported outcomes questionnaire recording by the patient		X	Monthly for both arms																
Investigation / Imaging / Pandendoscopy			In case of clinical suspicion of REC/SPM[6]																
Pregnancy test for women with childbearing potential[7]		X	Before each CT																

I0I Baseline: timepoint of patient enrollment and randomization at 6 months after treatment

[1] 3 - 6 months after therapy

[2] In case of rescheduling by patient as soon as possible within 2 weeks

[3] Before inclusion, pre-screening of eligibility base on available clinical data per-routine, confirmation of all eligibility criteria after informed consent had been obtained.

[4] If PET/CT or PET/MRI not available for ≥ T3 and/or N+: MRI or CT Head and Neck with contrast and CT covering chest to pelvis

[5] Chest CT without contrast marked with (O) is planned in participants with smoking history only (active smoking and ≥20 pack-years, or smoking habits at least within the last 15 years before cancer diagnosis and ≥20 pack-years). Current active smoker or former smoker of less than 20 pack-years do not get regular chest CT.

[6] REC/SPM: recurrence/second primary malignancy. According to the participating centers' standards

[7] Urine or blood test according to the standard of the center

[8] Pilotphase only

[9] Result maximum within 4 weeks before enrolment, ideally on the day of inclusion

[10] Assessment of the events according to appendix 3 of the protocol according to standard of each site.

11 PROs assessment should be performed at each visit by the participant before the medical consultation with the physician (scheduled or non-scheduled), but without comparison to the PROs monitored by the patients themselves at home (just for assessment of the symptoms).

[12] Scheduled imaging can be performed up to 4 weeks before the scheduled visit.

Appendix 2: Patient-reported outcomes questionnaire

		Answers				
1.	**Weight**<br>Insert the current weight on the left (use always the same scale at home and under the same conditions) and check on the right whether the weight loss was desired or undesired	**(kg)**	Weight loss<br>☐ Unintentional<br>☐ Intentional<br>☐ No			
**Signs / Symptoms**<br>Please indicate severity of signs that have been present **for at least 3 weeks**.		**None**	**Mild**	**Moderate**	**Severe**	**Very severe**
2.	**Wound, sores or lump in mouth or throat?**	0	1	2	3	4
3.	**Blood in saliva or sputum?**	0	1	2	3	4
4.	**Ear pain on one or both sides?**	0	1	2	3	4
5.	**Palpable new lump in the neck?**	0	1	2	3	4
6.	**Hoarseness?**	0	1	2	3	4
7.	**Pain or burning sensation in mouth or throat?**	0	1	2	3	4
8.	**Difficulty swallowing?**	0	1	2	3	4
9.	**Difficulty opening the mouth?**	0	1	2	3	4
10.	**Foul smelling breath?**	0	1	2	3	4
11.	**Chest pain?**	0	1	2	3	4
12.	**Shortness of breath?**	0	1	2	3	4
13.	**Cough?**	0	1	2	3	4
14.	**Noisy breathing?**	0	1	2	3	4
15.	**Fatigue or weakness?**	0	1	2	3	4
16.	**Loss of appetite?**	0	1	2	3	4
17.	**Persistent skin lesion (whole body)?**	0	1	2	3	4
18.	**Fear of the cancer coming back?**<br>How would you describe your worst-case fear?<br>0. I have no fear of recurrence<br>1. I have a little fear, with occasional thoughts but they don't really bother me<br>2. I am sometimes having fearful thoughts but I can usually manage these<br>3. I get a lot of fears of recurrence and these can really preoccupy my thoughts<br>4. I am fearful all the time that my cancer might return and I struggle with this	0	1	2	3	4

<u>The conditions triggering automated alerts</u> to the patients in the experimental arm and respective study site are the following:

A. Undesired weight loss of >5% compared to the weight at study enrolment and continuous undesired weight loss

And/Or

B. Any increase of ≥2 grades of at least one of the signs and symptoms question compared to the next lowest previous value

And/Or

C. Any increase to "Severe/I get a lot of fears" (grade 3) or "Very severe/I am fearful all the time" (grade 4)" in at least one signs and symptoms question

And/Or

D. Any persistent rating of "Severe/I get a lot of fears" (grade 3) or "Very severe/I am fearful all the time" (grade 4) over the period of 3 months in at least one of the signs and symptoms question*

And/Or

E. Any non-completion of the signs and symptoms questionnaire after three reminders

* If a persisting grade 3 or 4 item over 3 months results in an alert and organizing of a non-scheduled clinical visit, and a recurrence of second primary tumor is ruled out after this visit, the local PI can decide that this grade 3 or 4 item is related to a non-oncologic chronic problem (e.g. chronic sequel of the treatment or co-morbidities) and may choose to ignore the subsequent alerts generated in case of grad 3 or 4 persistence through the same item in the future. However, if in between, due to any treatment attempt, there is an improvement of the signs/symptoms, and later worsening to 3 or 4, a visit has again to be organized due to a new alert.

In the main study, alerts are:

1) automatically presented to the participant within the web-based tool indicating that a clinical visit is needed, and

2) automatically sent to respective trial site. The responsible site will then actively approach the participant to arrange a visit within 2 weeks for head and neck examination.

Additional procedures and imaging according to the participating centers' standards are performed in case of signs/symptoms or physical findings suggestive for REC/SPM or treatment-related adverse events. The respective site will be alerted in case patients still do not complete the questionnaire after three reminders and calls the patient for telephone follow-up, where a new training for the regular completion of the web-based Symptom Tracker (ePRO) questionnaire will be performed.

An appointment with the appropriate support center based on centers' standards will be organized in case of triggered alerts from question 18 "Fear of the cancer coming back".

Only the patients in the experimental arm will get reminders and triggered alerts.

In the pilot study, signs of REC/SPM are assessed by the study team after evaluation of the monthly PRO results through telephone call. Arrangements for examination or consultation with the appropriate support center are the same as in the main study.

Appendix 3: List and Description of Selected Adverse Events according to CTCAE V5.0

CTCAE Term	Grade 1	Grade 2	Grade 3	Grade 4
**Dysphagia**<br>A disorder characterized by difficulty in swallowing.	Symptomatic, able to eat regular diet	Symptomatic and altered eating/swallowing	Severely altered eating/swallowing; tube feeding or total parenteral nutrition or hospitalization indicated	Life-threatening consequences; urgent intervention indicated
**Hoarseness**<br>A disorder characterized by harsh and raspy voice arising from or spreading to the larynx.	Mild or intermittent voice change; fully understandable; self-resolves	Moderate or persistent voice changes; may require occasional repetition but understandable on telephone; medical evaluation indicated	Severe voice changes including predominantly whispered speech	-
**Laryngeal Stenosis**<br>A disorder characterized by a narrowing of the laryngeal airway.	Asymptomatic; clinical or diagnostic observations only; intervention not indicated	Symptomatic (e.g., noisy airway breathing), no respiratory distress; medical intervention indicated (e.g., steroids); limiting instrumental ADL	Limiting self-care ADL; stridor; endoscopic intervention indicated (e.g., stent, laser)	Life-threatening consequences; urgent intervention indicated
**Pharyngeal Stenosis**<br>A disorder characterized by a narrowing of the pharyngeal airway.	Asymptomatic; clinical or diagnostic observations only; intervention not indicated	Symptomatic (e.g., noisy airway breathing), no respiratory distress; medical intervention indicated (e.g., steroids); limiting instrumental ADL	Limiting self-care ADL; stridor; endoscopic intervention indicated (e.g., stent, laser)	Life-threatening airway compromise; urgent intervention indicated (e.g., tracheotomy or intubation)
**Pharyngolaryngeal and/or oral pain**<br>A disorder characterized by a sensation of marked discomfort in the pharyngolaryngeal region, mouth, tongue or lips.	Mild pain	Moderate pain; limiting instrumental ADL	Severe pain; limiting self-care ADL	-
**Dental caries**<br>A disorder characterized by the decay of a tooth, in which it becomes softened, discolored and/or porous.	One or more dental caries, not involving the root	Dental caries involving the root	Dental caries resulting in pulpitis or periapical abscess or resulting in tooth loss	-
**Osteonecrosis of the jaw**<br>A disorder characterized by a necrotic process occurring in the bone of the mandible (and/or maxilla).	Asymptomatic; clinical or diagnostic observations only; intervention not indicated	Symptomatic; medical intervention indicated (e.g., topical agents); limiting instrumental ADL	Severe symptoms; limiting self-care ADL; elective operative intervention indicated	Life-threatening consequences; urgent intervention indicated
**Hypothyroidism**<br>A disorder characterized by a decrease in production of thyroid hormone by the thyroid gland.	Asymptomatic; clinical or diagnostic observations only; intervention not indicated	Symptomatic; thyroid hormones replacement indicated; limiting instrumental ADL	Severe symptoms; limiting self-care ADL; hospitalization indicated	Life-threatening consequences; urgent intervention indicated
**Fatigue**<br>A disorder characterized by a state of generalized weakness with a pronounced inability to summon sufficient energy to accomplish daily activities.	Fatigue relieved by rest	Fatigue not relieved by rest; limiting instrumental ADL	Fatigue not relieved by rest, limiting self-care ADL	-
**Accessory nerve disorder**<br>A disorder characterized by dysfunction of the abducens nerve (sixth cranial nerve).	Asymptomatic; clinical or diagnostic observations only; intervention not indicated	Moderate symptoms; limiting instrumental ADL	Severe symptoms; limiting self-care ADL	-
**Depression**<br>A disorder characterized by melancholic feelings of grief or unhappiness.	Mild depressive symptoms	Moderate depressive symptoms; limiting instrumental ADL	Severe depressive symptoms; limiting self-care ADL; hospitalization not indicated	Life-threatening consequences, threats of harm to self or others; hospitalization indicated

ADL: activities of daily living; BSA: body surface area; ROM: range of motion.
Grade 0: no symptoms. Grade 5: death directly related to the particular effect and should be reported in end of study.

Appendix 4: List of Involved Institutions

Steering committee

**Coordinating investigator**	Prof. Dr. med Roland Giger, Department of ENT, Head and Neck Surgery, Inselspital, Bern University Hospital, Bern, Switzerland
**Co-Investigator (Deputy Coordinating investigator)**	PD Dr. med Olgun Elicin, Department of Radiation Oncology, Inselspital, Bern University Hospital, Bern, Switzerland
**Co-Investigator**	Dr. med. Simon A. Müller, Department of ENT, Head and Neck Surgery, University Hospital Zurich, Zurich, Switzerland

Central service Clinical Trials Unit, University of Bern

Study management	Samantha Chan, PhD<br>Clinical Trials Unit, University of Bern	Samantha.chan@ctu.unibe.ch<br>DeintensiF@insel.ch
Trial Statistician	Andreas Limacher, PhD<br>Clinical Trials Unit, University of Bern	
Data management	Dominik Güntensperger, PhD, Clinical Trials Unit, University of Bern	datamanagement@ctu.unibe.ch
Monitor	Zoe Peña, Clinical Trials Unit, University of Bern	zoe.pena@ctu.unibe.ch

Sites and principal investigators

Pilot study

Switzerland

CH 01 Bern	Prof Dr. Roland Giger, Inselspital, Bern University Hospital
CH 02 LUKS	Prof. Dr. Rajan Gunesh, Cantonal Hospital Lucerne
CH 03 USZ	Dr. Simon Müller, University Hospital Zurich

Potential participating site for the main study

Switzerland

CH 01 Bern	Prof Dr. Roland Giger, University of Bern
CH 02 LUKS	Prof. Dr. Rajan Gunesh, Cantonal Hospital Lucerne
CH 03 USZ	Dr. Simon Müller and PD Dr. Martina Broglie Däppen, University Hospital Zurich
CH 04 Basel	PD Dr. Laurent Muller, University Hospital Basel
CH 05 Freiburg	Dr. Alexander Asanau, HFR Freiburg Kantonsspital
CH 06 Geneva	Dr. Nicolas Dulguerov, Geneva University Hospital
CH 07 Graubünden	PD Dr. Yves Brand, Kantonsspital Graubünden
CH 08 Lausanne	Prof. Dr. Christian Simon, CHUV University of Lausanne
CH 09 Neuchâtel	Dr. Yves Jaquet, Réseau Hospitalier Neuchâtelois
CH 10 St. Gallen	Prof. Dr. Sandro Stöckli, Kantonsspital St. Gallen
CH 11 Wallis	Dr. Salim Bouayed, Hospital of Valais
CH 12 Solothurn	Dr. Markus Huth, Buergerspital of Solothurn

Germany

DE 01 Ulm	Sponsor Representative and Lead Coordinating Investigator of Germany
	Prof. Dr. Patrick Schuler, University Hospital Ulm
DE 02 Düsseldorf	PD Dr. Kathrin Scheckenbach, University Hospital Düsseldorf
DE 03 Frankfurt	Dr. Sven Balster, University Hospital Frankfurt
DE 04 Göttingen	Prof. Dr. Dirk Beutner, University Medical Center Göttingen
DE 05 Heidelberg	PD Dr. Karim Zaoui, University Hospital Heidelberg
DE 06 Homburg	PD Dr. Maximilian Linxweiler, Saarland University Medical Center
DE 07 Jena	Prof. Dr. Orlando Guntinas-Lichius, University Hospital Jena
DE 09 Mannheim	Prof. Dr. Nicole Rotter, University Hospital Mannheim

DE 10 LMU- OHN	Dr. Axel Lechner, Medical Center, Department of Otorhinolaryngology, Head and Neck Surgery, University of Munich
DE 11 LMU	Prof. Dr. Sven Otto, University of Munich
DE 13 UKM	PD Dr. Achim Beule, University Hospital Münster
DE 14 Regensburg	PD Dr. Julian Künzel, University Hospital Regensburg
DE 15 Tübingen	PD Dr. Sven Becker, University Medical Center Tübingen
DE 16 Wuerzburg	PD Dr. Matthias Scheich, University of Wuerzburg
DE 17 UKW	Prof. Dr. Urs Müller-Richter, University Hospital Wuerzburg

Austria

AT 01 MedUni Wien	Prof. Dr. Markus Brunner, Medical University of Vienna
AT 02 Wien	Dr. Birgit Erlacher, Hospital Barmherzige Brüder, Wien
AT 03 Wörthersee	Dr. Hanna Lena Obermeier, Hospital Klagenfurt am Wörthersee
AT 04 Feldkirch	Dr. Lukas Poyntner, Hospital Feldkirch

France

FR 01 Nantes	Prof. Dr. Olivier Malard, University Hospital of Nantes
FR 02	Prof. Dr. Xavier Dufour; University Hospital of Poitiers

Italy

IT 01 Milan	Prof. Dr. Giuseppe Mercante, Humanitas University, Rozzano
IT 02 NCI	Prof. Dr. Alberto Deganello, National Cancer Institute, Milano
IT 03 Rome	Prof. Dr. Raul Pellini, National Cancer Institute “Regina Elena”

Belgium

BE 01 Sint-Niklaas	Dr. Willem Lybaert, AZ Nikolaas, Sint-Niklaas

Netherlands

NL 01 AMS	Dr. Willem Hans Schreuder; Netherlands Cancer Institute, Amsterdam

Patient Advisory Board

**Inselspital, Bern University Hospital**	1. Miriam Wettstein, Head of patient advisory board, Inselspital, Nursing Directorate<br>2. Jacques Andres, Fribourg, Patient<br>3. Beatrice Christener, Niederwangen BE, Patient<br>4. Sara Jetzer, Bern, Patient<br>5. Bertrand Mauron, Bellmund, Patient
**Patient advisor, representative of the Krebsliga Schweiz**	6. Sefan Gremminger, Diepoldsau, Patient
**Patient advisors, representatives of the Selbsthilfenetzwerk Kopf-Hals-M.U.N.D.-Krebs e.V, Bonn, Deutschland**	7. Gunthard Kissinger, Head of the Selbsthilfenetz and Patient<br>8. Ingetraud Bönte-Hieronymus, Associate member and Patient<br>9. Jörg Hennigs, Associate member and Patient

Data Monitoring Committee (DMC)

To be defined
